# Synthesis and Reactivity of Extremely Electron‐Poor Au(III) Complexes Bearing OTf^−^ or NTf_2_^−^ Ligands

**DOI:** 10.1002/chem.70920

**Published:** 2026-03-27

**Authors:** Lachlan Barwise, Emily Mulhallen‐Graham, Lachlan J. Moon, Lachlan Sharp‐Bucknall, Miracle I. Ekavhiare, Keith F. White, Jason L. Dutton

**Affiliations:** ^1^ Department of Biochemistry and Chemistry, La Trobe Institute for Molecular Science La Trobe University Melbourne Victoria Australia

## Abstract

The synthesis and structural characterization of well‐defined Au(III)‐triflate and ‐bistriflimide complexes in concert with methylimidazole ligands are reported. These extremely electron‐poor gold complexes are found to be susceptible to aryl metalation reactions with unfunctionalized aryls such as benzene and toluene. Subsequent addition of soluble halide sources allows for the synthesis of aryl halides.

## Graphical abstract

A new family of electron‐poor Au(III) cations bearing bistriflimide or triflate ligands is reported. These cations can undergo direct C─H metalation with simple arenes to give mono‐aryl cations. Subsequent addition of simple halide sources allows for reductive elimination of aryl halides, formally from Ar─H and X^−^.

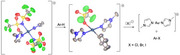

## 1 Introduction

With a later start in organometallic catalysis than many other metals [1], the elementary processes around gold for the Au(I)/Au(III) redox couple remain relatively less explored [2]. This is due to the reluctance of Au(I) to engage in many of the traditional oxidative addition processes, which less electronegative metals with non‐d‐10 electron counts undergo [3]. Nonetheless, in the past decade, strategies have emerged to achieve oxidative additions, and once in the +3 oxidation state, the relatively high reactivity this electron configuration presents for gold can be taken advantage of [4, 5, 6, 7, 8]. Our interest in this area lies in gaining an understanding of the reactivity of Au in the +3 oxidation state, and specifically what is possible if the already electron‐hungry d‐8 gold is rendered more electron‐poor by incorporating poorly nucleophilic or electron‐withdrawing ligands. We have generated Au(III) complexes that include trications featuring four pyridine or imidazole‐type ligands [9, 10] and more electron‐poor complexes in which two imidazoles are replaced with acetonitrile [11]. We have also explored using fluoride as a ligand, with two fluorides trans to one another about Au(III) [12].

**SCHEME 1:**
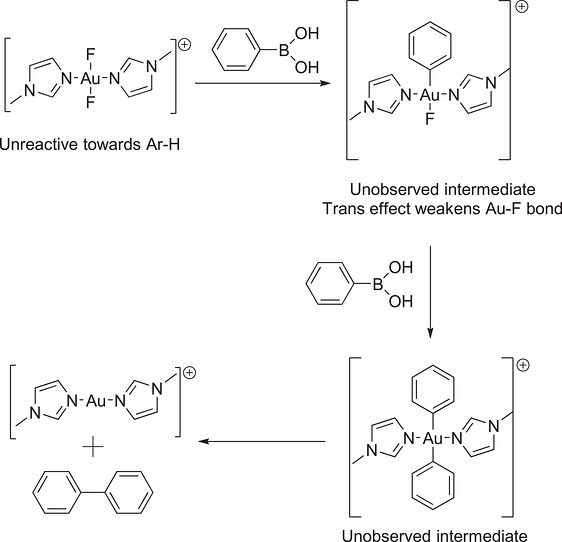
Reaction of [Au(MeIM)_2_F_2_]^+^ with phenylboronic acid, resulting in an undesired double substitution and reductive elimination of biphenyl.

These compounds have shown efficacy in generating other Au─E compounds via direct E─H activation with a variety of substrates bearing protons having a range of acidities [13]. A limitation of the reactions arises from the weak trans influence and the basicity of the pyridine or fluoride ligands, resulting in a general inability to perform single E─H activations at gold [14]. The new E─Au bond exerts a strong trans influence, rendering the Au─L or Au─F bond opposite weaker and more reactive toward E─H substitution, resulting in the metalation reactions occurring in pairs. Additionally, C─H substrates, with the goal of effecting C─H to C─E transformations, react either sluggishly or not at all. Metalating with aryl boronic acids on Au─F was possible, but again two metatheses occur, with subsequent reductive elimination to a biaryl (Scheme 1) [14].

The bisacetonitrile complex was, however, able to perform a single C─H activation of mesitylene (over 4 days), where, despite the much stronger trans influence of mesitylene as compared to acetonitrile, the poor basicity of acetonitrile suppressed a second C─H metalation [11].


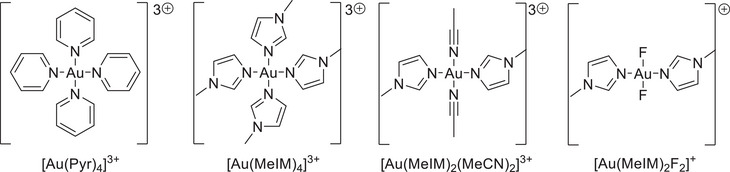


In this work, we report the synthesis of an Au(III) cation bearing poorly nucleophilic triflate or bistriflimide ligands, which provide the ideal mixture of an electron‐poor Au(III) effective for rapid aryl C‐H metalation and lability to introduce a second substrate, inducing reductive elimination and C─X bond formation.

## 2 Results and Discussion

Our initial target in this study was [Au(MeIM)_2_(OTf)_2_][OTf], with two imidazole and two OTf^−^ ligands bound to the Au(III). There are only two examples of structurally characterized Au(III) complexes, including a triflate ligand bound to the gold, one featuring a phenylpyridine and methyl filling the other three coordination sites [15], and the other with two chlorides and a water [16]. In the former example from 2011, it was noted that Au(III) complexes bearing highly labile anionic ligands are very rare and potentially useful for studying catalytic intermediates on the Au(III) side of Au(I)/Au(III) redox processes.

**SCHEME 2:**
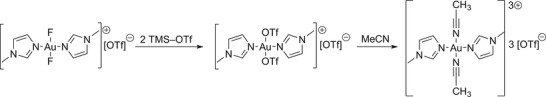
Reaction of [Au(MeIM)_2_F_2_][OTf] with 2 TMS‐OTf giving proposed intermediate [Au(MeIM)_2_(OTf)_2_][OTf] and onward substitution with CH_3_CN.

Reaction of a suspension of [Au(MeIM)_2_F_2_][OTf] with two equivalents of TMS‐OTf in CDCl_3_ resulted in an immediate color change of the solids from pale yellow to bright yellow (Scheme 2). The ^1^H NMR spectrum showed complete conversion of the TMS‐OTf into TMS‐F, and in the aryl region of the spectrum, only traces of Au(I) [Au(MeIM)_2_]^+^ were present. The solids were isolated and found to be soluble in CD_3_CN. The ^19^F NMR spectrum showed an absence of any resonance consistent with an Au‐F and a clear signal consistent with OTf^−^. The ^13^C NMR spectrum, however, was the same as the [Au(MeIM)_2_(MeCN)_2_]^3+^ complex previously reported by our group as a [BF_4_]^−^ salt [11]. Single crystals were grown from a sample stored at −30°C and x‐ray diffraction studies confirmed the compound to be Au(MeIM)_2_(MeCN)_2_][OTf]_3_. We hypothesize that the insoluble yellow powder produced in the reaction with TMS‐OTf to be [Au(MeIM)_2_(OTf)_2_][OTf], but cannot confirm this as the compound decomposes in any solvent in which it dissolves, except for MeCN, which gives the bisacetonitrile complex.

If one equivalent of TMS‐OTf is used, a new compound with an Au─F ^19^F chemical shift of −248 ppm is observed (c.f. [Au(MeIM)_2_F_2_][OTf] at −284 ppm), with a corresponding downfield shift for the imidazole protons in the ^1^H NMR. We believe this to be a mono‐fluoro complex of the Au(III); unfortunately, crystallographic characterization to identify if the fourth ligand is acetonitrile or triflate has remained elusive.

The apparent displacement of the OTf^−^ ligands from the Au(III) center by MeCN in the reaction with two equivalents of TMS‐OTf caught our interest, as [Au(MeIM)_2_(MeCN)_2_]^3+^ is an extremely electron‐poor compound, and this ligand exchange suggests [Au(MeIM)_2_(OTf)_2_]^+^ is even more electron‐poor. To access a similar compound with hopefully improved solubility, the same method was attempted using TMS‐NTf_2_. In iodine(III) chemistry, we have previously found that the NTf_2_^−^ bound I(III) centers in ArIL_2_ type compounds display similar properties as when the ligand is OTf^−^, albeit with NTf_2_^−^ compounds being even more oxidizing [17], as NTf_2_^−^ is a poorer nucleophile than OTf^−^ [18].

Reaction of [Au(MeIM)_2_F_2_][OTf] with two equivalents of TMS‐NTf_2_ in CD_2_Cl_2_ resulted in an orange precipitate within 1 min. The ^19^F NMR spectrum of the solids showed the absence of the Au─F signal from [Au(MeIM)_2_F_2_][OTf]. In the ^1^H NMR spectrum, the resonances from the MeIM ligands were observed to be downfield of the corresponding signals in [Au(MeIM)_2_F_2_][OTf], indicative of the generation of a more electron‐poor species. However, multiple resonances arising from MeIM were present. If four equivalents of TMS‐NTf_2_ are used, a single major product is, however, observed.

**SCHEME 3:**
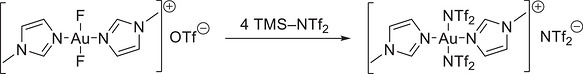
Reaction of [Au(MeIM)_2_F_2_][OTf] with excess TMS‐NTf_2_ giving [Au(MeIM)_2_(NTf_2_)_2_][NTf_2_].

**FIGURE 1:**
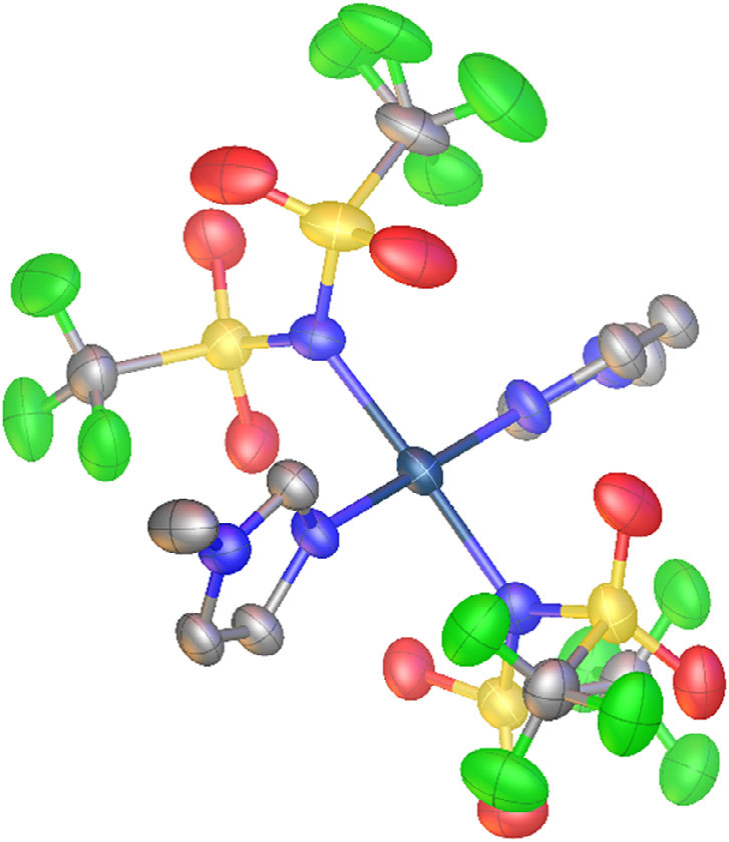
Solid‐state structure of [Au(MeIM)_2_(NTf_2_)_2_][NTf_2_]. Thermal ellipsoids are shown to 50% probability level. The [NTf_2_] counterion and hydrogen atoms are omitted.

Single crystals were grown from the NMR solution of the reaction using four equivalents of TMS‐NTf_2_ at −30°C in the glovebox freezer. Subsequent x‐ray diffraction studies revealed the compound to be [Au(MeIM)_2_(NTf_2_)_2_][NTf_2_] (Scheme 3 and Figure 1). Attempted isolation of a bulk solid consistently resulted in substantive decomposition to Au(I), thus, the [Au(MeIM)_2_(NTf_2_)_2_]^+^ cation in subsequent reactivity studies is generated and used in situ.

There is only one other structurally characterized class of Au(III) bound NTf_2_, two NHC‐AuCl_2_(NTf_2_) complexes where the NTf_2_ is trans to the NHC ligands [19]. The Au─N distances in those two complexes are 2.11 and 2.17 Å. The Au─N distances in [Au(MeIM)_2_(NTf_2_)_2_][NTf_2_] are slightly shorter at 2.050(6) and 2.061(6) Å for two NTf_2_ trans to one another. There are no significant contacts with the gold in the axial octahedral positions. We hypothesize that in the reaction with two equivalents of TMS‐NTf_2,_ the remaining triflate anion competes with [NTf_2_]^−^ at the Au(III) center resulting in a distribution of products.

**FIGURE 2:**
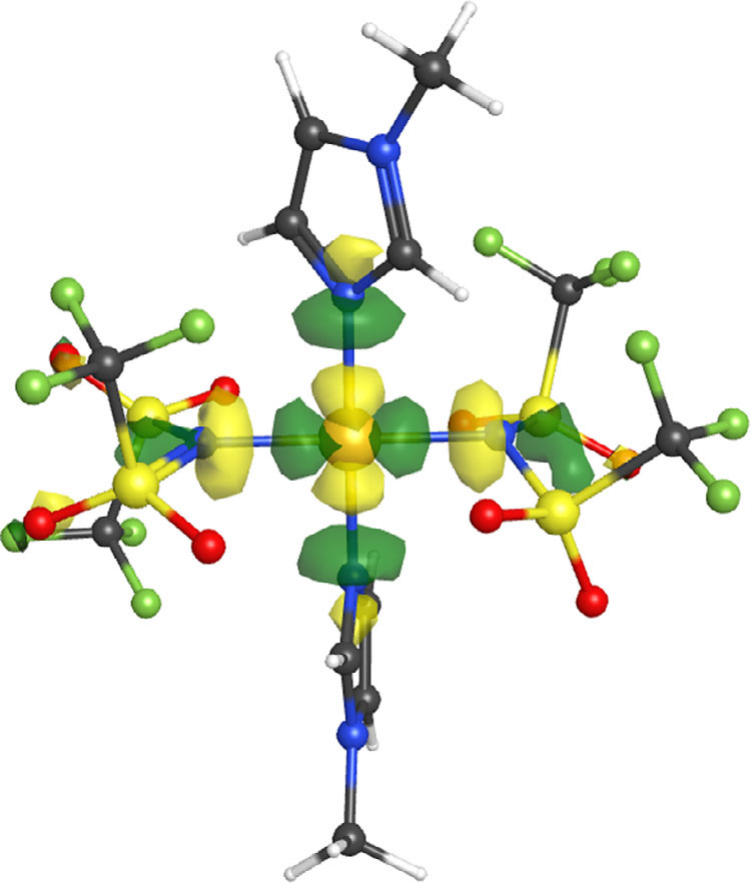
LUMO of [Au(MeIM)_2_(NTf_2_)_2_]^+^.

The electronic structure of [Au(MeIM)_2_(NTf_2_)_2_]^+^ was investigated using theoretical studies. The geometry was optimized using ωHPBE/def2SVP, which reproduced the experimentally determined metrical parameters well. Molecular orbitals were calculated using B3LYP/def2TZVP on the optimized geometry. The nature of the LUMO is similar to all other Au(III) compounds in the family, with a sigma antibonding interaction along the Au─N bond axes (Figure 2). The energy level of the LUMO is calculated to be −6.87 eV. The energy level of the LUMO in the [Au(MeIM)_2_F_2_]^+^ precursor is higher at −6.20 eV. Comparison to the more closely related Au‐OAc complex [Au(MeIM)_2_(OAc)_2_]^+^ shows this cation has a LUMO energy level of −6.09 eV. The more energetically accessible LUMO of [Au(MeIM)_2_(NTf_2_)_2_]^+^ potentially points to increased electrophilic behavior. The electron affinity for the addition of two electrons to [Au(MeIM)_2_(NTf_2_)_2_]^+^ was calculated to be 638 kJ/mol, higher than what is calculated for [Au(MeIM)_2_(OTf)_2_]^+^ at 597 kJ/mol. [Au(MeIM)_2_(OAc_2_)_2_]^+^ has a calculated two‐electron affinity of 302 kJ/mol. The LUMO for [Au(MeIM)_2_(OTf)_2_]^+^ has similar character to [Au(MeIM)_2_(NTf_2_)_2_]^+^ and is further lower in energy than [Au(MeIM)_2_(NTf_2_)_2_]^+^ at −7.04 eV, making it ambiguous as to which analogue would be predicted to be more electron‐poor between NTf_2_^−^ and OTf^−^ ligated, but both are clearly more electrophilic than previously reported [Au(MeIM)_2_(OAc_2_)_2_]^+^.

As with our previously reported tricationic acetonitrile complex, [Au(MeIM)_2_(MeCN)_2_][OTf]_3_,^11^ an experimental effort to support this assertion using electrochemistry was thwarted due to the oxidation potential being outside the range of any solvent that [Au(MeIM)_2_(NTf_2_)_2_]^+^ dissolves in but does not react with. However, as with [Au(MeIM)_2_(MeCN)_2_]^3+^, addition of the electrochemical reference compound [Ru(bpy)_3_][BF_4_]_2_ (bpy = bipyridine) to [Au(MeIM)_2_(NTf_2_)_2_]^+^ results in an immediate change to the characteristic green color of [Ru(bpy)_3_]^3+^ [20], indicating that [Au(MeIM)_2_(NTf_2_)_2_]^+^ is more oxidizing than [Ru(bpy)_3_]^3+^.

### 2.1 Reactivity Studies

Aryl C‐H activation by [Au(MeIM)_2_(MeCN)_2_]^3+^ was limited in scope to only mesitylene and was sluggish, with the reaction giving [Au(MeIM)_2_(MeCN)(Mes)]^2+^ taking four days at room temperature to reach completion [11]. In contrast, the reaction of one equivalent of mesitylene with [Au(MeIM)_2_F_2_][OTf] in the presence of two equivalents of TMS‐NTf_2_, generating [Au(MeIM)_2_(NTf_2_)_2_]^+^ in situ, in CD_2_Cl_2_, resulted in an instant color change to black. The ^1^H NMR showed a complex mixture including a set of peaks matching the Au(I) cation [Au(MeIM)_2_]^+^. ^1^H NMR resonances consistent with coupled bimesitylene were also apparent [21]. We have previously observed such behavior in the reaction of [Au(MeIM)_2_F_2_]^+^ with aryl boronic acids, where the more nucleophilic aryl moiety in that reagent resulted in a double aryl addition, followed by reductive elimination of biaryl [14]. Given the extremely fast reaction, we reasoned that the predicted more reactive [Au(MeIM)_2_(NTf_2_)_2_]^+^ might be too reactive with mesitylene and a similar double metalation might be occurring. Therefore, reactions were carried out with less activated benzene or toluene as substrates.

**SCHEME 4:**
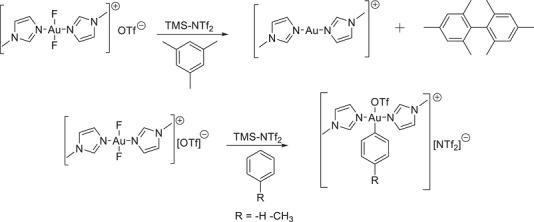
Reaction of [Au(MeIM)_2_F_2_][OTf] with two TMS‐NTf_2_ in the presence of mesitylene, toluene, and benzene.

**FIGURE 3:**
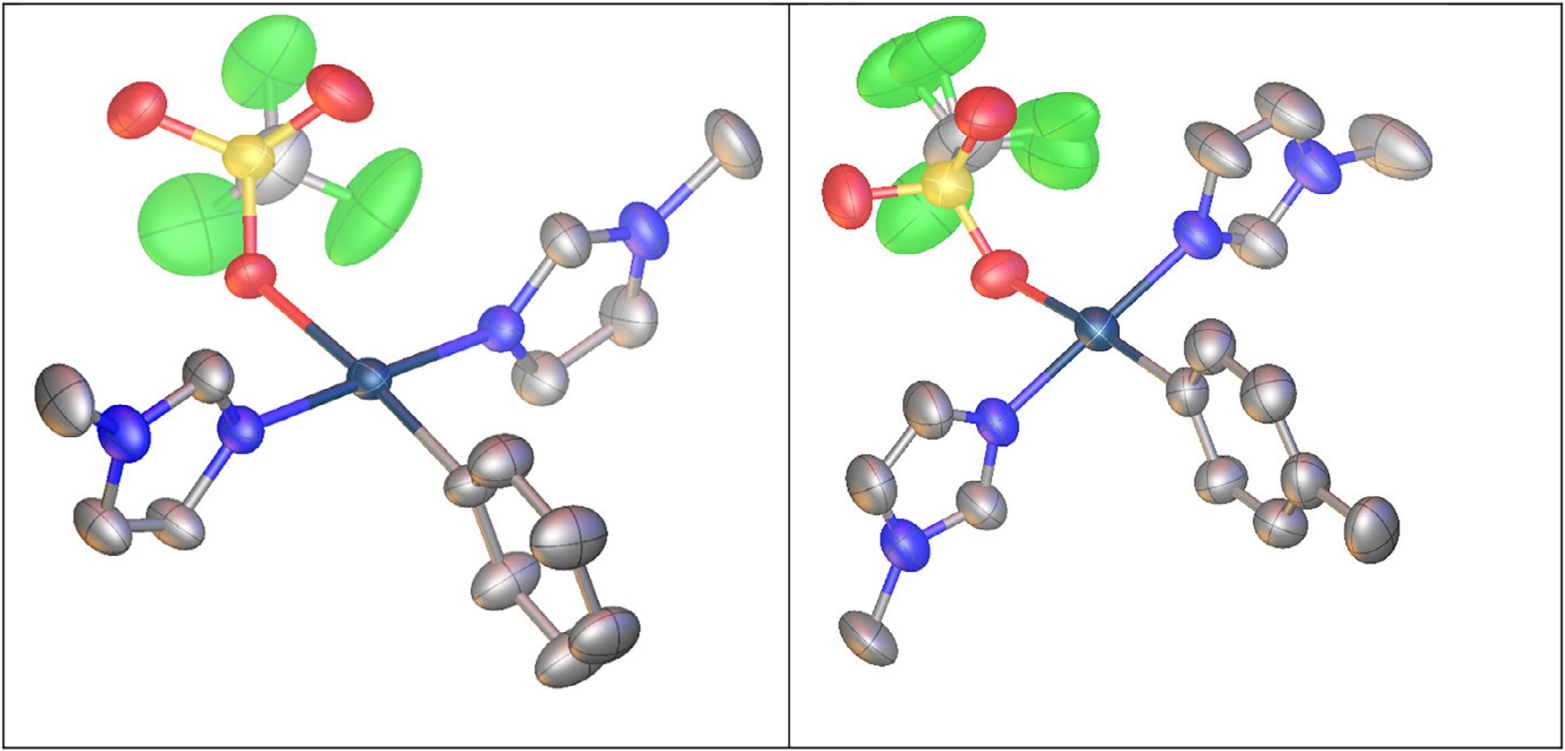
Solid state structures of [Au(MeIM)_2_(C_6_H_5_)(OTf)][NTf_2_] and [Au(MeIM)_2_(C_6_H_4_(CH_3_))(OTf)][NTf_2_]. Thermal ellipsoids are shown to 50% probability level. Hydrogen atoms and counterions are omitted.

Reactions of [Au(MeIM)_2_F_2_][OTf] with one equivalent of benzene or toluene in the presence of two equivalents of TMS‐NTf_2_ in CD_2_Cl_2_ resulted in a gradual change to yellow over 16 h. Dichloromethane is preferred over chloroform as the solubility of the [Au(MeIM)_2_(NTf_2_)_2_]^+^ complex is higher, allowing the reaction to better outpace product decomposition. For the same reason, [Au(MeIM)_2_(NTf_2_)_2_]^+^ is superior for the study of these reactions over [Au(MeIM)_2_(OTf)_2_]^+^, attempts at in situ reactions with TMS‐OTf as the fluoride abstraction agent resulted in very sluggish reactions and almost exclusively decomposition to Au(I). ^1^H NMR spectroscopy indicated that [Au(MeIM)_2_(NTf_2_)_2_]^+^ was being consumed and a new product emerging. [Au(MeIM)_2_(NTf_2_)_2_]^+^ was completely consumed upon stirring at room temperature overnight for both substrates. The ^1^H NMR showed sets of resonances consistent with a single‐site metalation as the major product in both cases. Single crystals were grown by concentration of the CD_2_Cl_2_ solutions of each reaction mixture by vapor diffusion into *n*‐hexane. X‐ray diffraction studies showed that for both reactions, the product was monoaryl metalated Au(III) with two imidazoles and a triflate filling the coordination sphere. For the benzene analogue, the counterion is NTf_2_^−^, whereas for toluene, OTf^−^, NTf_2_^ȡ^ and a [NTf_2_‐Au‐NTf_2_]^−^ are all present in the asymmetric unit, with the latter Au(I) anion indicative of some decomposition process having occurred (Scheme 4 and Figure 3). Decomposition upon attempted purification precluded the isolation of analytically pure samples in both cases. Reactions with less activated aryls containing formal electron withdrawing groups resulted in more sluggish reactions that were unable to outpace the intrinsic decomposition to Au(I) that [Au(MeIM)_2_(NTf_2_)_2_]^+^ suffers from in solution.

**SCHEME 5:**
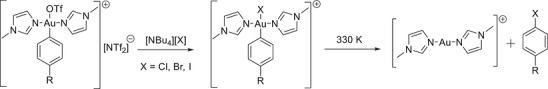
Reaction of [Au(MeIM)_2_(Ar)(OTf)][NTf] (Ar = benzene, toluene) with [NBu_4_][X] (X = Cl, Br, I), followed by heating, results in the reduction elimination of haloarenes.

**FIGURE 4:**
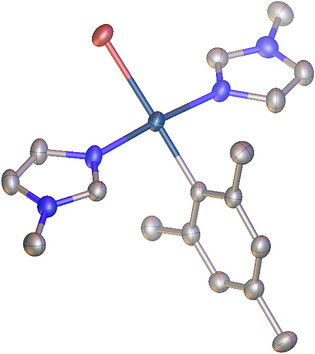
Solid state structure of [Au(MeIM)_2_(Br)(Mes)][OTf]. Thermal ellipsoids are depicted at 50% probability level. Hydrogen atoms and [OTf]– counterion are omitted.

Having been able to perform a mono‐metalation with a good leaving group still in place in NTf_2_^−^ or OTf^−^ we next sought to investigate if productive bond‐forming reactions were possible at the Au(III) center. These processes are of interest in the context of the reductive elimination manifold in Au(I)/Au(III) catalysis, which has been studied less systematically than the oxidative addition side of such cycles [15]. Halides were decided to be a good model nucleophile for this, with a goal of generating halo‐arenes, which have been of interest with Au(III) chemistry [22]. Reactions were found to be most convenient to carry out by reacting [Au(MeIM)_2_F_2_][OTf] with two equivalents of TMS‐NTf_2_ in CD_2_Cl_2_, to allow for in situ NMR monitoring, followed by addition of one equivalent of arene (benzene or toluene), allowing to react overnight to complete metalation and then addition of [NBu_4_][X] as a soluble halide source (Scheme 5). Upon addition of [NBu_4_][X] (X = Cl, Br, I), the ^1^H NMR spectra showed an immediate change to a new compound, still containing two methylimidazole ligands and the arene by integration. Despite extensive efforts, we were unable to grow single crystals of sufficient quality for x‐ray diffraction studies of these products. However in displacement of MeCN from [Au(MeIM)_2_(MeCN)(Mes)][OTf]_2_ with [NBu_4_][Br] the product was able to be verified with x‐ray crystallography (Figure 4), giving [Au(MeIM)_2_(Br)(Mes)][OTf], along with mass spectrometry returning signals consistent with the targets across the halide series (Cl, Br, I), confirming the viability of the [Au(MeIM)_2_(X)(arene)]^+^ compound family.

If these compounds are heated modestly to 55°C, in most cases for a few days, generation of [Au(MeIM)_2_]^+^ is observed, with reductive elimination of halo‐arenes. Higher temperatures to reduce reaction times led to a myriad of decomposition products.

**SCHEME 6:**
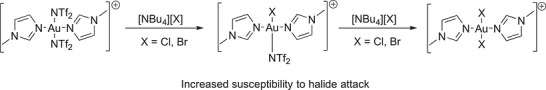
Reaction of [Au(MeIM)_2_(NTf_2_)_2_][NTf_2_] with [NBu_4_][X], ultimately resulting in disubstitution.

The order of addition for this process was found to be important. If [Au(MeIM)_2_(NTf_2_)_2_]^+^ is reacted with [NBu_4_][Cl] or [NBu_4_][Br], regardless of stoichiometry, [Au(MeIM)_2_X_2_]^+^ is formed, which was crystallographically verified for bromide and by NMR compared to a genuine sample for chloride (Scheme 6). With this order of addition, the increased trans influence of the halide relative to NTf_2_^−^ renders [Au(MeIM)_2_(NTf_2_)(X)]^+^ more reactive and the preferential substrate for a second substitution. Therefore, the order of reaction for benzene or toluene halogenation using this system is arene metalation, followed by halide addition.

**SCHEME 7:**
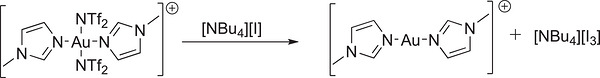
Reaction of [Au(MeIM)_2_(NTf_2_)_2_][NTf_2_] with [NBu_4_][I] resulting in iodide oxidation.

If [NBu][I] is added to [Au(MeIM)_2_(NTf_2_)_2_]^+^, the reaction mixture immediately turns deep red. ^1^H NMR showed the presence of [Au(MeIM)_2_]^+^ amongst other signals, indicating a redox process of some kind had occurred. Storing the reaction mixture in the freezer resulted in the growth of single crystals, and following x‐ray diffraction, the structure of the crystals was shown to be [NBu_4_][I_3_] (Scheme 7). This result suggests that [Au(MeIM)_2_(NTf_2_)_2_]^+^, when exposed to I^−^, oxidizes the iodide, ultimately resulting in a complex mixture of products. This result demonstrates that compounds of the class [Au(MeIM)_2_I_2_]^+^ or with related nitrogen ligands are likely inaccessible, as the iodide will be oxidized. Computation analysis shows that the reductive elimination of I_2_ from this compound has a Δ*G* of −63 kJ/mol, supporting this conclusion. For isolable [Au(MeIM)_2_Br_2_]^+^, the corresponding calculated Δ*G* for reductive elimination of Br_2_ is essentially flat at −3 kJ/mol.

It was found that halogenation of more activated mesitylene by [Au(MeIM)_2_(NTf_2_)_2_][NTf_2_] could be accomplished by mixing mesitylene and [NBu_4_][X] in CD_2_Cl_2_ solvent, followed by addition to a cold (232 K) solution of [Au(MeIM)_2_(NTf_2_)_2_][NTf_2_], which resulted in rapid reaction and formation of chloro‐, bromo‐ or iodo‐mesitylene. This was found to be most efficient for iodination, followed by bromination, and finally for chlorination, while conversion to chloromesitylene was poor, and several other unidentified products were apparent in the mixture.

The mechanism for the addition of halides to the metalated complexes was not conclusively determined, but in both [Au(MeIM)_2_(C_6_H_5_)(OTf)][NTf_2_] and [Au(MeIM)_2_(C_6_H_4_(CH_3_))(OTf)][NTf_2_], short contacts of ∼ 3 Å are observed in the axial positions with NTf_2_^−^ counterions in the solid state. It is likely that the much more nucleophilic halides initially engage with the Au(III) along this axis and then displace the bound Au‐OTf unit. These types of interactions have been observed in other cationic Au(III) complexes [23, 24]. They are not observed in [Au(MeIM)_2_(NTf_2_)_2_][NTf_2_], likely due to the steric constraints about Au imposed by the presence of two bound NTf_2_^−^ ligands.

## 3 Conclusion

In conclusion, we have described the synthesis of a new family of electron‐poor Au(III) cations bearing triflate or bistriflimide ligands. The new compounds allow for single C─H arylation reactions with isolation of the resultant metalated organometallic species. These reactions are somewhat hampered by a propensity for decomposition to Au(I), which is an issue in the chemistry of reactive Au(III) species, but nonetheless result in the rare examples of unsupported monoarylation of Au(III) directly from C─H aryls. The specific mechanism was not elucidated in this study, but it is likely via the S_E_Ar process proposed for the few other similar reactions known [25]. These can then be used for onward bond‐forming reactions with simple halide sources, resulting in the synthesis of aryl halides, formally from C─H aryls and X^−^. The observation that relatively weak nucleophiles such as acetonitrile and unactivated C─H arenes can displace the triflate and bistriflimide ligands means that other relatively unreactive substrates might be susceptible to activation by this family of compounds and could potentially be a useful synthon for Au(III) chemistry more widely.

## Conflicts of Interest

The authors declare no conflicts of interest.

## Supporting information



Experimental procedures, NMR and mass spectra, x‐ray crystallographic details, computational methods. Figures S1–S48, Tables S1–S7.
