# Supplementary material for: Synthesis and Reactivity of Extremely Electron‐Poor Au(III) Complexes Bearing OTf− or NTf2 − Ligands
Source: Chemistry. 2026 Mar 27;32(22):e70920. doi: 10.1002/chem.70920 (PMC13250368; doi:10.1002/chem.70920)
Supplement: Supplementary file 1 — Experimental procedures, NMR and mass spectra, x‐ray crystallographic details, computational methods. Figures S1–S48, Tables S1–S7. [file CHEM-32-e70920-s001.pdf]

Electronic Supplementary Information for Synthesis and Reactivity of Extremely Electron Poor Au(III) Complexes Bearing OTf^- or NTf_2^- Ligands

Lachlan Barwise, Emily Mulhallen-Graham, Lachlan Moon, Lachlan Sharp-Bucknall, Miracle I. Ekavhiare, Keith F. White and Jason L. Dutton*

Department of Biochemistry and Chemistry, La Trobe Institute for Molecular Science, La Trobe University, Melbourne, Victoria, Australia

j.dutton@latrobe.edu.au

Contents

General Procedures 2
Experimental 3
X-Ray Crystallographic Data 11
NMR Spectra 19
Mass Spectra: 56
Computational Details: 58
References 68

General Procedures

All reactions were performed under an N_2 atmosphere in either a glovebox or using Schlenk techniques. Glovebox solvents were dried using an Innovative Technologies Solvent Purification System. The dried solvents were stored under N_2 atmosphere over 3 Å molecular sieves in the glovebox. Deuterated solvents for NMR spectroscopy were purchased from Sigma Aldrich and dried by stirring for three days over CaH_2, distilled prior to use, and stored in the glovebox over 3 Å molecular sieves. Synthesis of THT-AuCl from gold powder (obtained from Precious Metals Online) was performed by literature procedure as well as the syntheses of [Au(MeIM)$_2$F$_2$][OTf], [Au(MeIM)$_2$][OTf].[1-3.]

All other reagents were purchased from Sigma Aldrich and used as received. Glassware was dried in an oven at 120 °C overnight and transferred to the glovebox port or Schlenk line where it was subjected to three vacuum cycles over 30 minutes prior to use. NMR spectra for all experiments were recorded using Bruker Ultrashield Plus 500 MHz and Ascend 400 MHz spectrometers.

Experimental

Reaction of $[Au(MeIM)_2F_2][OTf]$ with 2 equivalents of TMS-NTf_2

To a solution of $[Au(MeIM)_2F_2][OTf]$ (16 mg, 0.029 mmol) in CH_2Cl_2, 2 equivalents of TMS-NTf_2 (20 mg, 0.058 mmol) was added. The solution turned orange and after 1 minute orange/yellow precipitate formed. The precipitate was washed with cold CH_2Cl_2 and hexane, the suspension was left to sit till all precipitate had sedimented and the was solvent was carefully removed by pipette before exposing to vacuum for a few seconds to help dry the solid.

^1H NMR (400 MHz, (CD_2Cl_2)) δ (ppm): 4.00 (s, 6H), 4.11 (s, 4.5H), 7.31 (s, 2H), 7.48 (s, 2H), 7.54 (s, 2H), 7.61 (s, 1.5H), 8.35 (s, 1.5H), 8.73 (s, 2H).

^{19}F NMR (376 MHz, (CD_2Cl_2)) δ (ppm): -79.30 (s), -71.17 (s) -71.05 (s).

Synthesis of $[Au(MeIM)_2(NTf_2)_2][NTf_2]$

The method follows the same procedure as the previous entry, however 4 equivalents of TMS-NTf_2 are added to remove the OTf^- anion as TMS-OTf. Attempts to isolate a bulk solid resulted in increasing amounts of $[Au(MeIM)_2]^+$, worsening upon any purification attempts, therefore the compound is best used *in situ*.

^1H NMR (400 MHz, (CD_2Cl_2)) δ (ppm): 4.11 (s, 6H), 7.54 (s, 2H), 7.59 (s, 2H), 8.34 (s, 2H).

^{19}F NMR (376 MHz, (CD_2Cl_2)) δ (ppm): -78.02 (s), -77.52 (s), -71.14 (s).

$^{13}C\{^1H\}$ NMR (126 MHz, (CD_2Cl_2)) δ (ppm): 37.6 (s), 125.6 (s), 127.5 (s), 129.9 (s), 137.8 (s).

A single crystal suitable for X-ray diffraction was grown by vapour diffusion at -40°C with CD_2Cl_2/ n-hexanes.

Generation of $[Au(MeIM)_2(MeCN)_2][OTf]_3$

To a sample of $[Au(MeIM)_2F_2][OTf]$ (10 mg, 0.018 mmol) cooled to near freezing in a cold well by N_2(l) in CD_3CN, 4 equivalents of TMS-OTf (16 mg, 0.075 mmol) was added, the product of $[Au(MeIM)_2(MeCN)_2][OTf]_3$ was identified by X-ray diffraction quality crystals grown in by slow precipitation at -40 °C by adding 1 equivalent (by volume) of n-hexane to CD_3CN before CH_2Cl_2 was dropped in until the bilayer disappeared and before the

reaction mixture turned cloudy (more MeCN can be added dropwise to prevent early fast precipitation).

^{1}H NMR (400 MHz, (CD_2Cl_2)) δ (ppm): 3.95 (s, 6H), 7.48 (s, 2H), 7.53 (s, 2H). 8.51 (s, 2H).

^{19}F NMR (376 MHz, (CD_2Cl_2) δ (ppm): -79.14 (s)

^{13}C{^{1}H} NMR (126 MHz, (CD_2Cl_2)) δ (ppm): 37.1 (s), 124.9 (s), 127.6 (s), 140.2 (s).

Reaction of [Au(MeIM)$_2$F$_2$][OTf] with 1 equivalent of TMS-OTf

To a solution of [Au(MeIM)$_2$F$_2$][OTf] (13 mg, 0.025 mmol) in CD_3CN, 1 equivalent of TMS-OTf (5 mg, 0.025 mmol) was added. The mono-fluorinated Au product was identified by ^{1}H and ^{19}F NMR:

^{1}H NMR (400 MHz, (CD_3CN)) δ (ppm): 3.90 (s, 6H), 7.38 (s, 2H), 7.41 (s, 2H), 8.41 (s, 2H).

^{19}F NMR (376 MHz, (CD_3CN)) δ (ppm): -248.31 (s), -79.28 (s).

Synthesis of [Au(MeIM)$_2$Br$_2$][OTf]

A solution of [Au(MeIM)$_2$F$_2$][OTf] (49 mg, 0.09 mmol) dissolved in MeCN (2 mL) was reacted with 2 equivalents of TMS-Br (27 mg, 0.18 mmol). Colour change was observed from colourless to orange as the solution was stirred for 10 minutes. The orange solution was dried *in vacuo* to give an orange solid. (45 mg, 75% yield).

Single crystals suitable for X-ray diffraction were grown by vapour diffusion of MeCN/Et_2O.

^{1}H NMR (400 MHz, (CD_3CN)) δ (ppm): 3.86 (s, 6H), 7.33 (s, 2H), 7.39 (s, 2H), 8.25 (s, 2H).

^{19}F NMR (376 MHz, (CD_3CN)) δ (ppm): -79.34 (s).

ESI-MS: m/z(+) [Au(MeIM)$_2$Br$_2$]$^+$ 520.9 (calc. 518.91).

Reaction of [Au(MeIM)$_2$(NTf$_2$)$_2$][OTf] with [NBu$_4$][I]

To a solution of [Au(MeIM)$_2$(NTf$_2$)$_2$][OTf], (31 mg, 0.029 mmol) 2 equivalents of [NBu$_4$][I] (21 mg, 0.058 mmol) was added, the solution turned brown/red colour, [Au(MeIM)$_2$]$^+$ and free methylimidazole were identified by ^{1}H NMR, and the products of [NBu$_4$][I$_3$] were identified by X-ray crystallography.

Single crystals of [NBu$_4$][I$_3$] suitable for X-ray diffraction was grown by vapour diffusion at -40 °C with CD_2Cl_2/n-hexanes.

Synthesis of $[Au(MeIM)_2(C_6H_5)(OTf)][NTf_2]$

To a solution of $[Au(MeIM)_2(NTf_2)_2][OTf]$ generated *in-situ* in CD_2Cl_2 by reacting $[Au(MeIM)_2F_2][OTf]$ (7 mg, 0.013 mmol) with 2 equivalents of TMS-NTf_2 (9 mg, 0.026 mmol), 1 equivalent of benzene was added (1 mg, 0.013 mmol) and left to stir overnight. The resulting product was completely redissolved after 16 hours offering a yellow solution. A white precipitate was isolated with the addition of 1:1 Hex/Et_2O (3 mg, 27% yield) however could not be separated from small amounts of decomposition products.

Vapour diffusion at -40 °C with CD_2Cl_2/n-hexanes produced single crystals identified as $[Au(MeIM)_2(C_6H_5)(OTf)][NTf_2]$ by X-ray diffraction.

^{1}H NMR (400 MHz, (CD_2Cl_2)) δ (ppm): 3.82 (s, 6H), 7.14 (m, 4H), 7.15 (m, 2H). 7.16 (m, 2H), 7.27 (m, 1H), 8.12 (s, 2H).

^{19}F NMR (376 MHz, (CD_2Cl_2) δ (ppm): -77.97 (s,), -75.46 (s), -75.11 (s), -71.21 (s).

^{13}C{^{1}H} NMR (126 MHz, (CD_2Cl_2)) δ (ppm): 36.3 (s), 118.5 (s), 121.1 (s), 123.0 (s), 127.6 (s), 128.9 (s), 130.1 (s), 131.7 (s), 139.4 (s).

Synthesis of $[Au(MeIM)_2(C_6H_4(CH_3))(OTf)][NTf_2]$

To a solution of $[Au(MeIM)_2(NTf_2)_2][OTf]$ generated *in-situ* in CD_2Cl_2 by reacting $[Au(MeIM)_2F_2][OTf]$ (7 mg, 0.013 mmol) with 2 equivalents of TMS-NTf_2 (9 mg, 0.026 mmol), 1 equivalent of toluene was added (1 mg, 0.012 mol) and left to stir overnight. The resulting product was completely redissolved after 16 hours offering a rich golden solution. A white precipitate was isolated with the addition of 1:1 n-hexanes/Et_2O (4 mg, 34% yield) identified as $[Au(MeIM)_2(C_6H_4(CH_3))(OTf)][NTf_2]$ by NMR, however, could not be separated from small amounts of decomposition products.

A single crystal suitable for X-ray diffraction was grown by vapour diffusion at - 40°C with CD_2Cl_2/n-hexanes.

^{1}H NMR (400 MHz, (CD_2Cl_2)) δ (ppm): 2.34 (s, 3H), 3.82 (s, 6H), 6.98 (dd, 4H), 7.13 (d, 2H), 7.14 (d, 2H), 8.10 (s, 2H).

^{19}F NMR (376 MHz, (CD_2Cl_2)) δ (ppm): -78.47 (s), -78.15 (s), -75.45 (s), -75.10 (s), -72.45 (s).

^{13}C{^{1}H} NMR (126 MHz, (CD_2Cl_2)) δ (ppm): 20.5 (s), 21.6 (s), 35.9 (s), 122.3 (s), 127.3 (s), 128.0 (s), 128.4 (s) 129.2 (s), 131.9 (s), 139.5 (s).

Synthesis of [Au(MeIM)$_2$(I)(Mes)][OTf]

To a solution of [Au(MeIM)$_2$(MeCN)(Mes)][OTf]$_2$ (14 mg, 0.018 mmol) in CD_3CN, 1 equivalent of [NBu$_4$][I] (6 mg, 0.018 mmol) was added. The solution turned bright orange immediately, slowly turning red, producing a yellow precipitate.

^{1}H NMR (400 MHz, (CD_3CN)) δ (ppm): 2.23 (s, 2H), 2.27 (s, 3H), 2.46 (s, 6H), 3.78 (s, 6H), 6.78 (s, 2H), 6.98 (s, 2H), 7.39 (s, 2H).

^{19}F NMR (376 MHz, (CD_3CN)) δ (ppm): -79.27 (s).

ESI-MS: m/z(+) [Au(MeIM)$_2$(I)(Mes)]$^+$ 607.04 (calc. 607.063).

The reaction mixture was heated for 4 days at 55 °C, the product of iodomesitylene was identified by ^{1}H NMR.

^{1}H NMR (400 MHz, (CD_3CN)) δ (ppm): 2.21 (s, 3H), 2.39 (s, 6H), 6.94 (s, 2H).

Synthesis of [Au(MeIM)$_2$(Br)(Mes)][OTf]

To a solution of [Au(MeIM)$_2$(MeCN)(Mes)][OTf]$_2$ (15 mg, 0.012 mmol) in CD_3CN, 1 equivalent of [NBu$_4$][Br] (3 mg, 0.012 mmol) was added. The solution turned to a dark brown with small amounts of golden particles forming.

^{1}H NMR (400 MHz, (CD_3CN)) δ (ppm): 2.23 (s, 2H), 2.25 (s, 3H), 2.36 (s, 6H), 3.83 (s, 6H), 6.90 (s, 2H), 7.15 (s, 2H), 7.19 (s, 2H), 7.90 (s, 2H),

^{19}F NMR (376 MHz, (CD_3CN)) δ (ppm): -79.3 (s).

ESI-MS: m/z(+) [Au(MeIM)$_2$(Br)(Mes)]$^+$ 559.04(^{35}Br)/561.02(^{37}Br) (calc. 559.077)

A single crystal suitable for X-ray diffraction was grown by vapour diffusion at -40 °C with doping the CD_3CN reaction mixture with 1 equivalent (by volume) CD_2Cl_2/n-hexanes.

The reaction mixture was heated for 4 days at 55 °C, the product of bromomesitylene was identified by ^{1}H NMR (quantitative conversion).

^{1}H NMR (400 MHz, (CD_3CN)) δ (ppm): 2.22 (s, 3H), 2.34 (s, 6H), 6.96 (s, 2H).

Synthesis of [Au(MeIM)$_2$(Cl)(Mes)][OTf]

To a solution of [Au(MeIM)$_2$(MeCN)(Mes)][OTf]$_2$ (22 mg, 0.026 mmol) in CD_3CN, 1 equivalent of [NBu$_4$][Cl] (7 mg, 0.026 mmol) was added. The solution turned to a darker brown immediately after shaking.

^{1}H NMR (400 MHz, (CD_3CN)) δ (ppm): 2.23 (s, 2H), 2.27 (s, 3H), 2.38 (s, 6H), 3.73 (s, 6H), 6.79 (s, 2H), 6.94 (s, 2H), 7.20 (s, 2H).

^{19}F NMR (376 MHz, (CD_3CN)) δ (ppm): -79.15 (s).

ESI-MS: m/z(+) $[Au(MeIM)_2(Cl)(Mes)]^+$ 515.06(^{19}Cl)/517.04(^{19}Cl) (calc. 515.128).

The reaction mixture was heated for 4 days at 55 °C, the product of chloromesitylene was identified by ^1H NMR (quantitative conversion).

^1H NMR (400 MHz, (CD_3CN)) δ (ppm): 2.24 (s, 3H), 2.30 (s, 6H), 6.95 (s, 2H).

Reaction of $[Au(MeIM)_2(NTf_2)_2][OTf]$ + Mes

To a sample of $[Au(MeIM)_2(NTf_2)_2][OTf]$ (10 mg, 0.010 mmol) in CD_2Cl_2, 1 equivalent of mesitylene (1 mg, 0.01 mol) was added to the Au solution dropwise. The reaction immediately proceeded, and the solution turned black. The products of $[Au(MeIM)_2]^+$ and Mes-Mes were observed by ^1H NMR:

^1H NMR (400 MHz, (CD_2Cl_2)) δ (ppm): $[Au(MeIM)_2]^+$ 3.38 (s, 3H), 7.15 (s, 1H), 7.18 (s, 1H), 7.90 (s, 1H).

^1H NMR (400 MHz, (CD_2Cl_2)) δ (ppm): Coupled mesitylene, 2.25 (s, 6H), 2.30 (s, 3H), 6.90 (s, 2H).

Reaction of $[Au(MeIM)_2(NTf_2)_2][OTf]$ + Mes + $[NBu_4][I]$

A sample of $[Au(MeIM)_2(NTf_2)_2][OTf]$ (9 mg, 0.0083 mmol) was cooled to near freezing in a cold well by $N_2(l)$ in CD_2Cl_2, 1 equivalent of mesitylene was added (1 mg, 0.0083 mol) was mixed with $[NBu_4][I]$ (3 mg, 0.0083 mol) and added to the Au solution dropwise.

^1H NMR (400 MHz, (CD_2Cl_2)) δ (ppm): $[Au(MeIM)_2]^+$ 3.38 (s, 3H), 7.15 (s, 1H), 7.18 (s, 1H), 7.90 (s, 1H).

The iodomesitylene product was observed by ^1H NMR:

^1H NMR (400 MHz, (CD_2Cl_2)) δ (ppm): iodomesitylene - 2.22 (s, 3H), 2.25 (s, 6H), 6.90 (s, 2H).

Reaction of $[Au(MeIM)_2(NTf_2)_2][OTf]$ + Mes + $[NBu_4][Br]$

A sample of $[Au(MeIM)_2(NTf_2)_2][OTf]$ (8 mg, 0.0074 mmol) was cooled to near freezing in a cold well by $N_2(l)$ in CD_2Cl_2, 1 equivalent of mesitylene was added (1 mg, 0.0074 mol) was mixed with $[NBu_4][Br]$ (2 mg, 0.0074 mol) and added to the Au solution dropwise.

^1H NMR (400 MHz, (CD_2Cl_2)) δ (ppm): $[Au(MeIM)_2]$+ 3.38 (s, 3H), 7.15 (s, 1H), 7.18 (s, 1H), 7.90 (s, 1H).

The bromomesitylene product was identified by ^{1}H NMR:

^{1}H NMR (400 MHz, (CD_2Cl_2)) δ (ppm): bromomesitylene 2.22 (s, 3H), 2.35 (s, 6H), 6.90 (s, 2H).

Reaction of [Au(MeIM)$_2$(NTf$_2$)$_2$][OTf] + Mes + [NBu$_4$][Cl]

A sample of [Au(MeIM)$_2$(NTf$_2$)$_2$][OTf] (8 mg, 0.0074 mmol) was cooled to near freezing in a cold well by N_2(l) in CD_2Cl_2, 1 equivalent of mesitylene (1 mg, 0.0074 mmol) was mixed with [NBu$_4$][Cl] (2 mg, 0.014 mmol) and added to the Au solution dropwise.

^{1}H NMR (400 MHz, (CD_2Cl_2)) δ (ppm): [Au(MeIM)$_2$]$^+$ 3.38 (s, 3H), 7.15 (s, 1H), 7.18 (s, 1H), 7.90 (s, 1H).

The chloromesitylene product was observed by ^{1}H NMR:

^{1}H NMR (400 MHz, (CD_2Cl_2)) δ (ppm): chloromesitylene - 2.30 (s, 6H), 2.31 (s, 3H), 7.00 (s, 2H).

Reaction of [Au(MeIM)$_2$($C_6H_4(CH_3)$)(OTf)][NTf$_2$] + [NBu$_4$][I]

A sample of [Au(MeIM)$_2$($C_6H_4(CH_3)$)(OTf)][NTf$_2$] generated *in-situ* from [Au(MeIM)$_2$F$_2$][OTf] (9 mg, 0.016 mmol) in $CDCl_3$ with 2 equivalents of TMS-NTf$_2$ (11 mg, 0.032 mmol) and 1 equivalent of toluene (1 mg, 0.016 mmol), had 1 equivalent of [NBu$_4$][I] added (6 mg, 0.016 mol) turning the reaction to orange. [Au(MeIM)$_2$(I)($C_6H_4(CH_3)$)]$^+$ was identified by ^{1}H NMR.

^{1}H NMR (400 MHz, (CD_2Cl_2)) δ (ppm): 2.38 (s, 3H), 3.93 (s, 6H), 6.91 (d, 2H), 7.00 (d, 2H), 7.22 (s, 2H), 7.39 (s, 2H), 8.45 (s, 2H).

Trace amounts of iodotoluene were observed in ^{1}H NMR instantly.

The reaction was repeated in a $CDCl_3$, which lead to incomplete metalation of the aryl species, but allowed for heating to 55 °C for overnight, with iodotoluene was identified as the major product by ^{1}H NMR (quantitative conversion).

^{1}H NMR (400 MHz, (CD_2Cl_2)) δ (ppm): 2.28 (s, 3H), 6.94 (d, 2H), 7.56 (d, 2H).

Reaction of [Au(MeIM)$_2$($C_6H_4(CH_3)$)(OTf)][NTf$_2$] + [NBu$_4$][Br]

A sample of [Au(MeIM)$_2$($C_6H_4(CH_3)$)(OTf)][NTf$_2$] generated *in-situ* from [Au(MeIM)$_2$F$_2$][OTf] (9 mg, 0.016 mmol) in $CDCl_3$ with 2 equivalents TMS-NTf$_2$ (11 mg, 0.032 mmol) and 1 equivalent toluene (1, 0.016 mmol), had 1 equivalent of [NBu$_4$][Br] added (5 mg, 0.016

mol) turning the reaction an orange colour. $[Au(MeIM)_2(Br)(C_6H_4(CH_3))]^+$ was identified by ^1H NMR, NMR conversion of 50 % in $CDCl_3$.

^1H NMR (400 MHz, ($CDCl_3$)) δ (ppm): 2.33 (s, 3H), 3.80 (s, 3H), 3.93 (s, 3H). 6.94 (d, 2H), 6.95 (s, 1H), 7.00 (d, 2H), 7.14 (s, 1H) 7.76 (s, 1H), 8.32 (s, 1H), 8.44 (s, 1H).

After heating to 55 °C for 6 days, bromotoluene was identified by ^1H NMR (81 % conversion).

^1H NMR (400 MHz, (CD_2Cl_2)) δ (ppm): 2.29 (s, 3H), 7.04 (d, 2H), 7.35 (d, 2H).

Reaction of $[Au(MeIM)_2(C_6H_4(CH_3))(OTf)][NTf_2]$ + $[NBu_4][Cl]$

A sample of $[Au(MeIM)_2(C_6H_4(CH_3))(OTf)][NTf_2]$ generated *in-situ* from $[Au(MeIM)_2F_2][OTf]$ (9 mg, 0.016 mmol) in $CDCl_3$ with 2 equivalents TMS-NTf_2 (11 mg, 0.032 mmol) and 1 equivalent toluene (1, 0.016 mmol), had 1 equivalent of $[NBu_4][Cl]$ added (4 mg, 0.016 mmol) turning the reaction a pale yellow colour.

$[Au(MeIM)_2(Cl)(C_6H_4(CH_3))]^+$ was identified by ^1H NMR.

^1H NMR (400 MHz, (CD_2Cl_2)) δ (ppm): 3.32 (s, 3H), 3.94 (s, 6H), 6.95 (d, 2H), 7.02 (d, 2H), 7.24 (s 2H), 7.42 (s, 2H), 8.51 (s, 2H).

The reaction was repeated in a $CDCl_3$, which lead to incomplete metalation of the aryl species, but allowed for heating to 55 °C for 6 days, no chlorotoluene was observed.

Reaction of $[Au(MeIM)_2(C_6H_5)(OTf)][NTf]$ + $[NBu_4]I$

A sample of $[Au(MeIM)_2(C_6H_5)(OTf)][NTf_2]$ generated *in-situ* from $[Au(MeIM)_2F_2][OTf]$ (9 mg, 0.016 mmol) in $CDCl_3$ with 2 equivalents TMS-NTf_2 (11 mg, 0.032 mmol) and 1 equivalent C_6H_6 (1, 0.016 mmol), had 1 equivalent of $[NBu_4][I]$ added (6 mg, 0.016 mmol) turning the reaction orange.

$[Au(MeIM)_2(I)(C_6H_5)]^+$ was identified by ^1H NMR.

^1H NMR (400 MHz, ($CDCl_3$)) δ (ppm): 3.92 (s 6H), 6.99 (d, 1H), 7.12 (t, 2H), 7.13 (t, 2H), 7.19 (t, 2H), 7.20 (s, 2H), 7.35 (s, 2H), 8.48 (s, 2H).

The reaction was repeated in CH_2Cl_2, and diluted 3-fold in $CDCl_3$, which allowed for heating to 55 °C for 6 days producing C_6H_5I identified by ^1H NMR, quantitatively with respect to $[Au(MeIM)_2(I)(C_6H_5)]^+$.

^1H NMR (400 MHz, (CD_2Cl_2)) δ (ppm): 7.10 (t, 2H), 7.32 (t, 2H), 7.70 (d, 2H).

Reaction of $[Au(MeIM)_2(C_6H_5)(OTf)][NTf_2]$ + $[NBu_4][Br]$

A sample of $[Au(MeIM)_2(C_6H_5)(OTf)][NTf_2]$ generated *in-situ* from $[Au(MeIM)_2F_2][OTf]$ (8 mg, 0.014 mol) in $CDCl_3$ with 2 equivalents TMS-NTf_2 (11 mg, 0.028 mmol) and 1 equivalent C_6H_6 (1 mg, 0.014 mmol), had 1 equivalent of $[NBu_4][Br]$ added (4 mg, 0.014 mmol) turning the reaction a pale orange colour.

$[Au(MeIM)_2(Br)(C_6H_5)]^+$ was identified by ^1H NMR.

^{1}H NMR (400 MHz, ($CDCl_3$)) δ (ppm): 3.85 (s 3H), 3.99 (s, 3H), 7.15 (s, 1H), 7.22 (m, 2.5H), 7.45 (s, 1H), 8.25 (s, 1H), 8.53 (s, 1H)

For further clarity on the product of the reaction it was repeated in a $CDCl_3$, which lead to incomplete metalation of the aryl species, but allowed for heating to 55 °C for 6 days producing bromobenzene quantitatively relative to the amount of $[Au(MeIM)_2(Br)(C_6H_5)]^+$, identified by ^1H NMR

^{1}H NMR (400 MHz, ($CDCl_3$)) δ (ppm): 7.70 (t, 2H), 7.32 (t, 1.5H), 7.70 (d, 2H)

m-H integration is slightly out due to peak overlap

Reaction of $[Au(MeIM)_2(C_6H_5)(OTf)][NTf_2]$ + $[NBu_4][Cl]$

A sample of $[Au(MeIM)_2(C_6H_5)(OTf)][NTf_2]$ generated *in-situ* from $[Au(MeIM)_2F_2][OTf]$ (8 mg, 0.014 mol) in $CDCl_3$ with 2 equivalents of TMS-NTf_2 (11 mg, 0.028 mmol) and 1 equivalent of C_6H_6 (1 mg, 0.014 mol), had 1 equivalent of $[NBu_4][Cl]$ added (4 mg, 0.014 mmol) turning the reaction yellow.

$[Au(MeIM)_2(Cl)(C_6H_5)]^+$ was identified by ^1H NMR.

^{1}H NMR (400 MHz, ($CDCl_3$)) δ (ppm): 3.74 (s, 3H), 3.88 (s, 3H), 6.89 (s, 1H), 7.01 (s, 1H),7.10 (m, 1H) 7.19 (m, 3H) 7.25 (m, 1H), 7.35 (s, 1H) 7.44 (s, 1H), 8.11 (s, 1H), 8.45 (s, 1H)

The reaction was repeated in a $CDCl_3$, which lead to incomplete metalation of the aryl species, but allowed for heating to 55°C for 3 days with no observable change.

X-Ray Crystallographic Data

Crystals presented in this work rapidly decompose and need to be kept at 0°C or below. Transport from glovebox to XRD was performed by isolating a small sample of crystals into a sealed N_2 filled container to be embedded in a slurry of ice in EtOH and mounted immediately on arrival. Single crystals were selected under n-paratone oil, mounted on a nylon loop, and held under a stream of N_2 (150 K) on a Rigaku SuperNova CCD diffractometer using Cu*K*α radiation. Structures were solved using SHELXT[5] (intrinsic phasing) and refined with the SHELXL[6] program. Non-hydrogen atoms were refined with anisotropic displacement parameters. Hydrogen atoms were placed in geometrically estimated positions and refined using the riding model. Coordinates and anisotropic thermal parameters of all non-hydrogen atoms were refined. All calculations were carried out using the program Olex2.[7]

All structures except those containing the $[Au(III)(MeIM_2)(C_6H_4(CH_3)(OTf)]^+$ provided good refinement data. Analysis of the X-ray diffraction data collected on crystals containing $[Au(III)(MeIm)_2(C_6H_4(CH_3)(OTf)]^+$ cations revealed the cations (2 per ASU) to be very well resolved and undoubtedly present in the crystal. Analysis of the other components of the crystal indicates the presence of disorder and decomposition products, decomposition is supported by NMR analysis, which proved challenging to model. The best model produced could be refined reasonably well with a weighted residual factor (*wR*2- all data) = 0.1872.

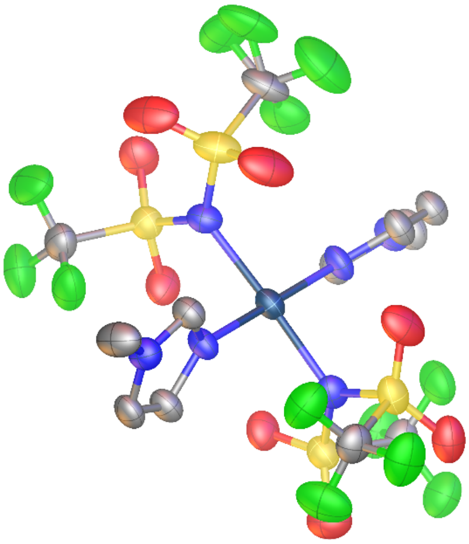

Figure S1: Thermal ellipsoid plot of [Au(MeIM)$_2$(NTf$_2$)$_2$][NTf$_2$], thermal ellipsoids are drawn at the 50% probability level. Hydrogen atoms NTf$_2^-$ counterion removed for clarity

Table S1 Crystallographic information of [Au(MeIM)$_2$(NTf$_2$)$_2$][NTf$_2$]

Empirical Formula	$C_{14}H_{12}N_7O_{12}F_{18}S_6Au$
FW (g/mol)	1201.63
Temperature/K	150.0(1)
Crystal system	triclinic
Space group	$P\bar{1}$
a/Å	10.8110(3)
b/Å	12.8546(4)
c/Å	14.9788(3)
α/°	69.117(2)
β/°	87.493(2)
γ/°	65.786(3)
Volume/Å^3	1760.91(9)
Z	2
ρ_{calc}g/cm^3	2.266
μ/mm^{-1}	12.699
F(000)	1156.0
Crystal size/mm^3	0.08 × 0.04 × 0.03
2Θ range for data collection/°	8.122 to 138.4
Index ranges	$-12 \leq h \leq 13$, $-15 \leq k \leq 15$, $-17 \leq l \leq 18$
Reflections collected	31888
Independent reflections	6441 [R_{int} = 0.1669, R_{sigma} = 0.0842]
Data/restraints/parameters	6441/0/535
Goodness-of-fit on F^2	1.097
Final R indexes [I>=2σ (I)]	R_1 = 0.0603, wR_2 = 0.1580
Final R indexes [all data]	R_1 = 0.0695, wR_2 = 0.1688
Largest diff. peak/hole / e Å^{-3}	2.85/-1.41

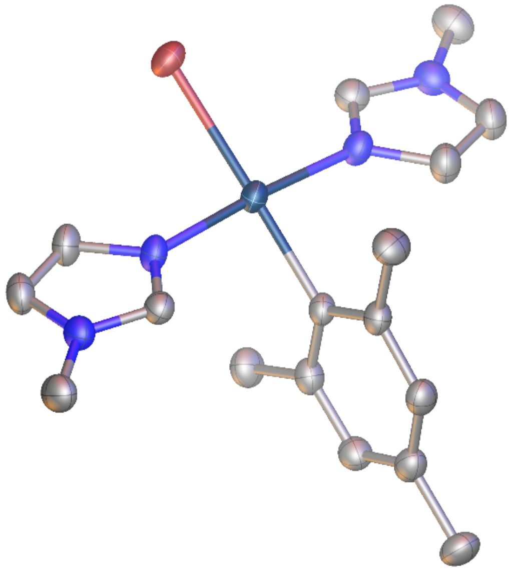

Figure S2 Thermal ellipsoid plot of [Au(MeIM)$_2$(Br)(Mes)][OTf], thermal ellipsoids are drawn at the 50% probability level. Hydrogen atoms and OTf$^-$ counterion removed for clarity

Table S2 Crystallographic information of of [Au(MeIM)$_2$(Br)(Mes)][OTf]

Empirical Formula	$C_{18}H_{23}AuBrN_4S_1F_3O_3$
Formula weight	709.34
Temperature/K	150.0(1)
Crystal system	monoclinic
Space group	$P2_1/c$
a/Å	11.4363(1)
b/Å	13.7318(1)
c/Å	14.7621(1)
α/°	90
β/°	96.990(1)
γ/°	90
Volume/Å^3	2301.02(3)
Z	4
ρ_{calc}g/cm^3	2.048
μ/mm^{-1}	15.367
F(000)	1360.0
Crystal size/mm^3	0.21 × 0.13 × 0.07
2Θ range for data collection/°	7.788 to 142.552
Index ranges	$-13 \le h \le 14$, $-16 \le k \le 16$, $-18 \le l \le 16$
Reflections collected	45091
Independent reflections	4460 [R_{int} = 0.0753, R_{sigma} = 0.0313]
Data/restraints/parameters	4460/0/285
Goodness-of-fit on F^2	1.070
Final R indexes [I>=2σ (I)]	R_1 = 0.0281, wR_2 = 0.0765
Final R indexes [all data]	R_1 = 0.0292, wR_2 = 0.0774
Largest diff. peak/hole / e Å^{-3}	1.35/-1.94

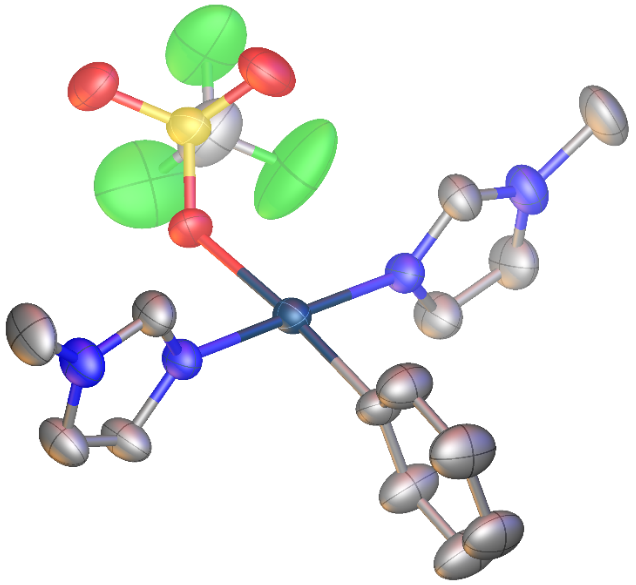

Figure S3 Thermal ellipsoid plot of [Au(MeIM)$_2$(C$_6$H$_5$)(OTf)][NTf$_2$], thermal ellipsoids are drawn at the 50% probability level. Hydrogen atoms, NTf$_2^-$ counterion and CH$_2$Cl$_2$ solvate removed for clarity

Table S3 Crystallographic information of [Au(MeIM)$_2$(C$_6$H$_5$)(OTf)][NTf$_2$]

Empirical formula	$C_{21}H_{23}AuCl_2D_2F_9N_5O_7S_3$
Formula weight	996.52
Temperature/K	190.0(1)
Crystal system	monoclinic
Space group	*C*2/*c*
a/Å	21.4452(1)
b/Å	9.9280(1)
c/Å	31.6635(2)
α/°	90
β/°	93.450(1)
γ/°	90
Volume/Å^3	6729.19(9)
Z	8
ρ_{calc}g/cm^3	1.967
μ/mm^{-1}	12.325
F(000)	3872.0
Crystal size/mm^3	0.37 × 0.05 × 0.03
2Θ range for data collection/°	8.26 to 142.538
Index ranges	$-26 \le h \le 26$, $-12 \le k \le 12$, $-37 \le l \le 38$
Reflections collected	67139
Independent reflections	6533 [R_{int} = 0.0699, R_{sigma} = 0.0248]
Data/restraints/parameters	6533/0/381
Goodness-of-fit on F^2	1.069
Final *R* indexes [I>=2σ (I)]	R_1 = 0.0331, wR_2 = 0.0847
Final *R* indexes [all data]	R_1 = 0.0345, wR_2 = 0.0856
Largest diff. peak/hole / e Å^{-3}	2.45/-0.83

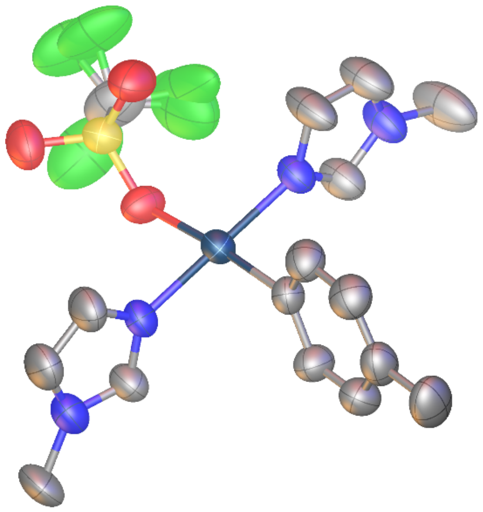

Figure S4 Thermal ellipsoid plot of the Au(III) cation in 2[Au(III)(MeIM)$_2$(C$_6$H$_4$(CH$_3$))(OTf)][Au(I)(NTf$_2$)$_2$][NTf$_2$] (H$_2$O)$_2$, thermal ellipsoids are drawn at the 50% probability level. Hydrogen and NTf$_2^-$ counterion removed for clarity

Table S4 Crystallographic information of 2[Au(III)(MeIM)$_2$(C$_6$H$_4$(CH$_3$))(OTf)][Au(I)(NTf$_2$)$_2$][[NTf$_2$] (H$_2$O)$_2$

Empirical formula	$C_{37}H_{38}Au_{2.5}F_{21}N_{10.5}O_{19}S_7$
Formula weight	2049.61
Temperature/K	190.0(1)
Crystal system	triclinic
Space group	$P\bar{1}$
a/Å	9.1167(1)
b/Å	14.1734(2)
c/Å	25.0756(3)
α/°	95.4082(9)
β/°	92.3072(9)
γ/°	94.5118(9)
Volume/Å^3	3212.00(6)
Z	2
ρ_{calc}g/cm^3	2.119
μ/mm^{-1}	13.913
F(000)	1968.0
Crystal size/mm^3	0.22 × 0.06 × 0.02
2Θ range for data collection/°	6.908 to 142.696
Index ranges	$-11 \le h \le 11$, $-17 \le k \le 17$, $-30 \le l \le 19$
Reflections collected	63653
Independent reflections	12422 [R_{int} = 0.0644, R_{sigma} = 0.0408]
Data/restraints/parameters	12422/2/924
Goodness-of-fit on F^2	1.035
Final R indexes [I>=2σ (I)]	R_1 = 0.0624, wR_2 = 0.1801
Final R indexes [all data]	R_1 = 0.0696, wR_2 = 0.1872
Largest diff. peak/hole / e Å^{-3}	1.51/-3.56

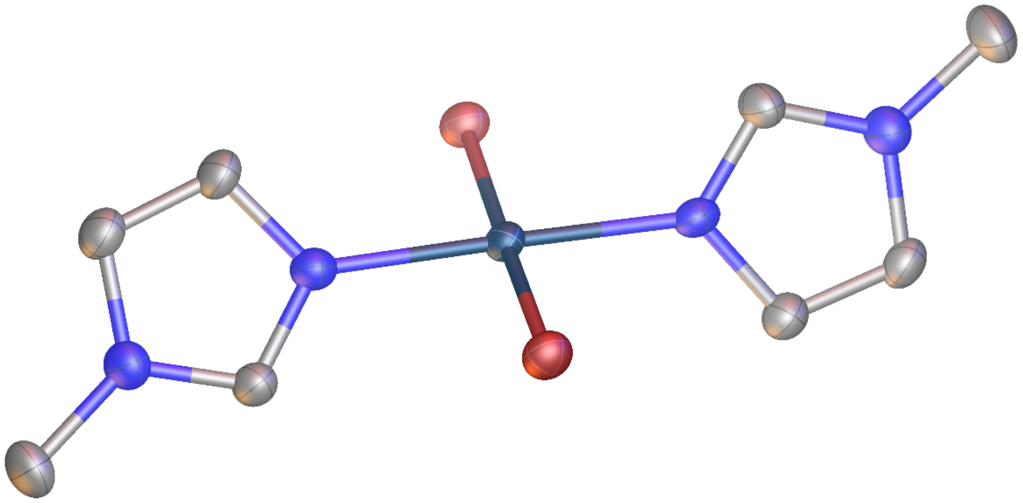

Figure S5 Thermal ellipsoid plot of [Au(MeIM)$_2$Br$_2$][OTf], thermal ellipsoids are drawn at the 50% probability level. Hydrogen atoms, OTf$^-$ counterion and MeCN solvate removed for clarity

Table S5 Crystallographic information of [Au(MeIM)$_2$Br$_2$][OTf]

Empirical formula	$C_{11}H_{15}AuBr_2F_3N_5O_3S$
Formula weight	711.13
Temperature/K	150.0(1)
Crystal system	triclinic
Space group	$P\bar{1}$
a/Å	7.5453(4)
b/Å	8.2539(7)
c/Å	15.894(1)
α/°	78.519(6)
β/°	86.132(5)
γ/°	80.391(6)
Volume/Å^3	955.86(12)
Z	2
ρ_{calc}g/cm^3	2.471
μ/mm^{-1}	20.869
F(000)	664.0
Crystal size/mm^3	0.28 × 0.11 × 0.04
2Θ range for data collection/°	11.076 to 139.612
Index ranges	$-9 \le h \le 9$, $-9 \le k \le 9$, $-19 \le l \le 18$
Reflections collected	7954
Independent reflections	3561 [R_{int} = 0.0247, R_{sigma} = 0.0245]
Data/restraints/parameters	3561/0/256
Goodness-of-fit on F^2	1.080
Final R indexes [I>=2σ (I)]	R_1 = 0.0353, wR_2 = 0.0943
Final R indexes [all data]	R_1 = 0.0361, wR_2 = 0.0951

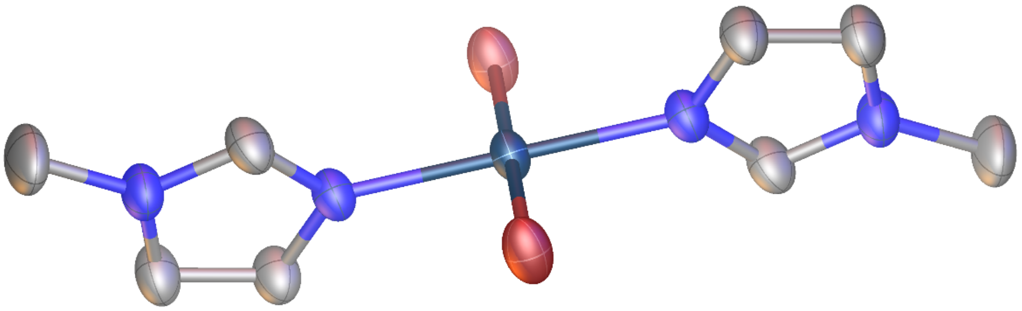

Figure S6 Thermal ellipsoid plot of [Au(MeIM)$_2$Br$_2$][NTf$_2$], thermal ellipsoids are drawn at the 50% probability level. Hydrogen atoms and NTf$_2^-$ counterion removed for clarity

Table S6 Crystallographic information of [Au(MeIM)$_2$Br$_2$][NTf$_2$]

Empirical formula	$C_{10}H_{12}AuBr_2F_6N_5O_4S_2$
Formula weight	801.15
Temperature/K	190.0(1)
Crystal system	triclinic
Space group	$P\bar{1}$
a/Å	8.7502(2)
b/Å	9.9640(2)
c/Å	13.8940(2)
α/°	109.098(1)
β/°	92.888(1)
γ/°	104.290(2)
Volume/Å^3	1097.85(4)
Z	2
ρcalcg/cm^3	2.424
μ/mm^{-1}	19.386
F(000)	748.0
Crystal size/mm^3	0.17 × 0.08 × 0.05
2Θ range for data collection/°	6.802 to 142.646
Index ranges	$-10 \le h \le 10$, $-12 \le k \le 12$, $-12 \le l \le 17$
Reflections collected	22701
Independent reflections	4234 [R_{int} = 0.0486, R_{sigma} = 0.0306]
Data/restraints/parameters	4234/0/276
Goodness-of-fit on F^2	1.079
Final R indexes [I>=2σ (I)]	R_1 = 0.0303, wR_2 = 0.0818
Final R indexes [all data]	R_1 = 0.0337, wR_2 = 0.0840
Largest diff. peak/hole / e Å^{-3}	1.92/-1.44

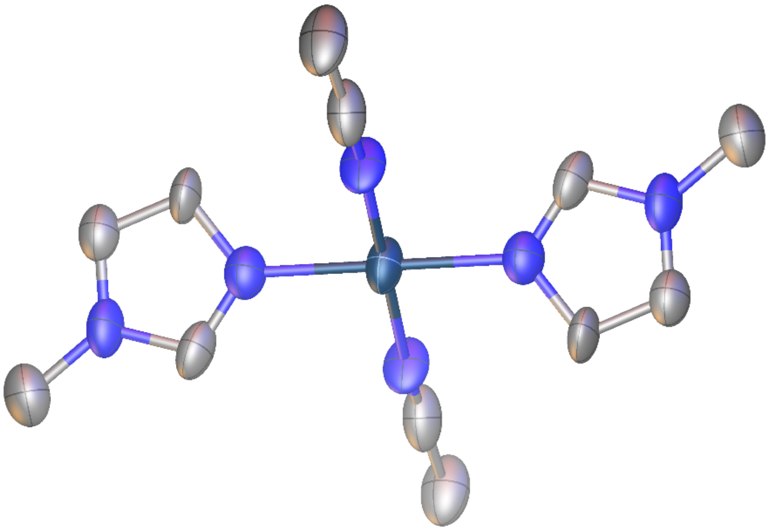

Figure S7 Thermal ellipsoid plot of $[Au(MeIM)_2(MeCN)_2][OTf]_3$ CH_2Cl_2, thermal ellipsoids are drawn at the 50% probability level. Hydrogen atoms, OTf^- counter ions and dichloromethane solvate omitted for clarity.

Table S7 Crystallographic information of $[Au(MeIM)_2(MeCN)_2][OTf]_3$

Empirical formula	$C_{16}H_{20}AuCl_2F_9N_6O_9S_3$
Formula weight	975.43
Temperature/K	150.0(1)
Crystal system	triclinic
Space group	$P\bar{1}$
a/Å	9.9743(4)
b/Å	13.208(1)
c/Å	13.2307(7)
α/°	83.312(5)
β/°	69.326(4)
γ/°	79.175(5)
Volume/Å^3	1599.3(2)
Z	2
ρcalcg/cm^3	2.026
μ/mm^{-1}	13.005
F(000)	944.0
Crystal size/mm^3	0.1 × 0.06 × 0.04
2Θ range for data collection/°	6.824 to 143.602
Index ranges	$-12 \leq h \leq 10$, $-16 \leq k \leq 16$, $-16 \leq l \leq 16$
Reflections collected	31592
Independent reflections	6200 [R_{int} = 0.1271, R_{sigma} = 0.0743]
Data/restraints/parameters	6200/0/478
Goodness-of-fit on F^2	1.080
Final R indexes [I>=2σ (I)]	R_1 = 0.0775, wR_2 = 0.1932
Final R indexes [all data]	R_1 = 0.1082, wR_2 = 0.2125
Largest diff. peak/hole / e Å-3	2.41/-2.35

NMR Spectra

Reaction of $[Au(MeIM)_2F_2][OTf]$ with 2 TMS-NTf_2 in CD_2Cl_2

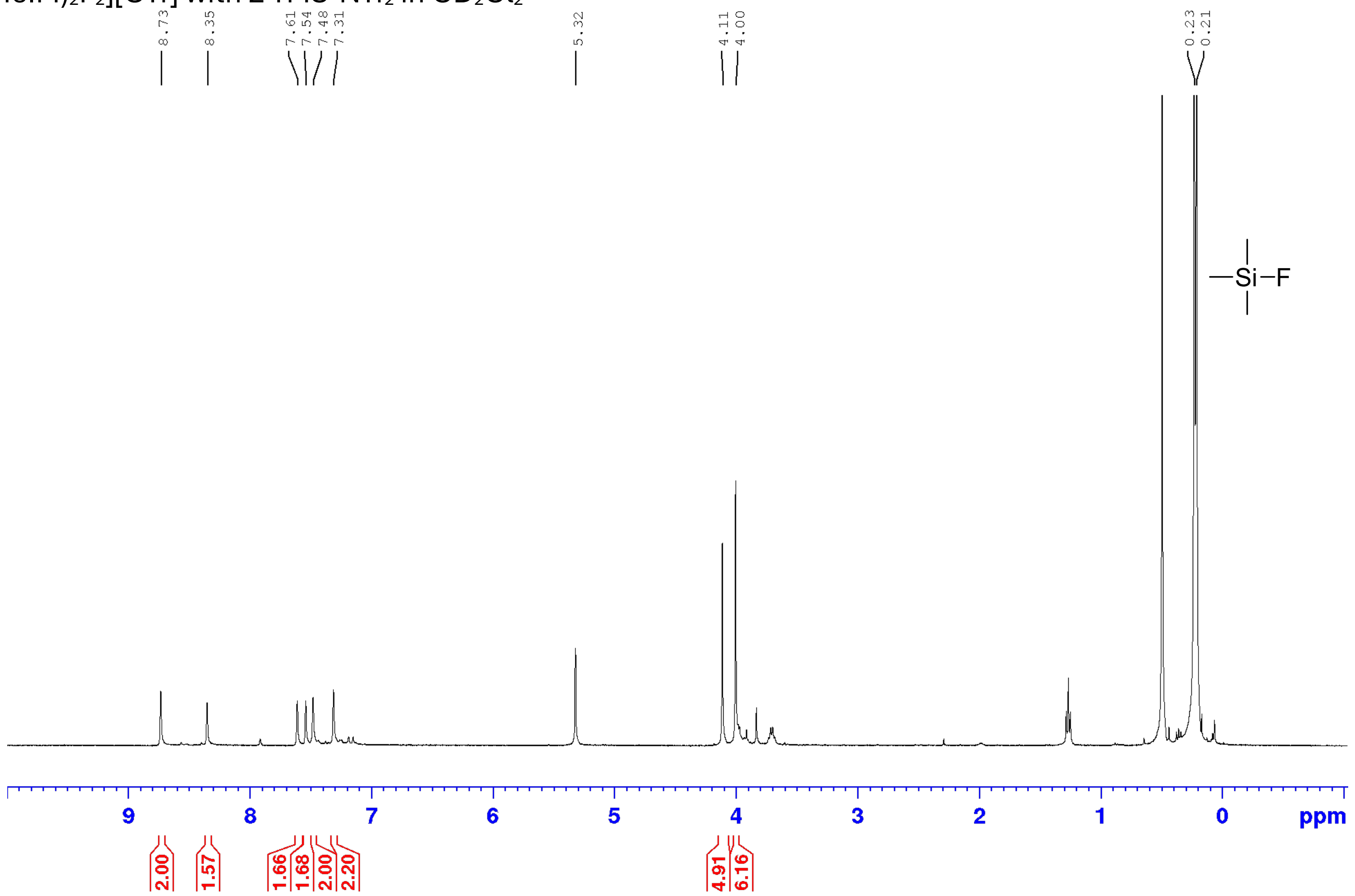

Figure S8 ^{1}H NMR of the reaction of $[Au(MeIM)_2F_2][OTf]$ with 2 TMS-NTf_2 in CD_2Cl_2 resulting in competition between NTf_2 and OTf at the Au centre

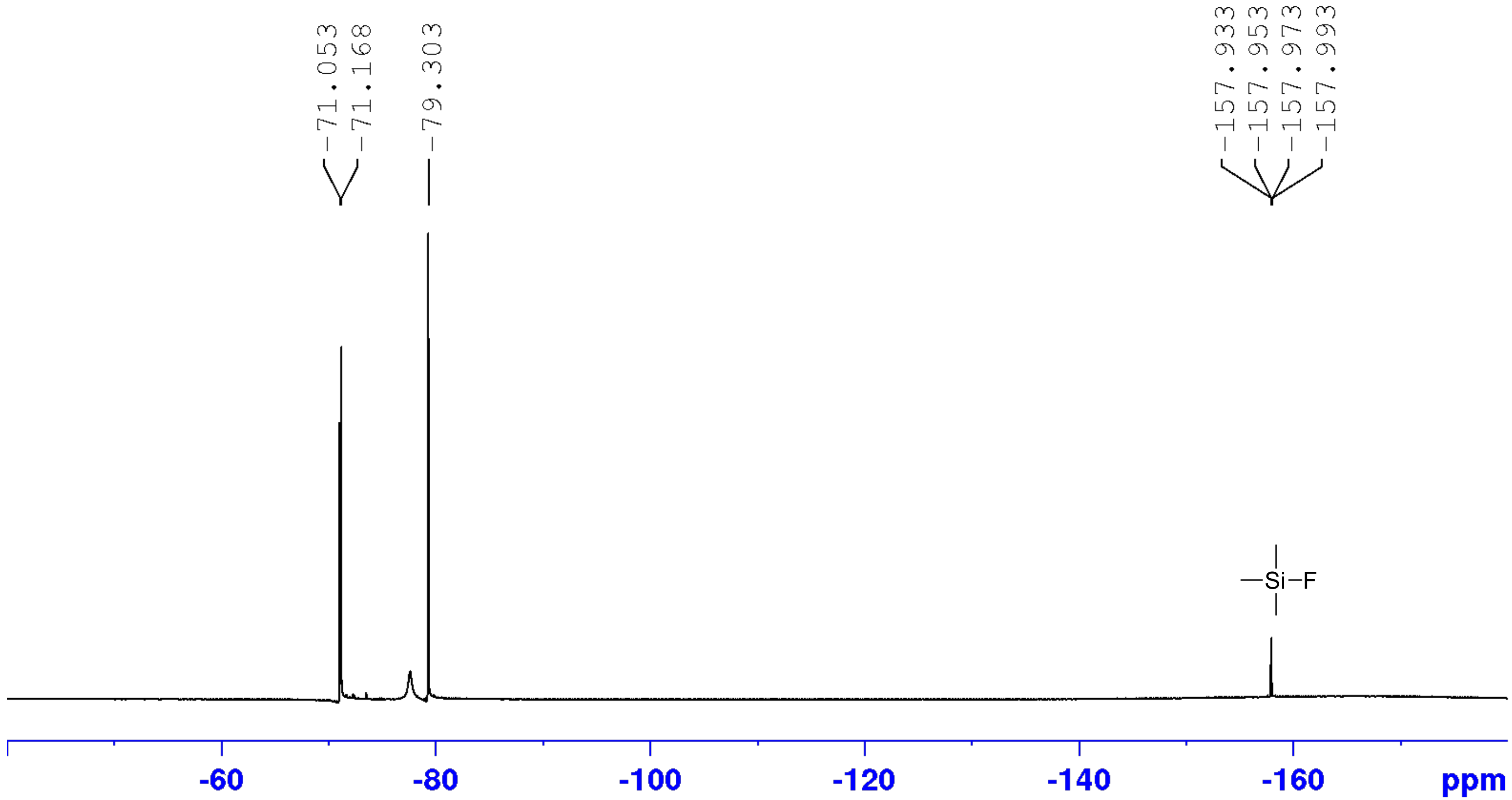

Figure S9 ^{19}F NMR of the reaction of [Au(MeIM)$_2$F$_2$][OTf] with 2 TMS-NTf$_2$ in CD$_2$Cl$_2$ resulting in competition between NTf$_2$ and OTf at the Au centre

[Au(MeIM)$_2$(NTf$_2$)$_2$][NTf$_2$] – CD$_2$Cl$_2$

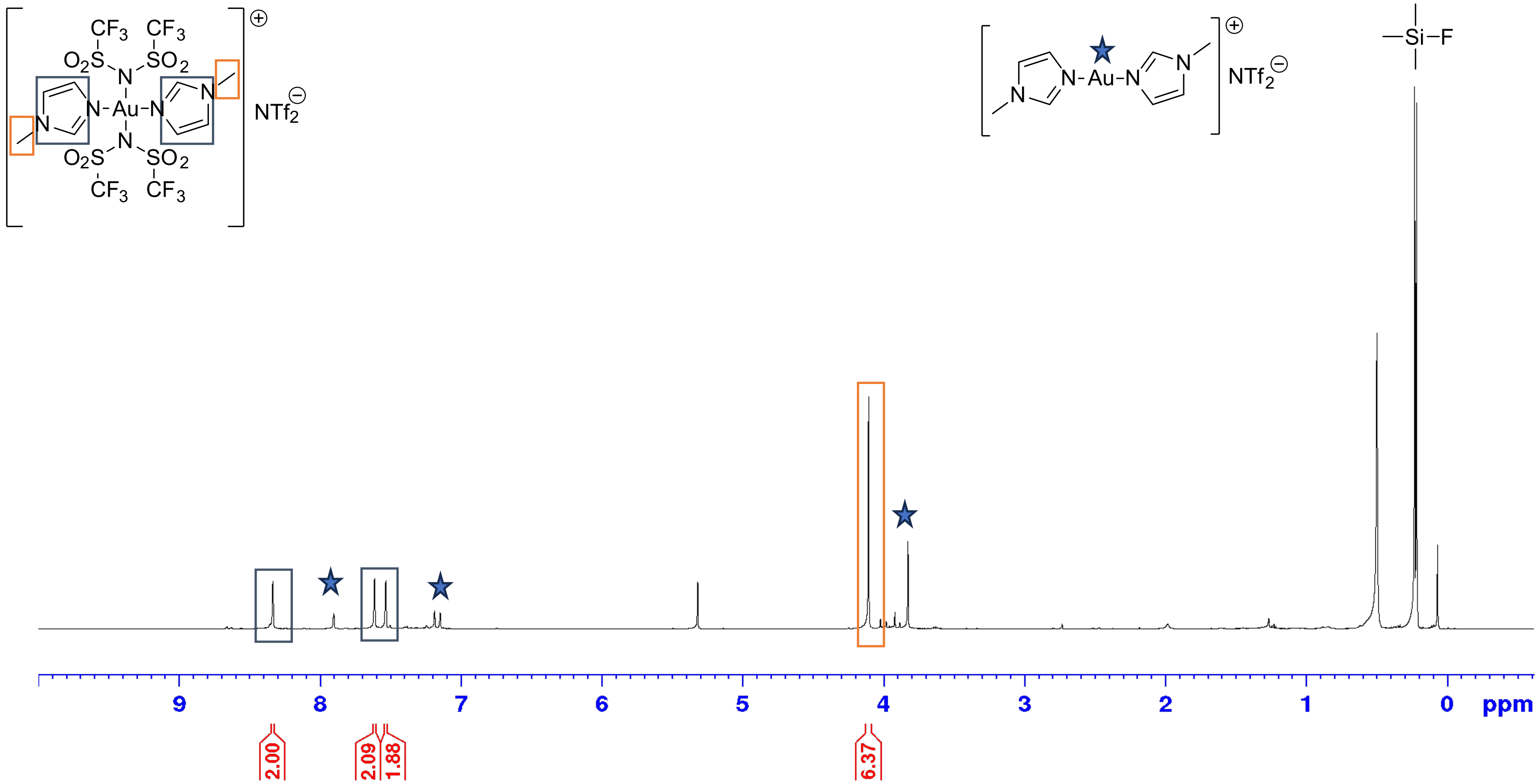

Figure S10 ¹H NMR of [Au(MeIM)$_2$(NTf$_2$)$_2$][NTf$_2$] in CD$_2$Cl$_2$

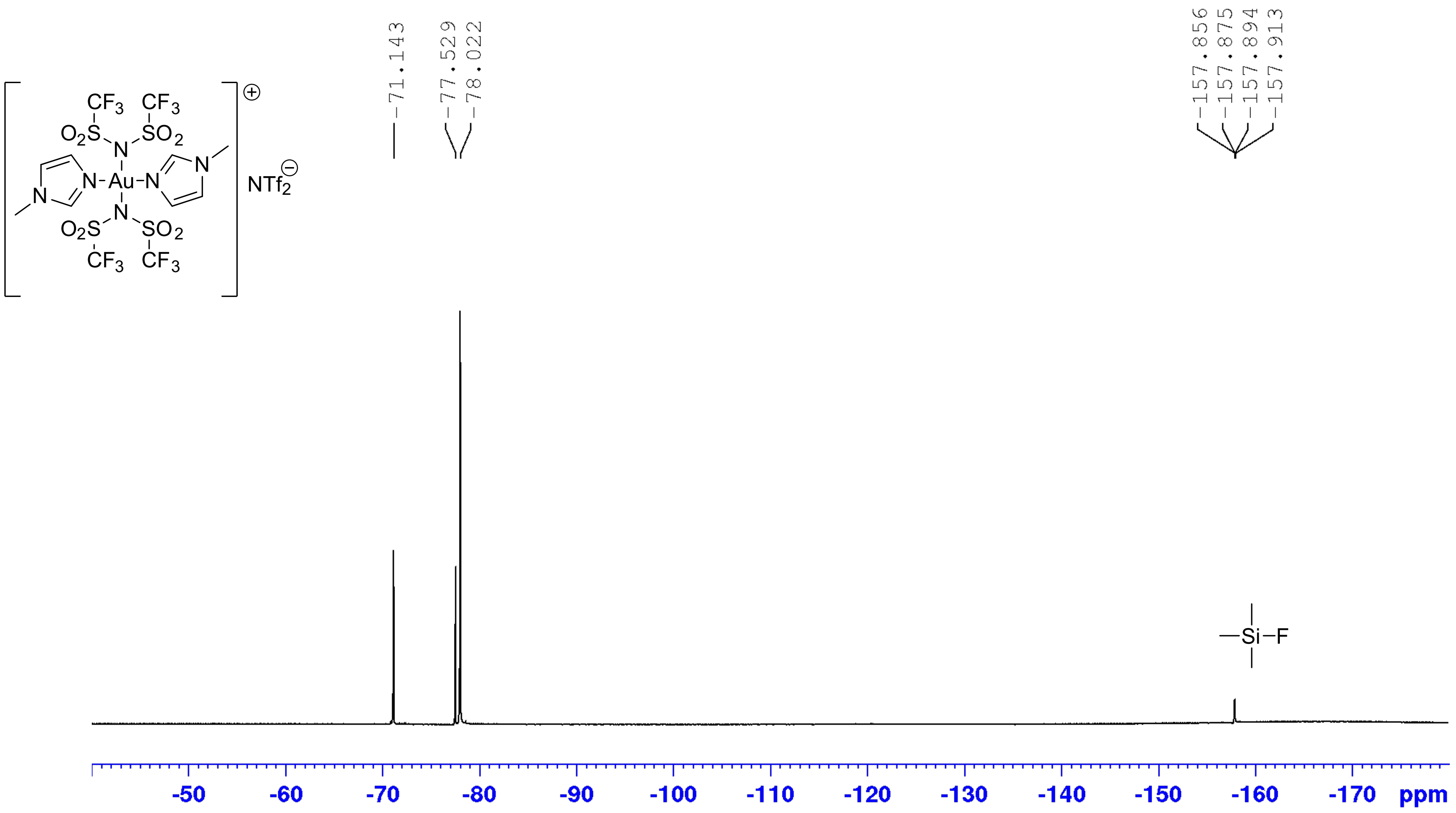

Figure S11 ^{19}F NMR of Au(MeIM)$_2$(NTf$_2$)$_2$][NTf$_2$] in CD$_2$Cl$_2$

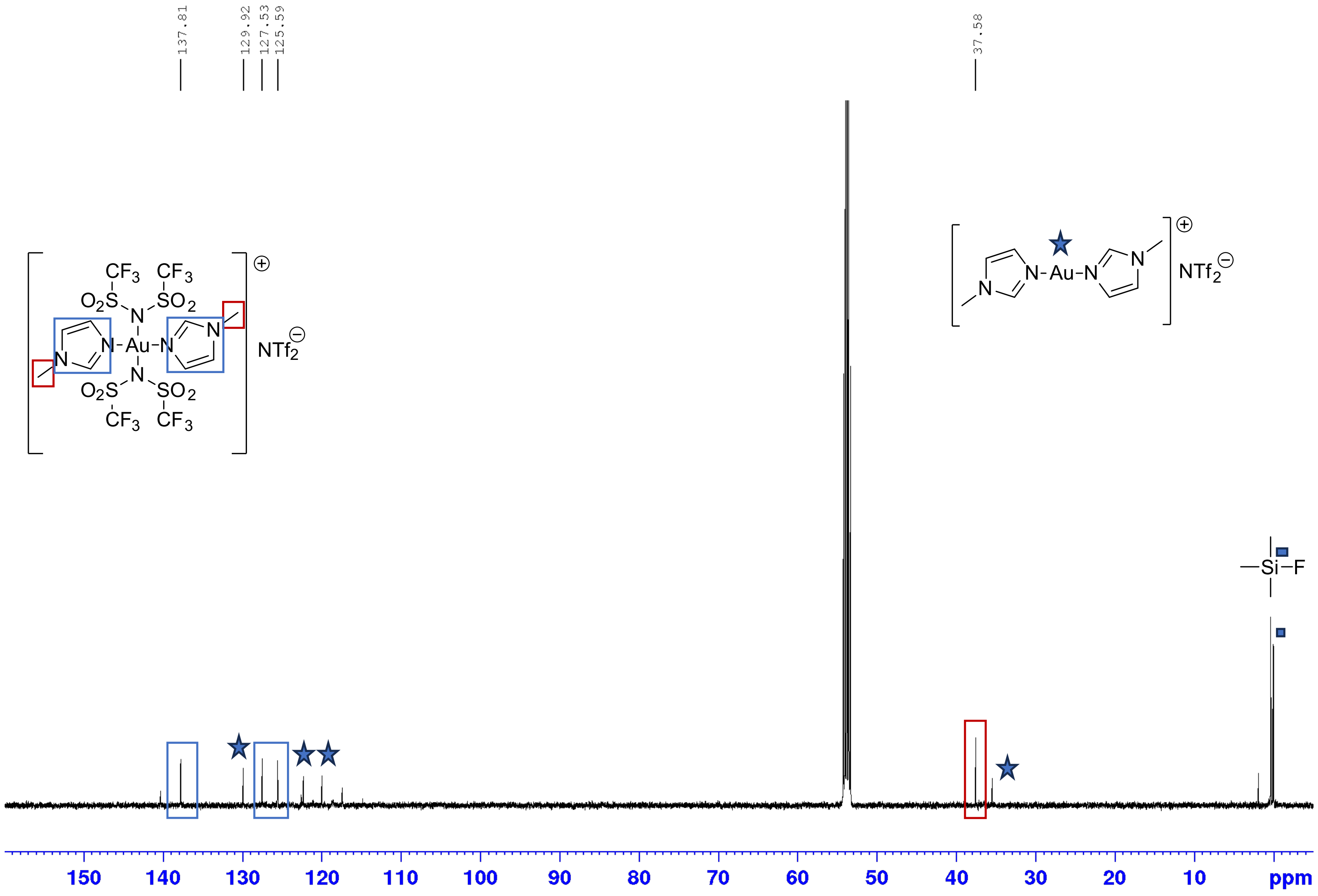

Figure S12 ^{13}C NMR of [Au(MeIM)$_2$(NTf$_2$)$_2$][NTf$_2$] in CD_2Cl_2, starred peaks are Au^{I} bismethylimidazole, *and squares are TMS.*

[Au(MeIM)₂Br₂][OTf] – CD₃CN

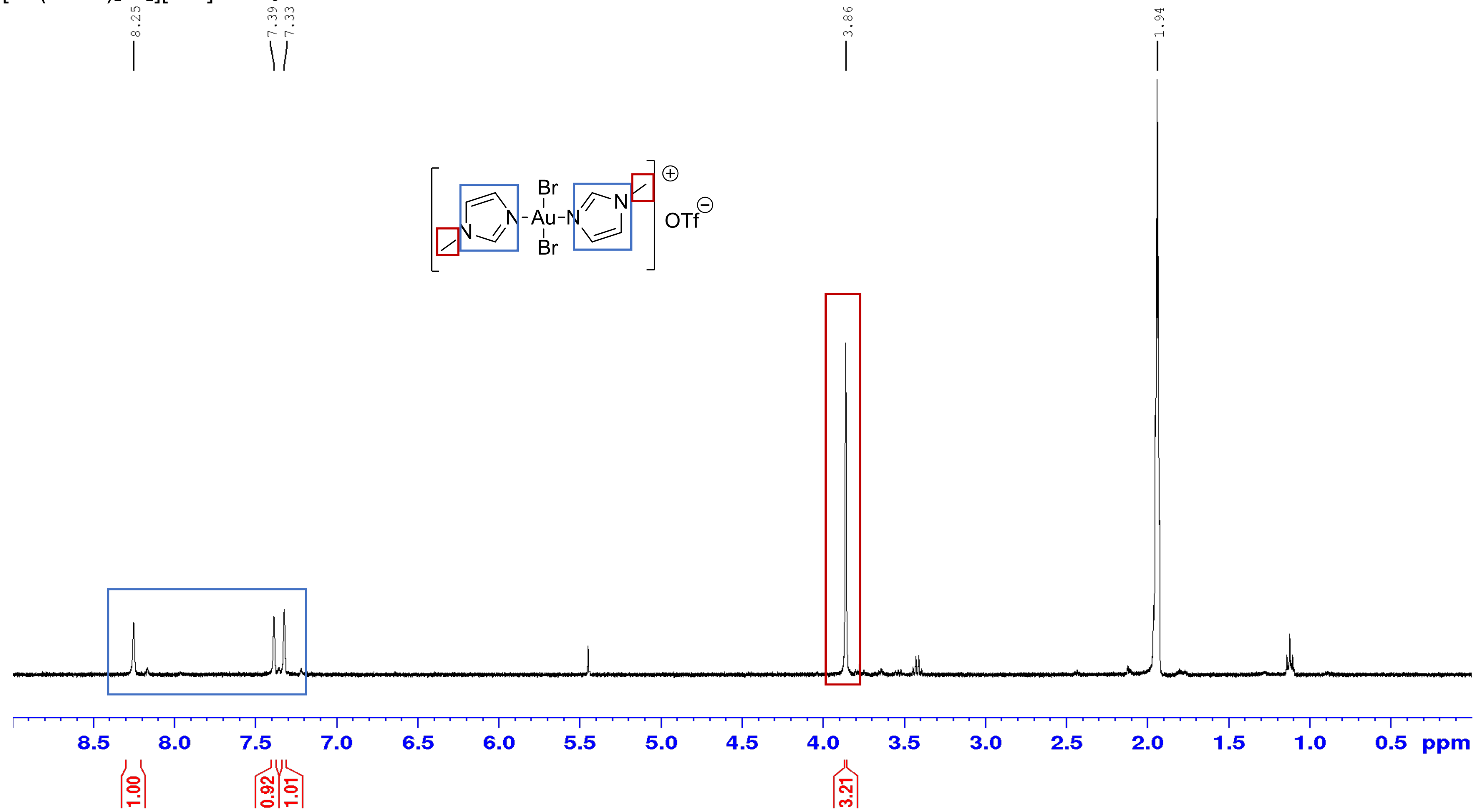

Figure S13 ¹H NMR of [Au(MeIM)₂Br₂][OTf] in CD₃CN

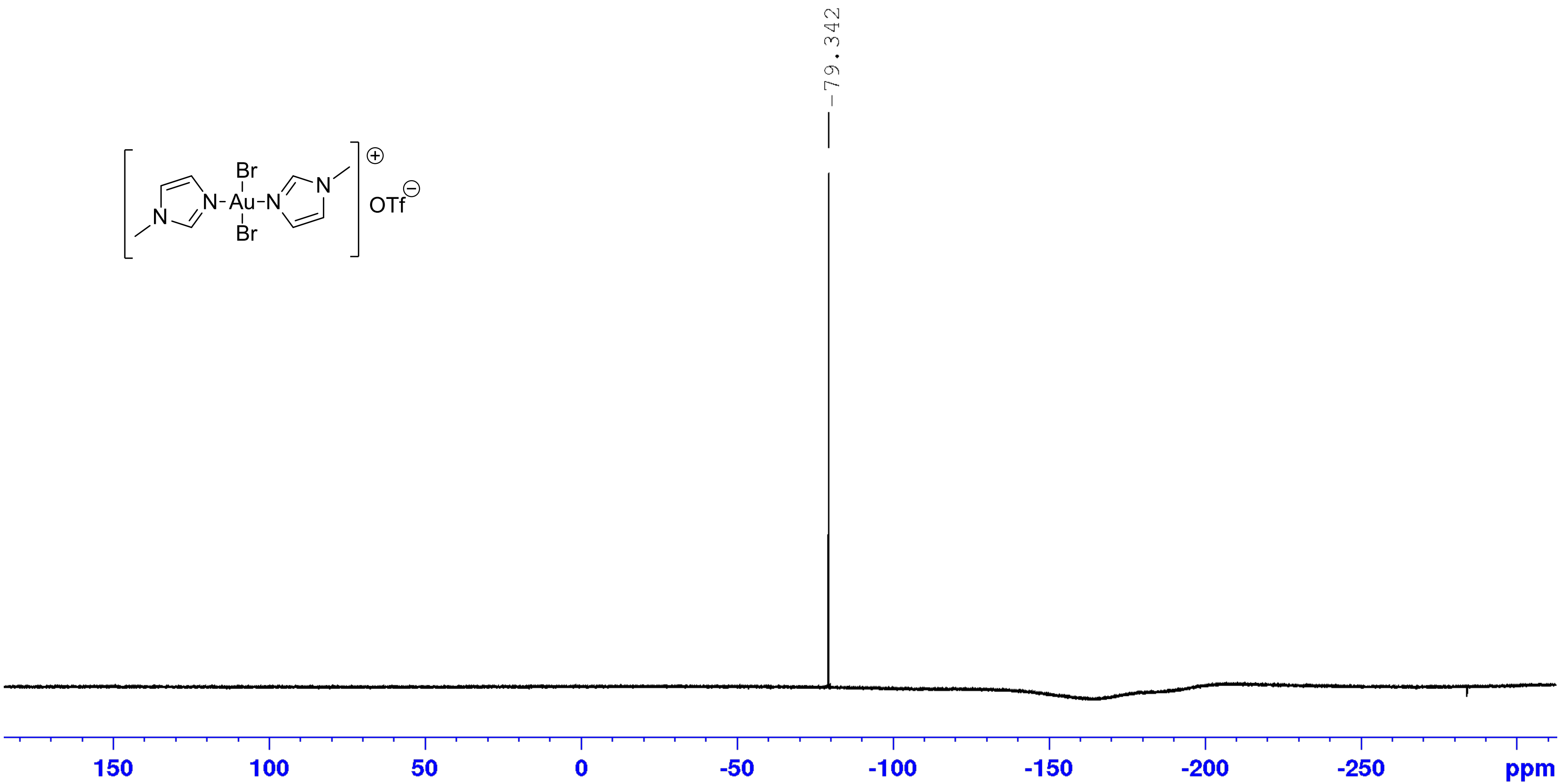

Figure S14 ^{19}F NMR of [Au(MeIM)$_2$Br$_2$][OTf] in CD_3CN

[Au(MeIM)$_2$(C$_6$H$_5$)(OTf)][NTf$_2$] – CD$_2$Cl$_2$

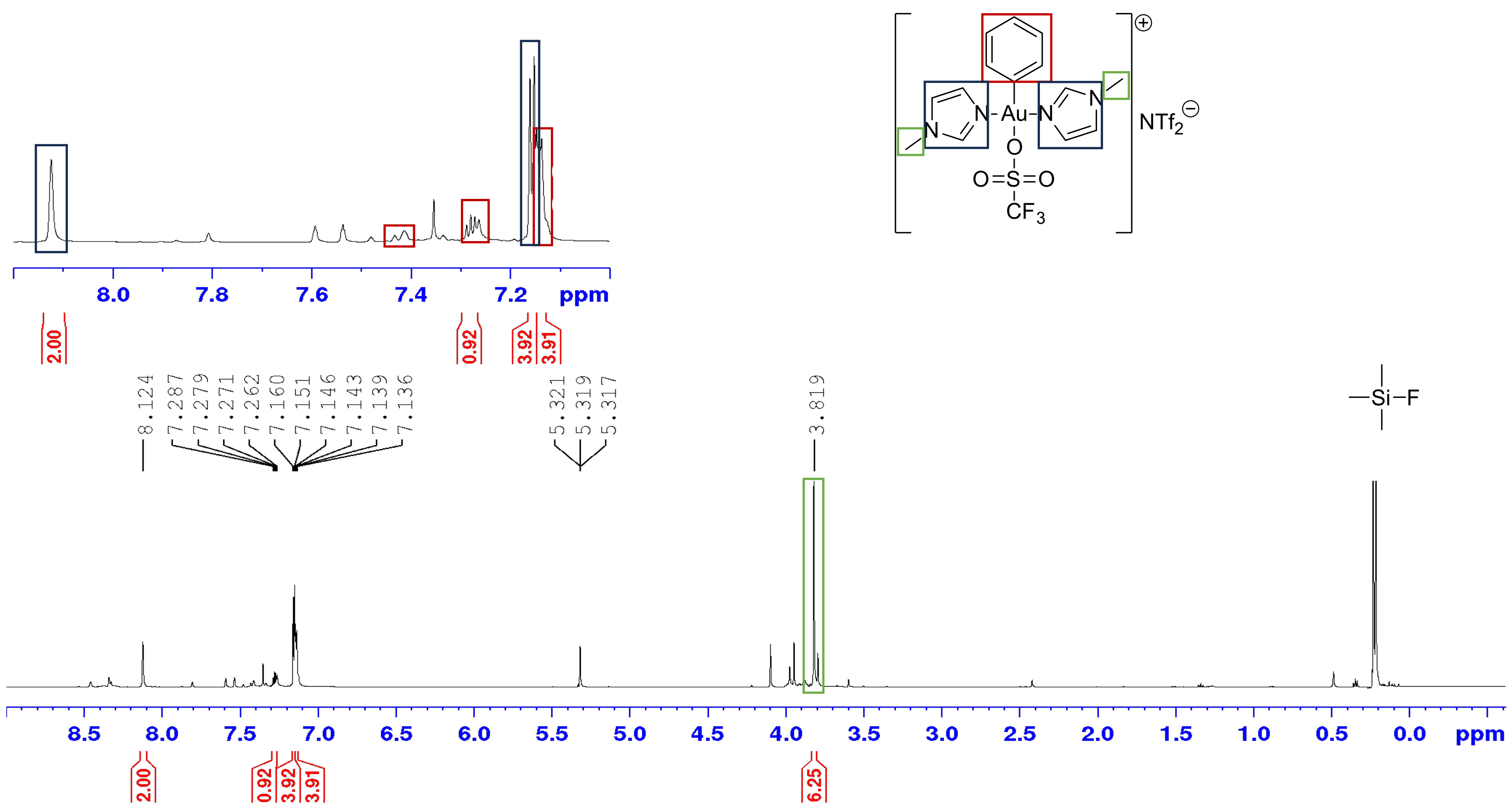

Figure S15 ^{1}H NMR of [Au(MeIM)$_2$(C$_6$H$_5$)(OTf)][NTf$_2$] in CD$_2$Cl$_2$, partial overlap of benzene multiplet with imidazole backbone

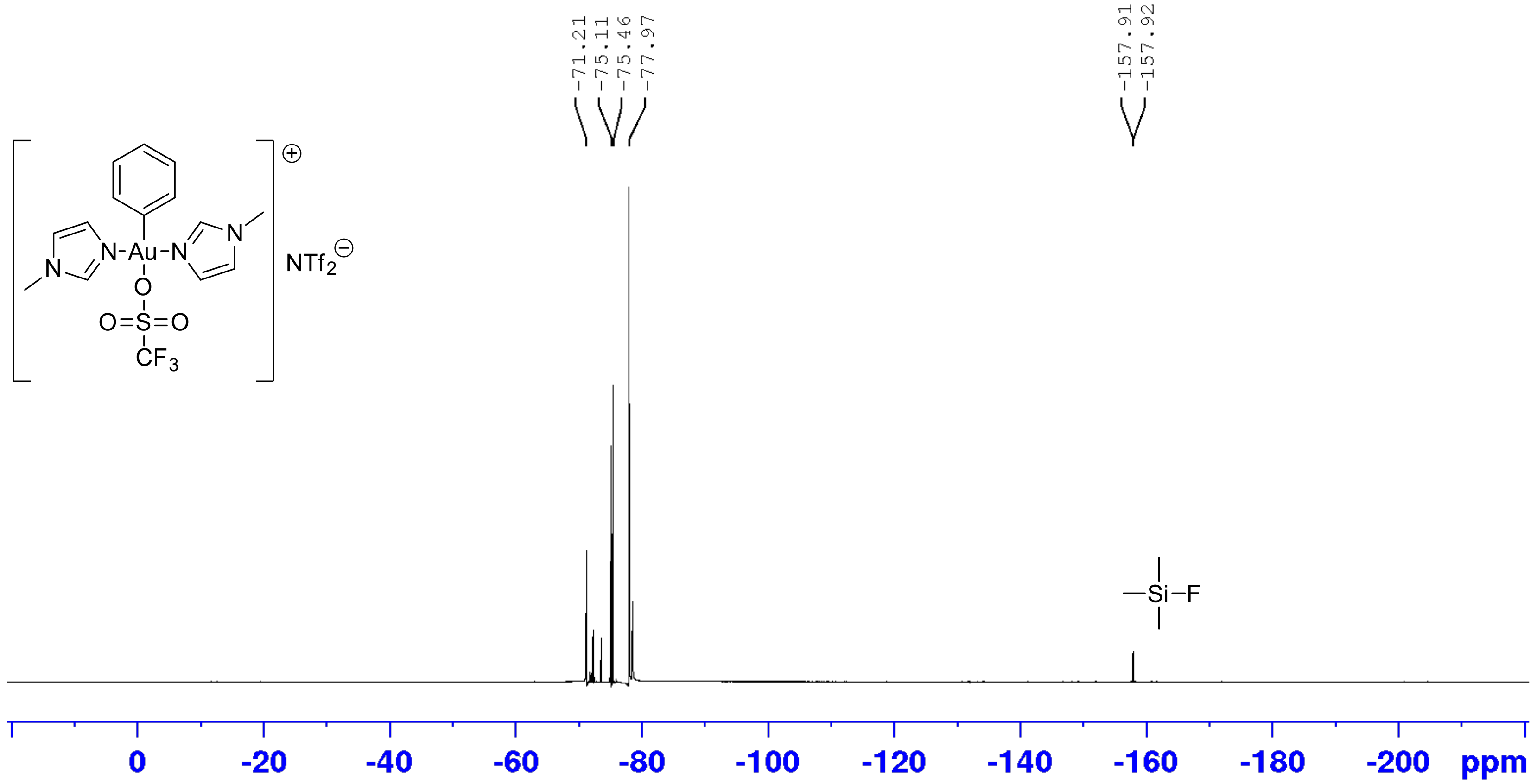

Figure S16 ^{19}F NMR of [Au(MeIM)$_2$(C$_6$H$_5$)(OTf)][NTf$_2$] in CD$_2$Cl$_2$

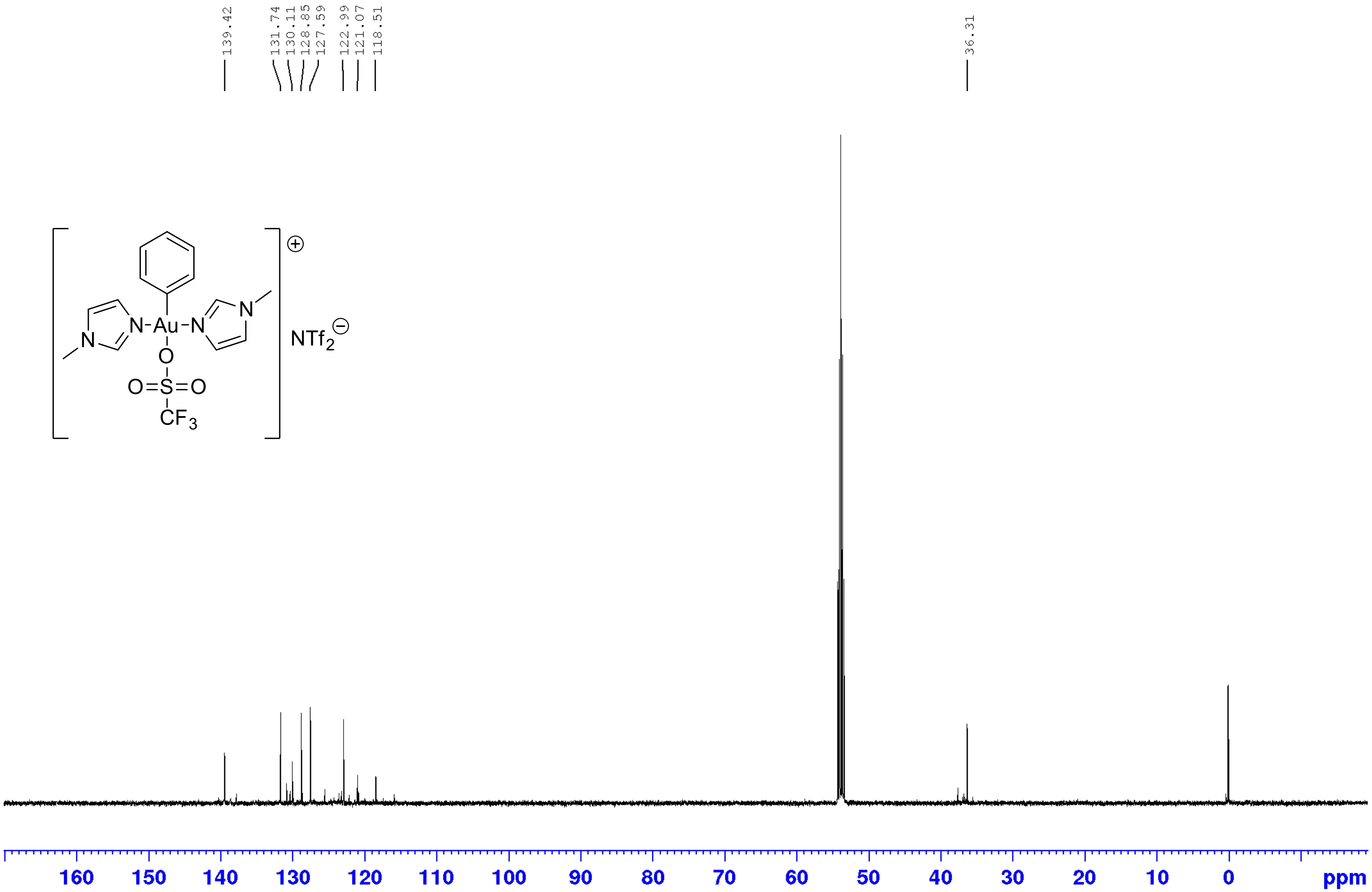

Figure S17 ^{13}C NMR of $[Au(MeIM)_2(C_6H_5)(OTf)][NTf_2]$ in CD_2Cl_2

[Au(MeIM)$_2$(C$_6$H$_4$(CH$_3$))(OTf)][NTf$_2$] – CD$_2$Cl$_2$

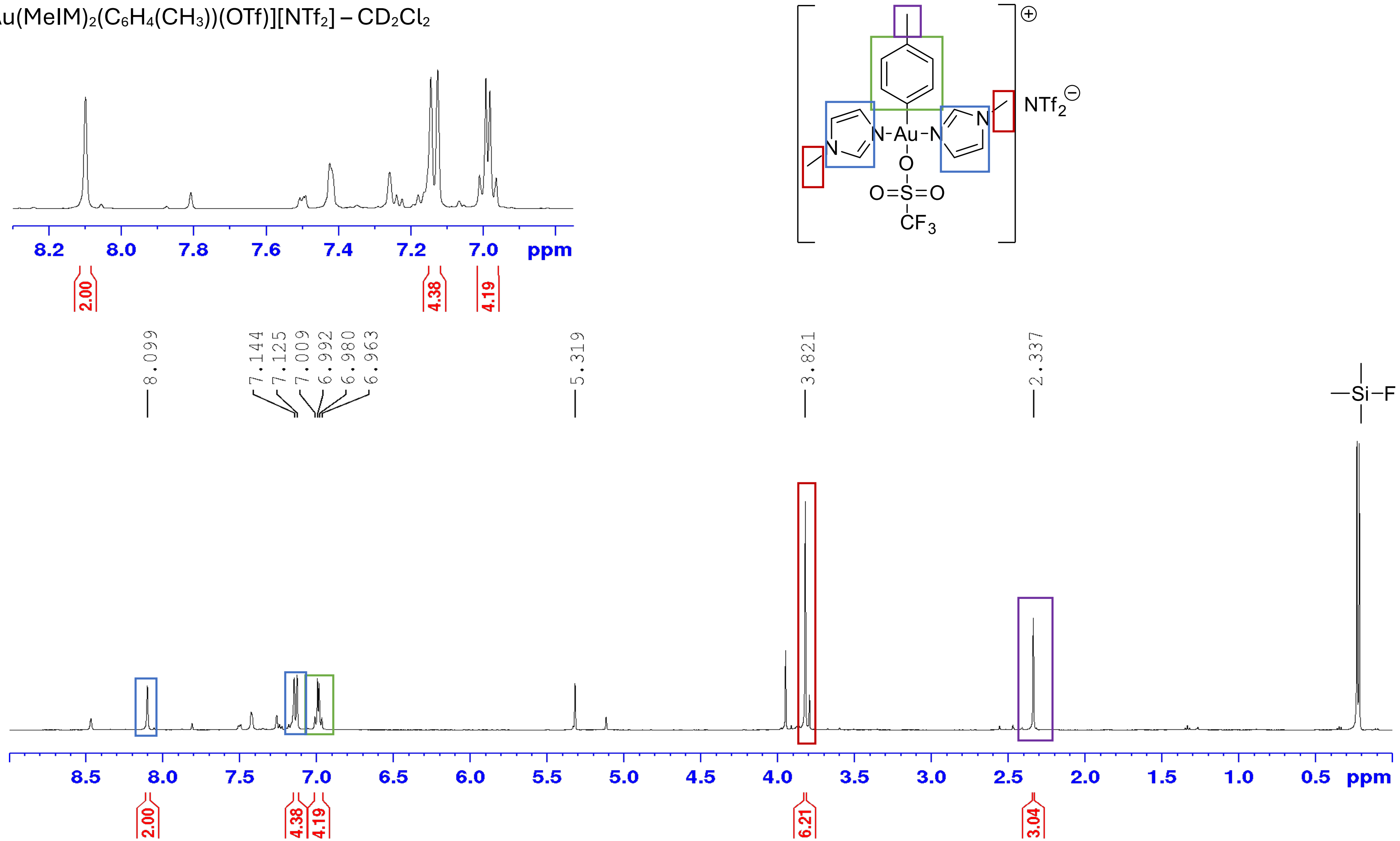

Figure S18 ^{1}H NMR of [Au(MeIM)$_2$(C$_6$H$_4$(CH$_3$))(OTf)][NTf$_2$] in CD$_2$Cl$_2$

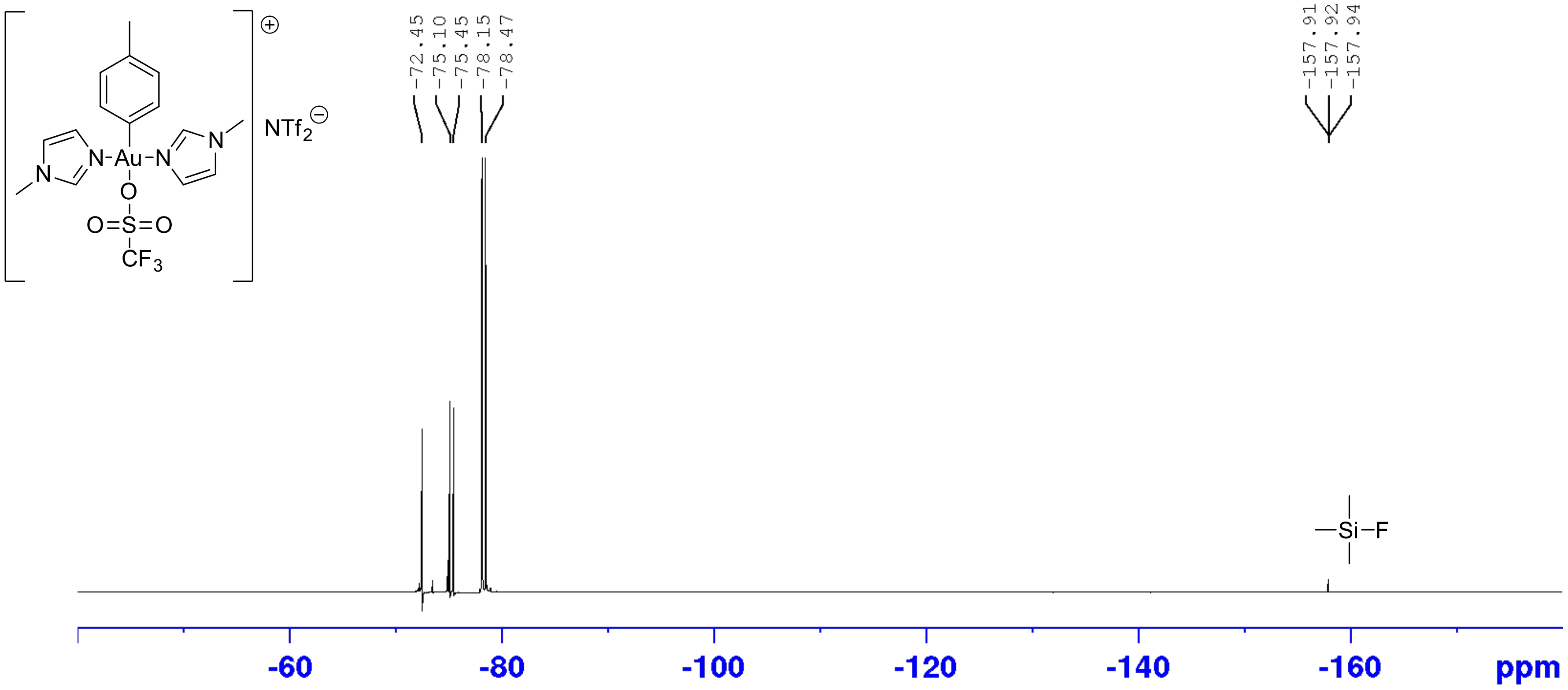

Figure S19 ^{19}F NMR of [Au(MeIM)$_2$(C$_6$H$_4$(CH$_3$))(OTf)][NTf$_2$] in CD$_2$Cl$_2$

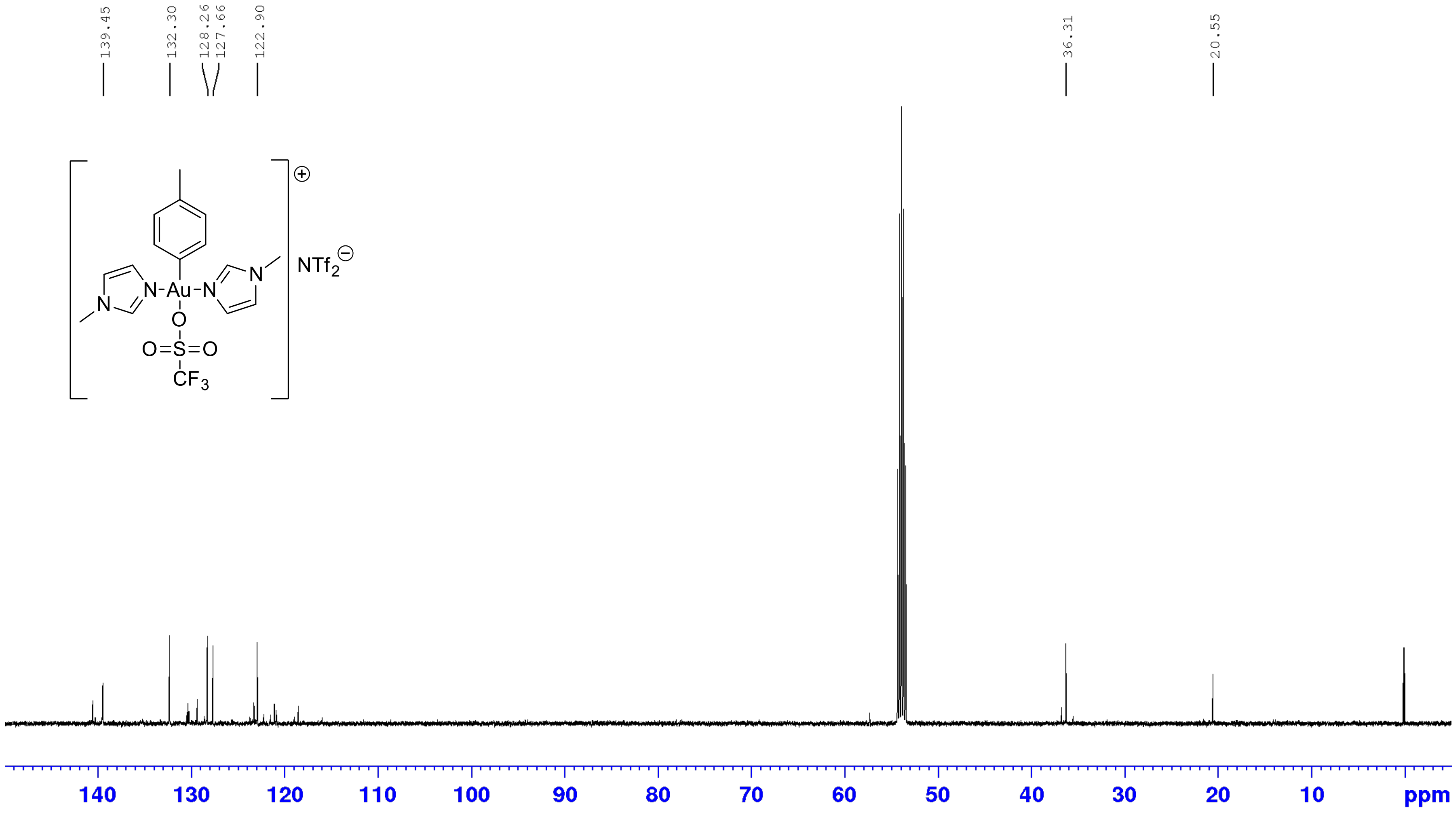

Figure S20 ^{13}C NMR of $[Au(MeIM)_2(C_6H_4(CH_3))(OTf)][NTf_2]$ in CD_2Cl_2

Reaction of [Au(MeIM)$_2$(C$_6$H$_4$(CH$_3$))(OTf)][NTf$_2$] + [NBu$_4$][I] - CD$_2$Cl$_2$

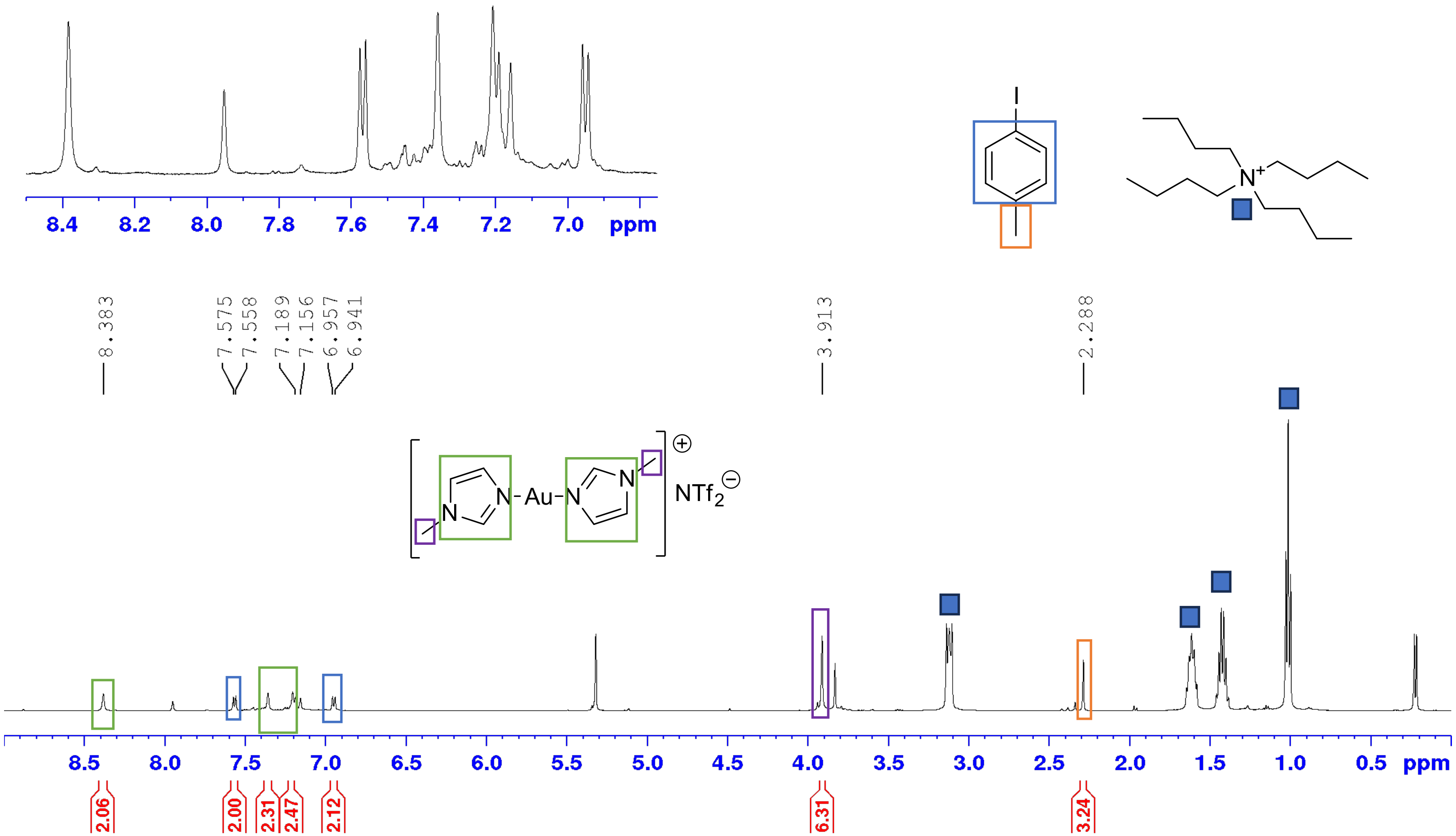

Figure S21 ^{1}H NMR of reaction with [Au(MeIM)$_2$(C$_6$H$_4$(CH$_3$))(OTf)] and [NBu$_4$][I] in CD$_2$Cl$_2$, showing [Au(MeIM)$_2$][OTf] and p-iodotoluene products[4]

Reaction of [Au(MeIM)$_2$(C$_6$H$_4$(CH$_3$))(OTf)][NTf$_2$] + [NBu$_4$][Br] – CDCl$_3$

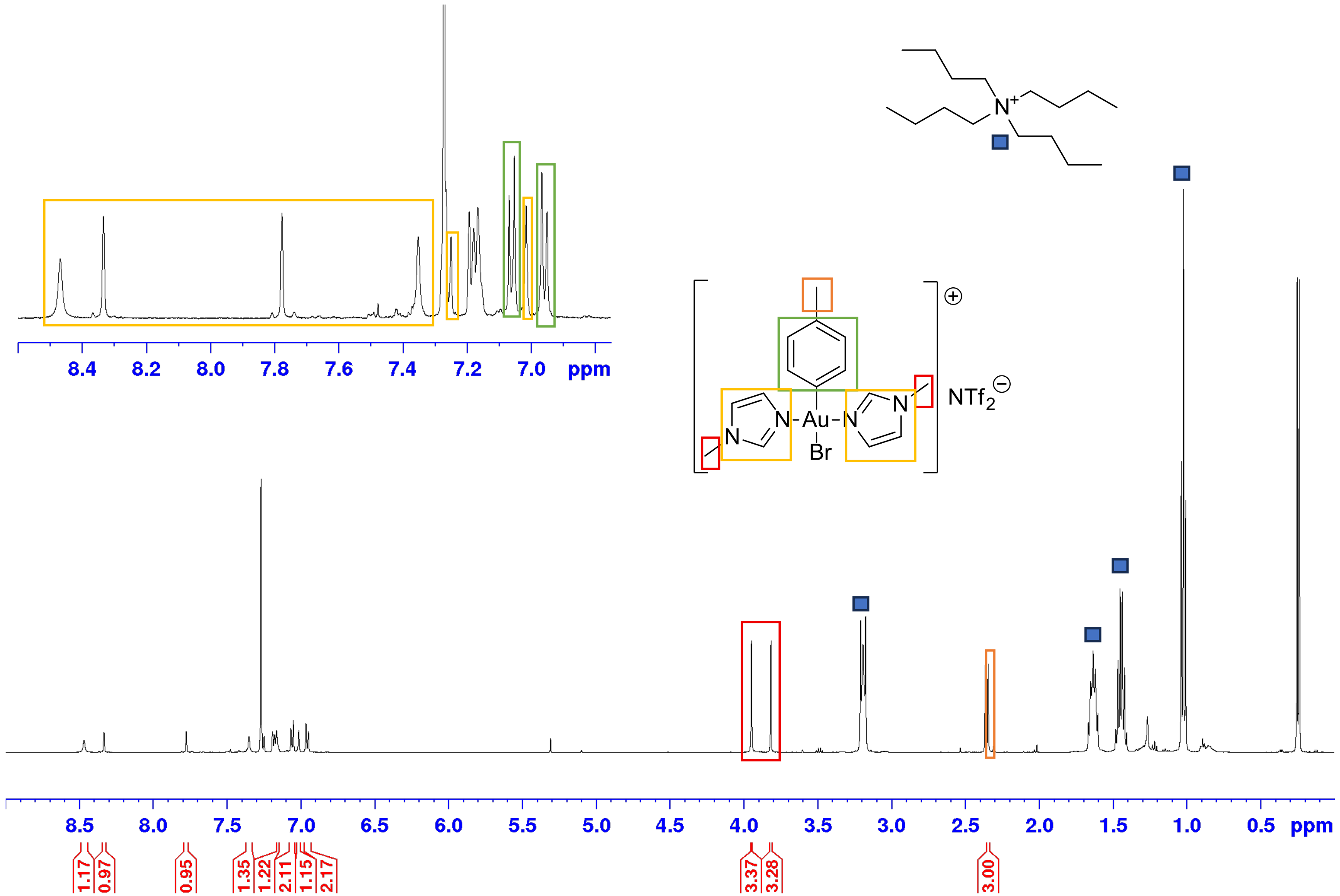

Figure S22 ^{1}H NMR of reaction with [Au(MeIM)$_2$(C$_6$H$_4$(CH$_3$))(OTf)][NTf$_2$] and [NBu$_4$][Br] in CDCl$_3$ showing [Au(MeIM)$_2$(Br)(C$_6$H$_4$(CH$_3$))][NTf$_2$].

Reaction of [Au(MeIM)$_2$(C$_6$H$_4$(CH$_3$))(OTf)][NTf$_2$] +[NBu$_4$][Cl] – CD$_2$Cl$_2$

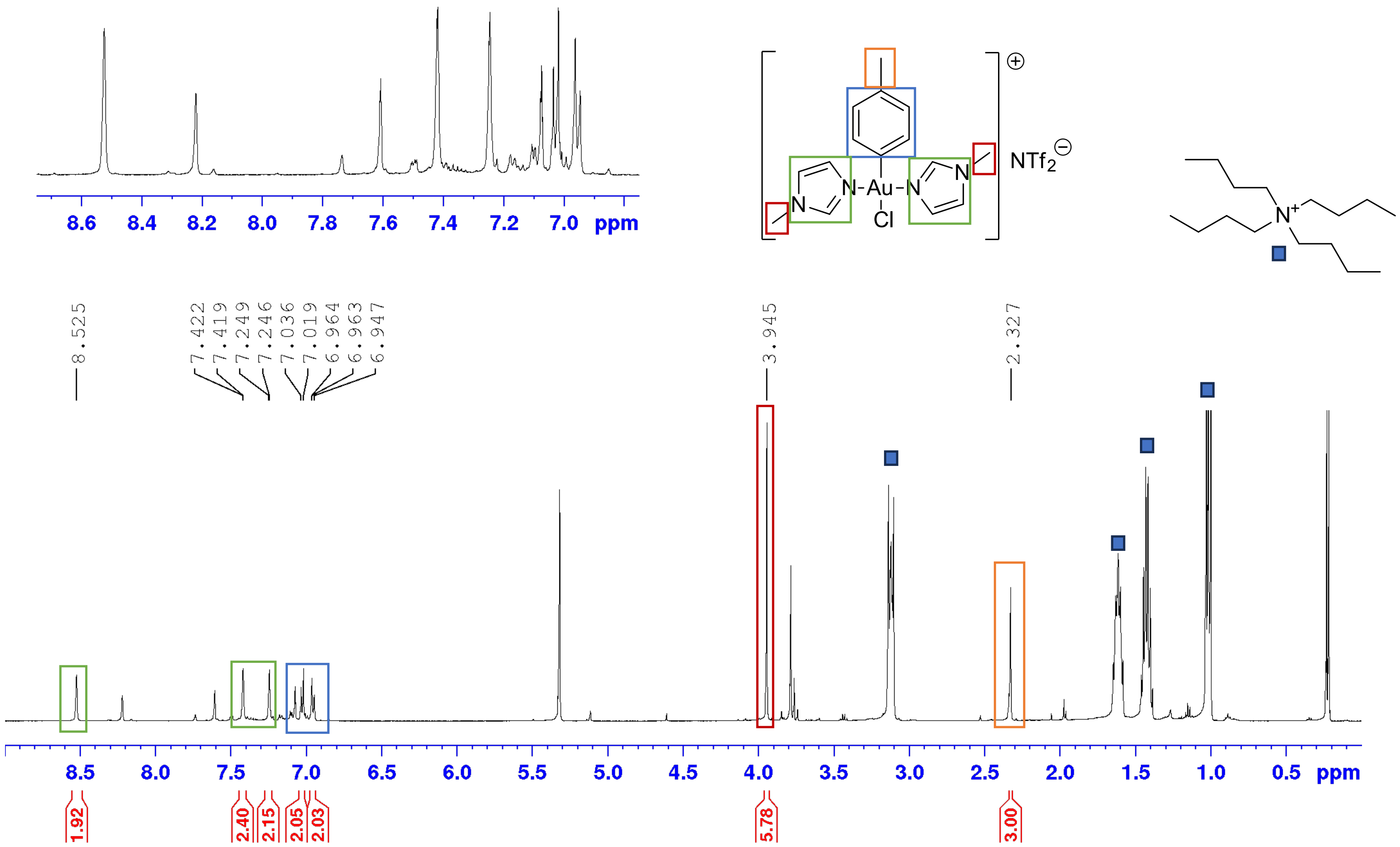

Figure S23 ^{1}H NMR of reaction with [Au(MeIM)$_2$(C$_6$H$_4$(CH$_3$))(OTf)][NTf$_2$] and [NBu$_4$][Cl] in CD$_2$Cl$_2$ showing [Au(MeIM)$_2$(Cl)(C$_6$H$_4$(CH$_3$))][NTf$_2$]

Reaction of [Au(MeIM)$_2$F$_2$][OTf] with one equivalent of TMS-OTf in CD$_3$CN

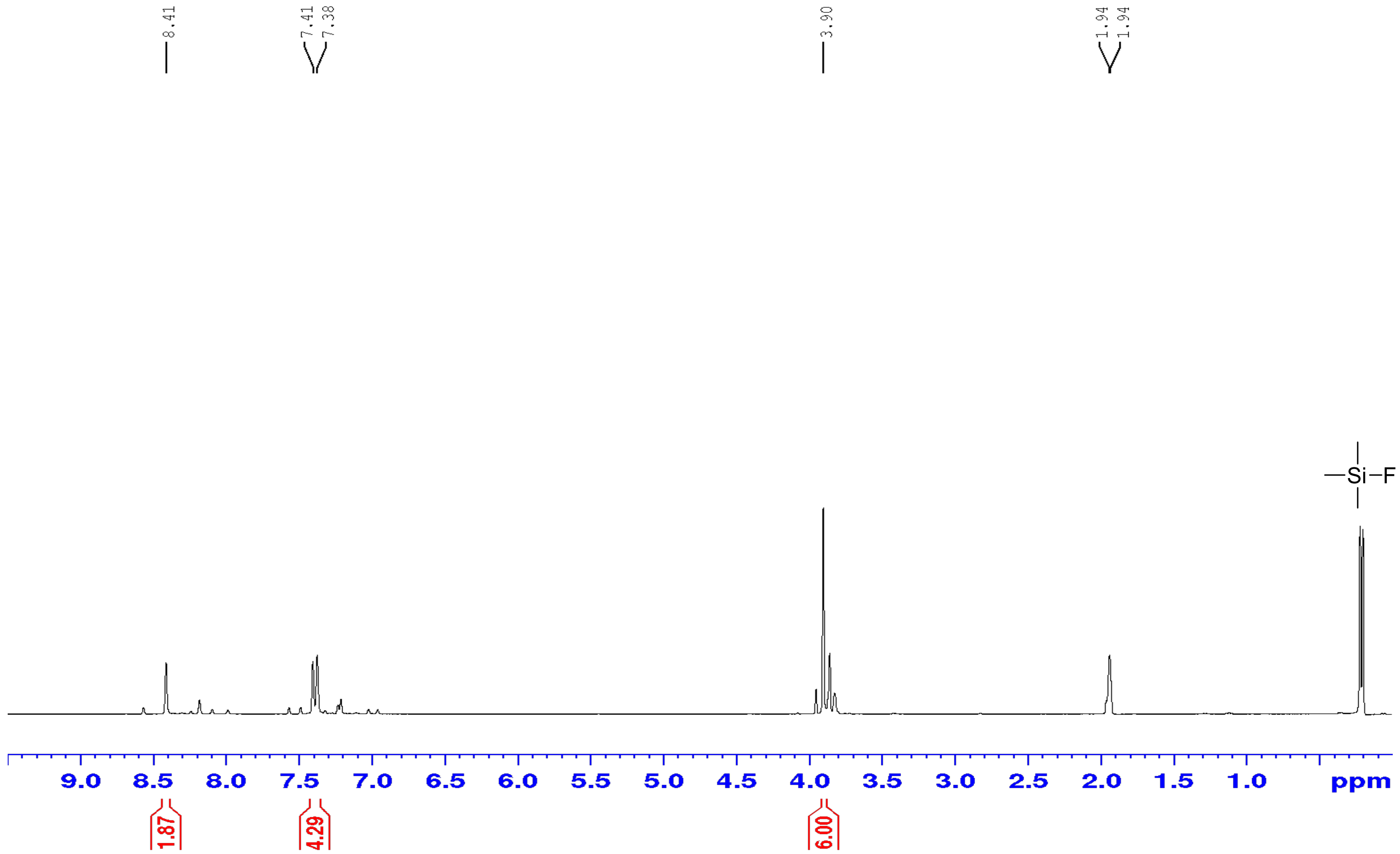

Figure S24 ^{1}H NMR of Reaction of [Au(MeIM)$_2$F$_2$][OTf] with one equivalent of TMS-OTf in CD$_3$CN

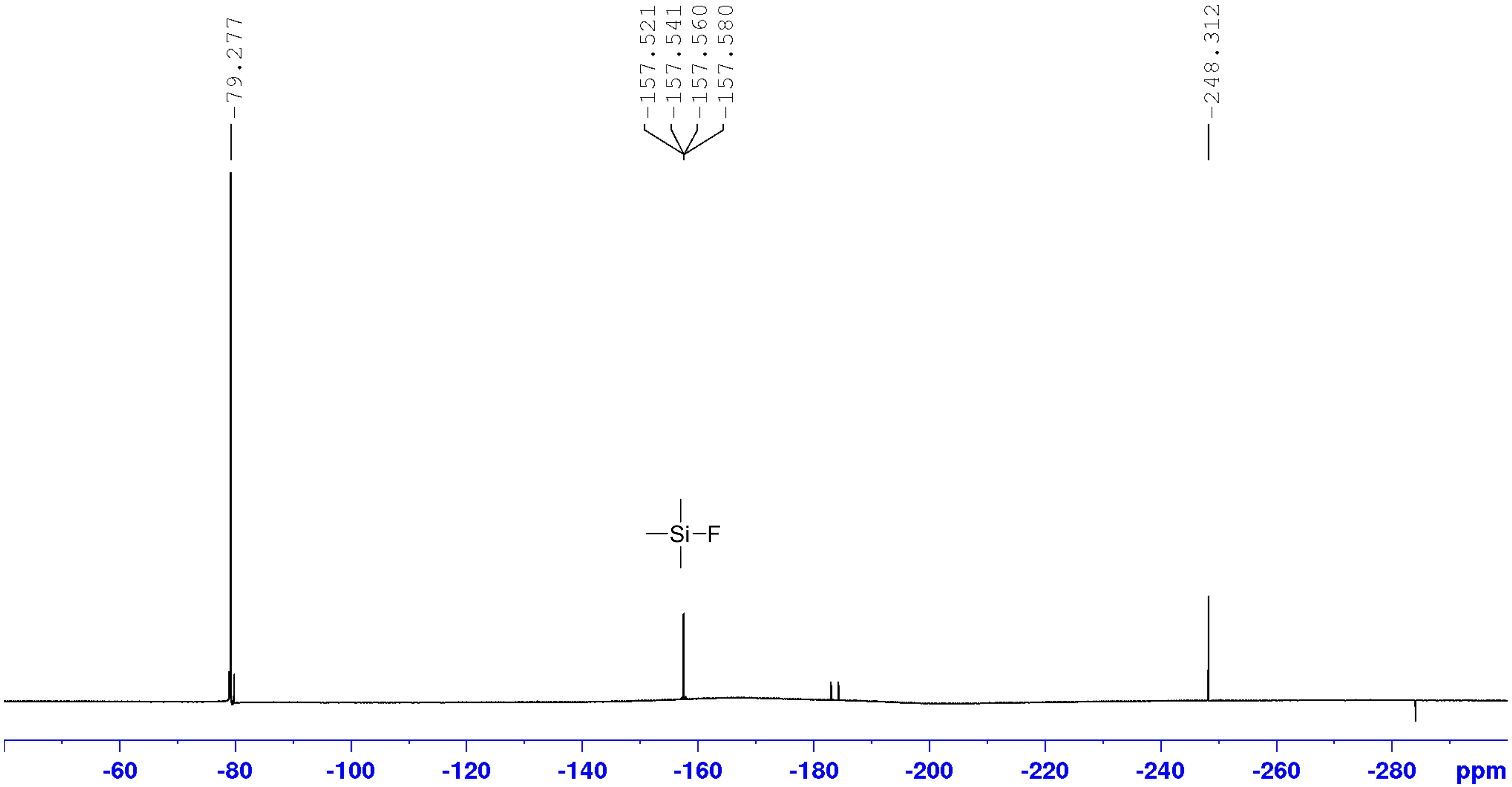

Figure S25 ^{19}F NMR of Reaction of $[Au(MeIM)_2F_2][OTf]$ with one equivalent of TMS-OTf in CD_3CN

[Au(MeIM)$_2$(MeCN)(Mes)][OTf]$_2$ + [NBu$_4$][Br] – 4 Days 55 °C – CD_3CN

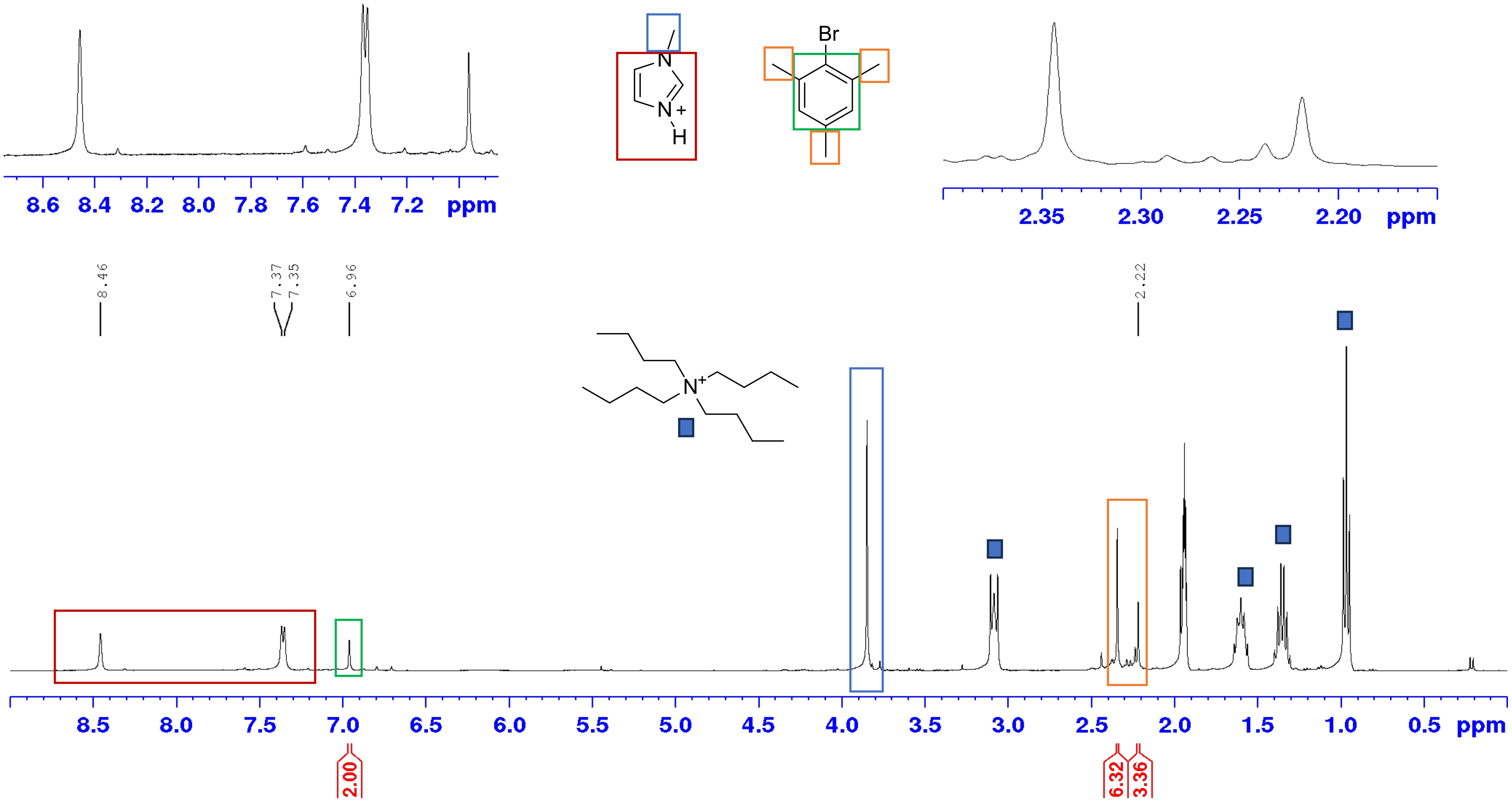

Figure S26 ^{1}H NMR of reaction with [Au(MeIM)$_2$(MeCN)(Mes)][OTf]$_2$ and [NBu$_4$][Br] in CD_3CN, bromomesitylene product peaks picked and integrated after 4 days at 55 °C

[Au(MeIM)$_2$(MeCN)(Mes)][OTf]$_2$ + [NBu$_4$][Cl] – 4 Days 55 °C – CD$_3$CN

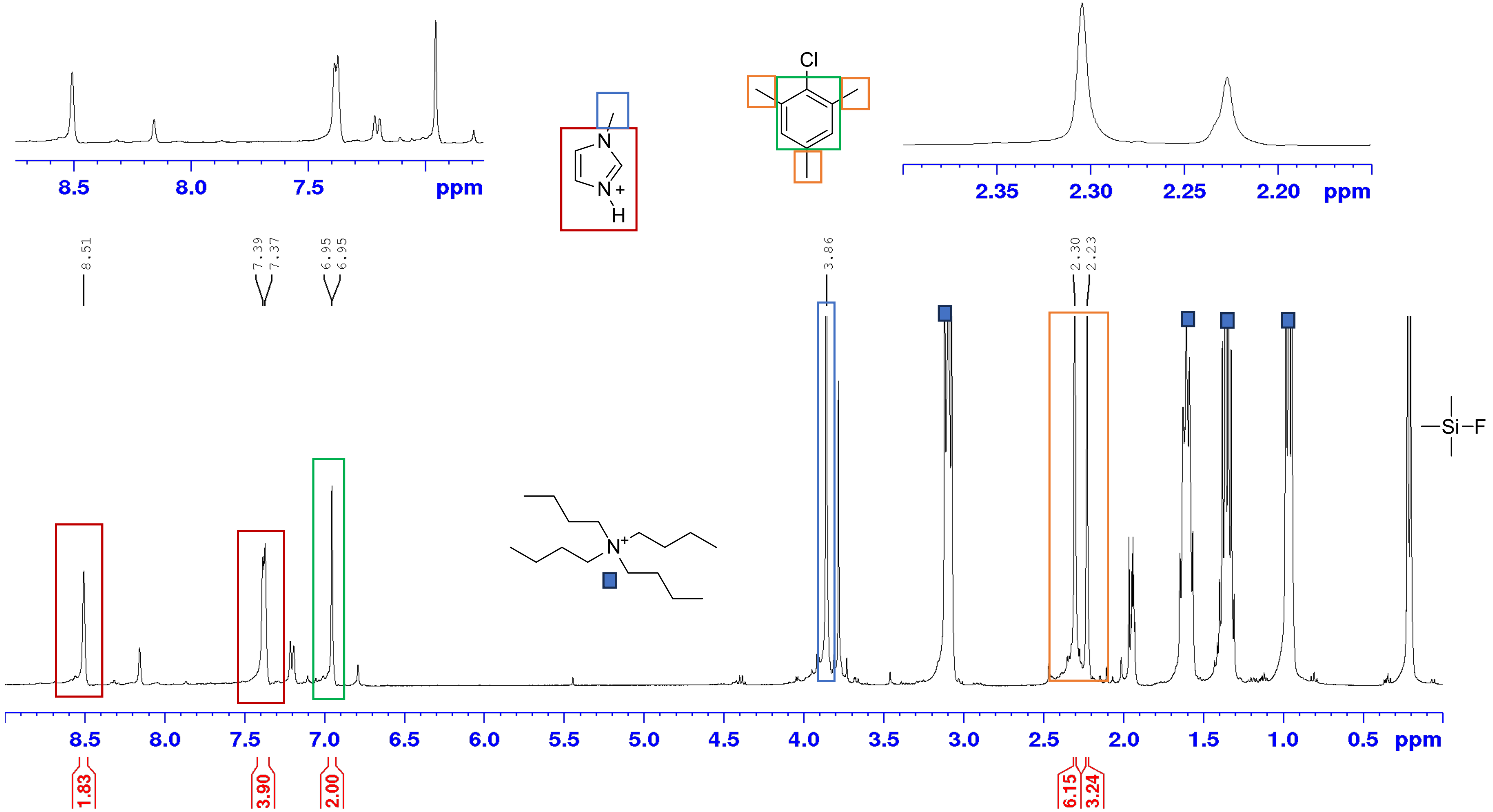

Figure S27 ^{1}H NMR of reaction with [Au(MeIM)$_2$(MeCN)(Mes)][OTf]$_2$ and [NBu$_4$][Cl] in CD$_3$CN, chloromesitylene product peaks picked and integrated after 4 days at 55 °C

[Au(MeIM)$_2$(I)(Mes)][OTf] – CD_2Cl_2

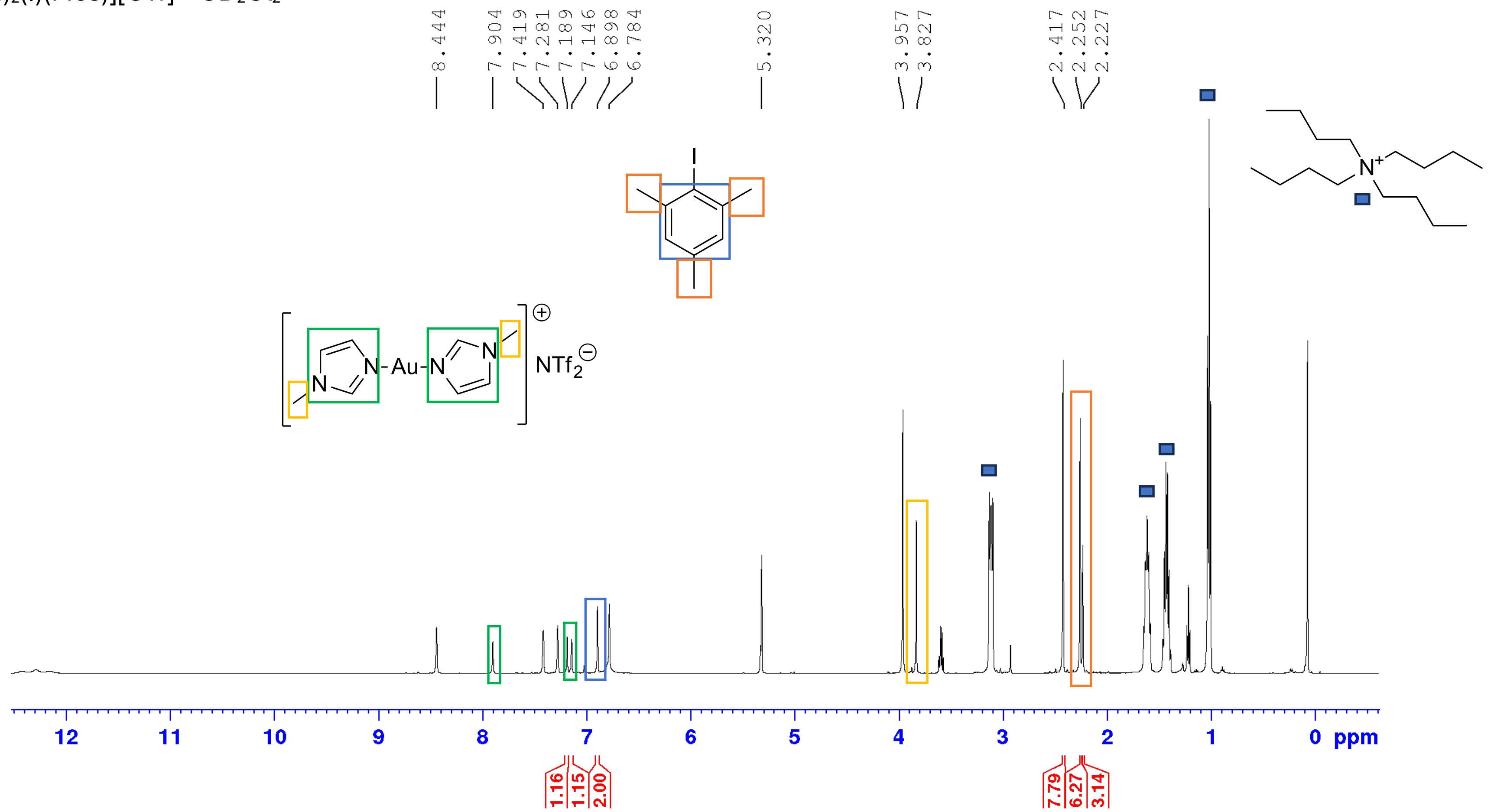

Figure S28 ^{1}H NMR of reaction with [Au(MeIM)$_2$(NTf$_2$)$_2$][OTf] with [NBu$_4$][I] and mesitylene in CD_2Cl_2 at 16 hours.

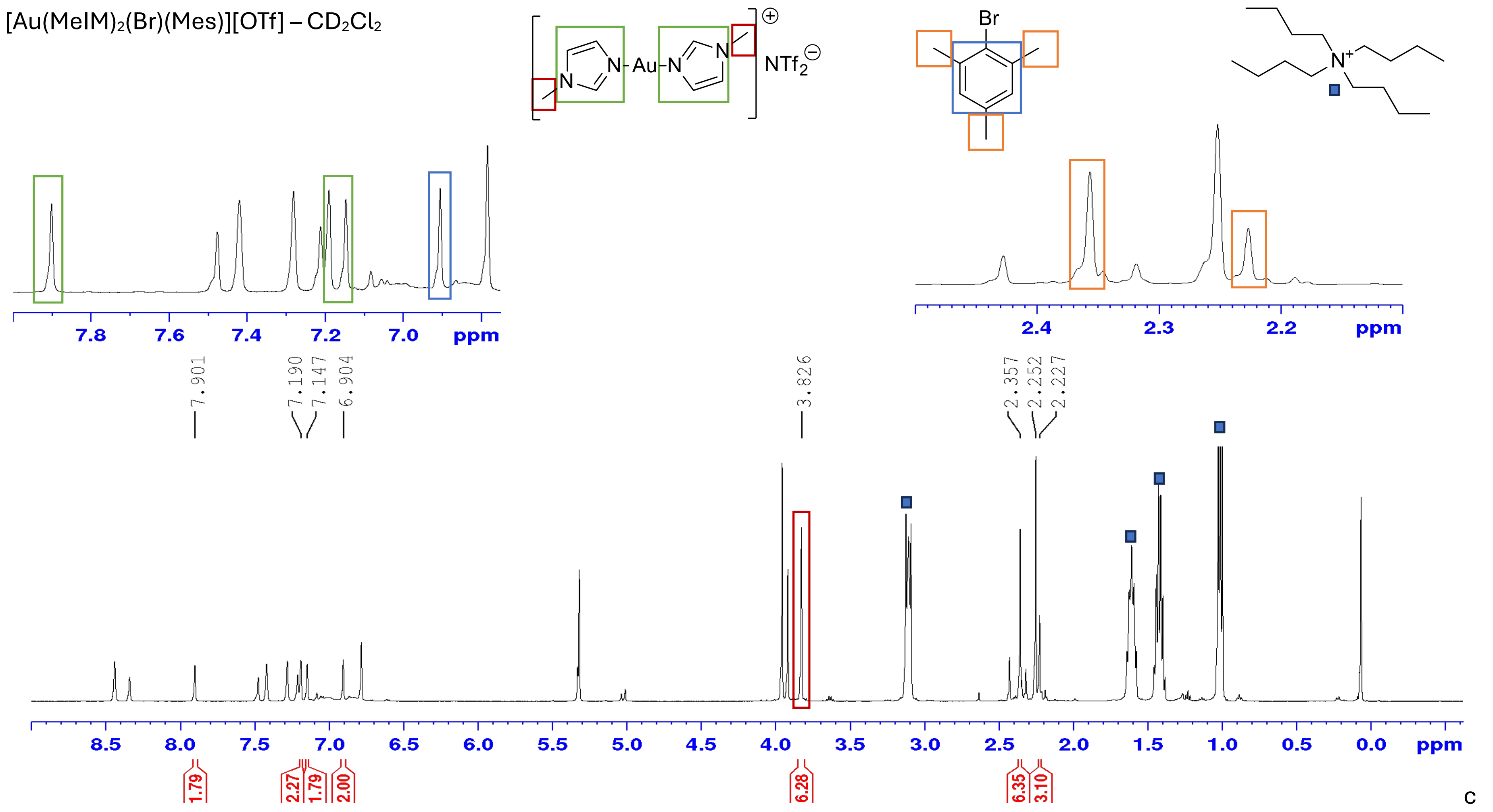

Figure S29 ^{1}H NMR of reaction with [Au(MeIM)$_2$(NTf$_2$)$_2$][OTf] with [NBu$_4$][Br] and mesitylene in CD$_2$Cl$_2$ at 24 hours.

[Au(MeIM)$_2$(C$_6$H$_4$(CH$_3$))(OTf)][NTf$_2$] + [NBu$_4$][I] – CDCl$_3$

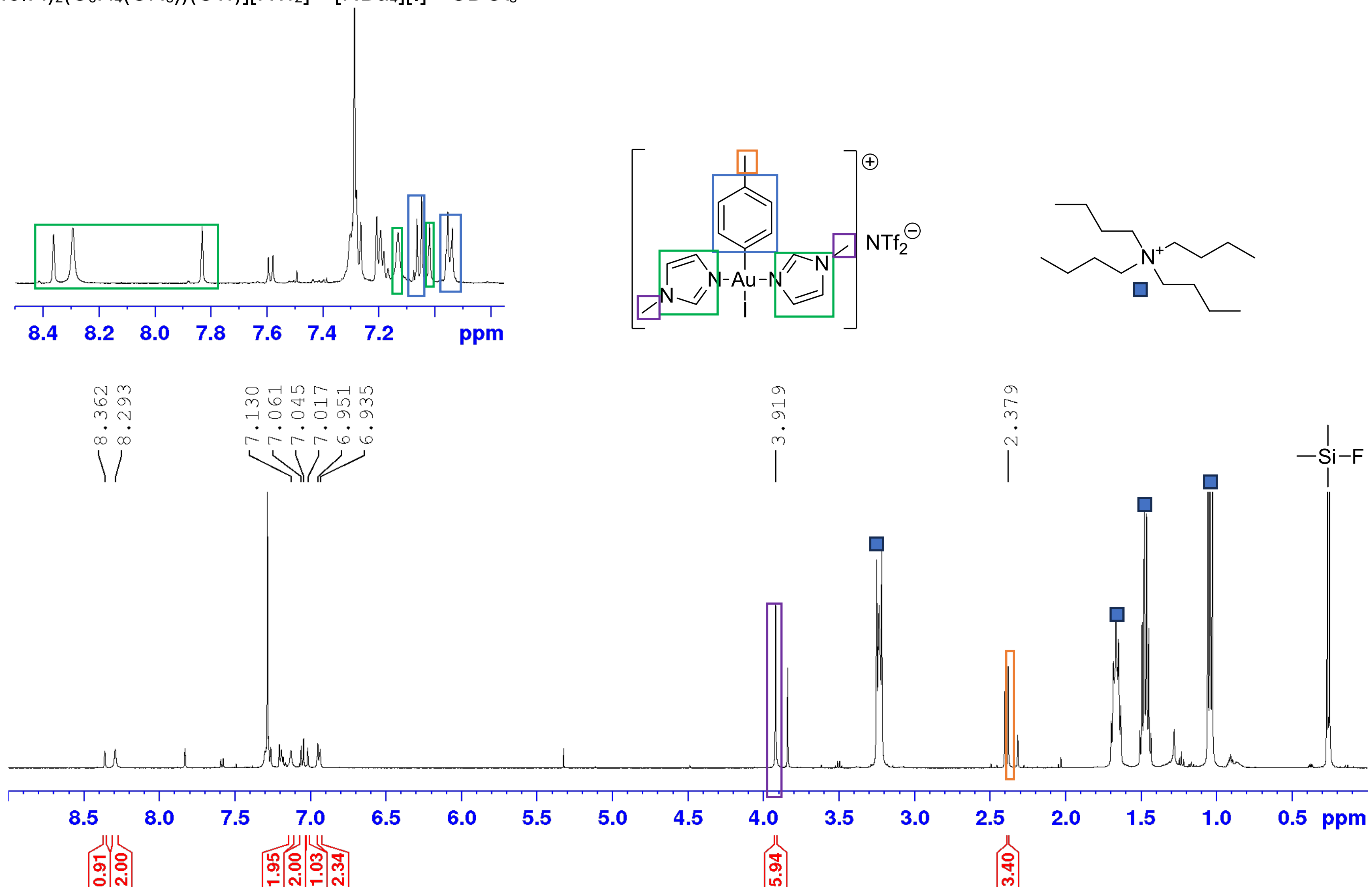

Figure S30 ^{1}H NMR of [Au(MeIM)$_2$(C$_6$H$_4$(CH$_3$))(OTf)][NTf$_2$] and [NBu$_4$][I] in CDCl$_3$ showing [Au(MeIM)$_2$(I)(C$_6$H$_4$(CH$_3$))][NTf$_2$] and small amounts of Au^I and iodotoluene.

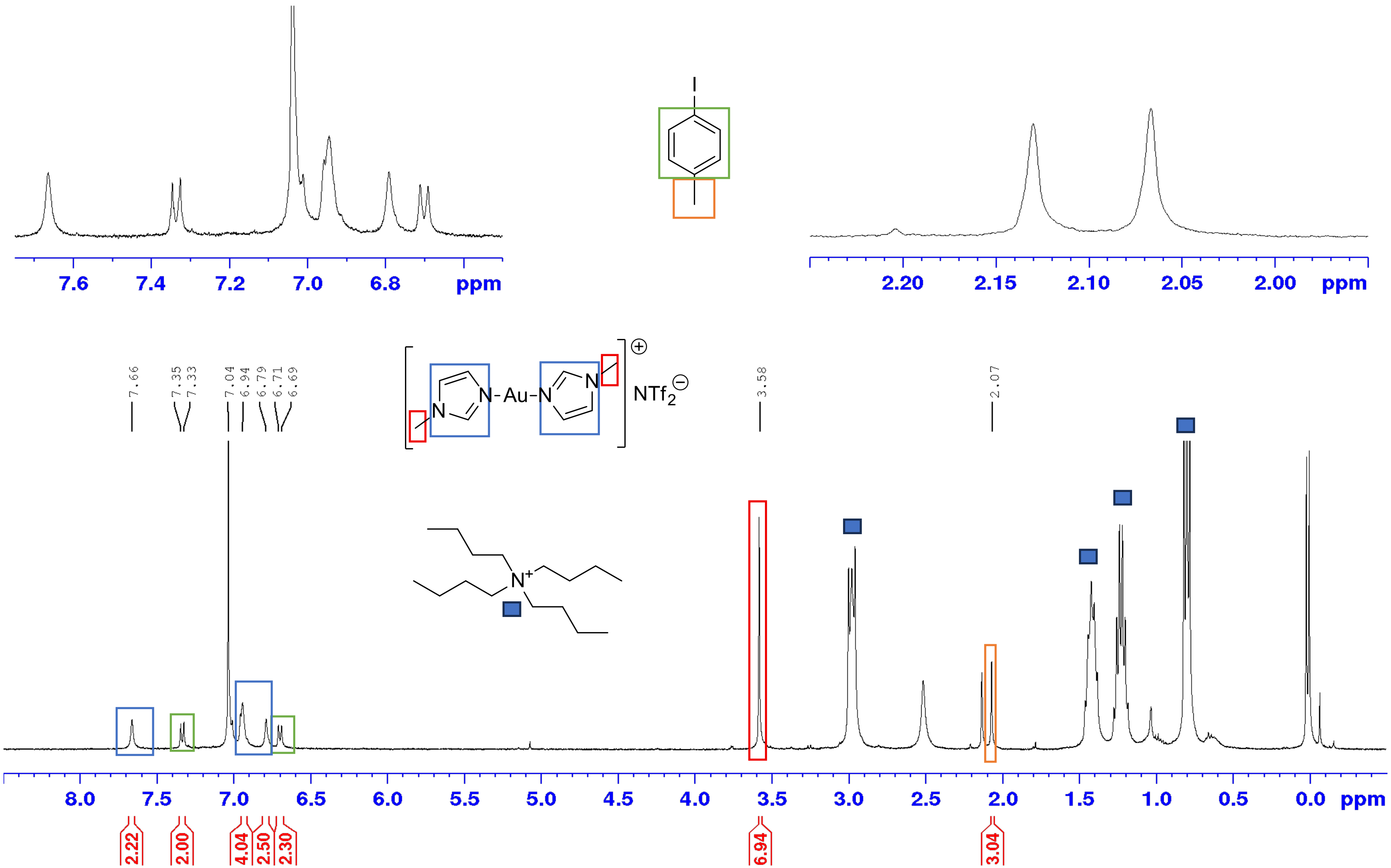

Figure S31 ^{1}H NMR of [Au(MeIM)$_2$(C$_6$H$_4$(CH$_3$))(OTf)][NTf$_2$] and [NBu$_4$][I] in CDCl$_3$ overnight at 55°C showing formation of iodotoluene

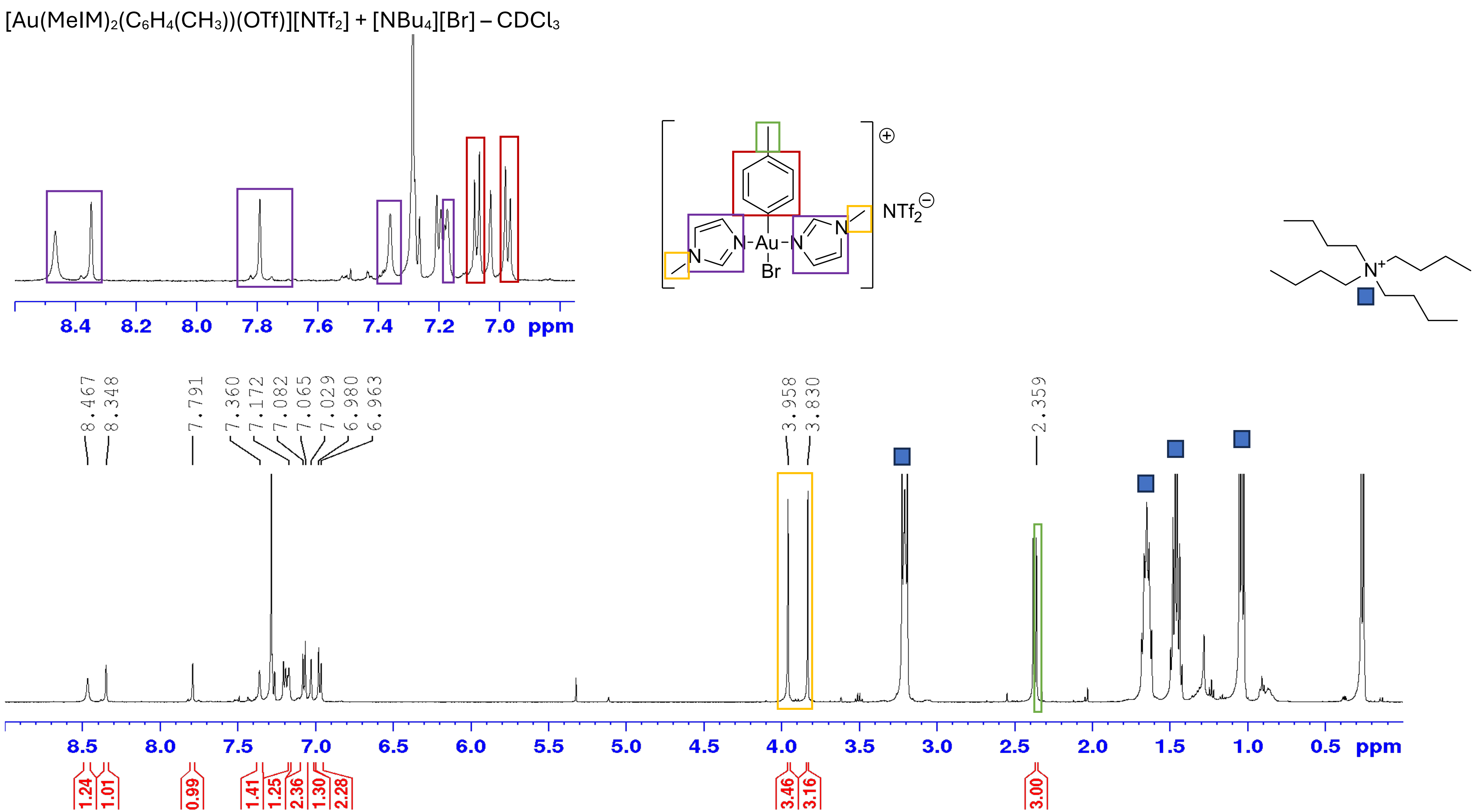

Figure S32 ^{1}H NMR of [Au(MeIM)$_2$(C$_6$H$_4$(CH$_3$))(OTf)][NTf$_2$] and [NBu$_4$][Br] in CDCl$_3$ after initial addition showing [Au(MeIM)$_2$(Br)(C$_6$H$_4$(CH$_3$))][NTf$_2$]

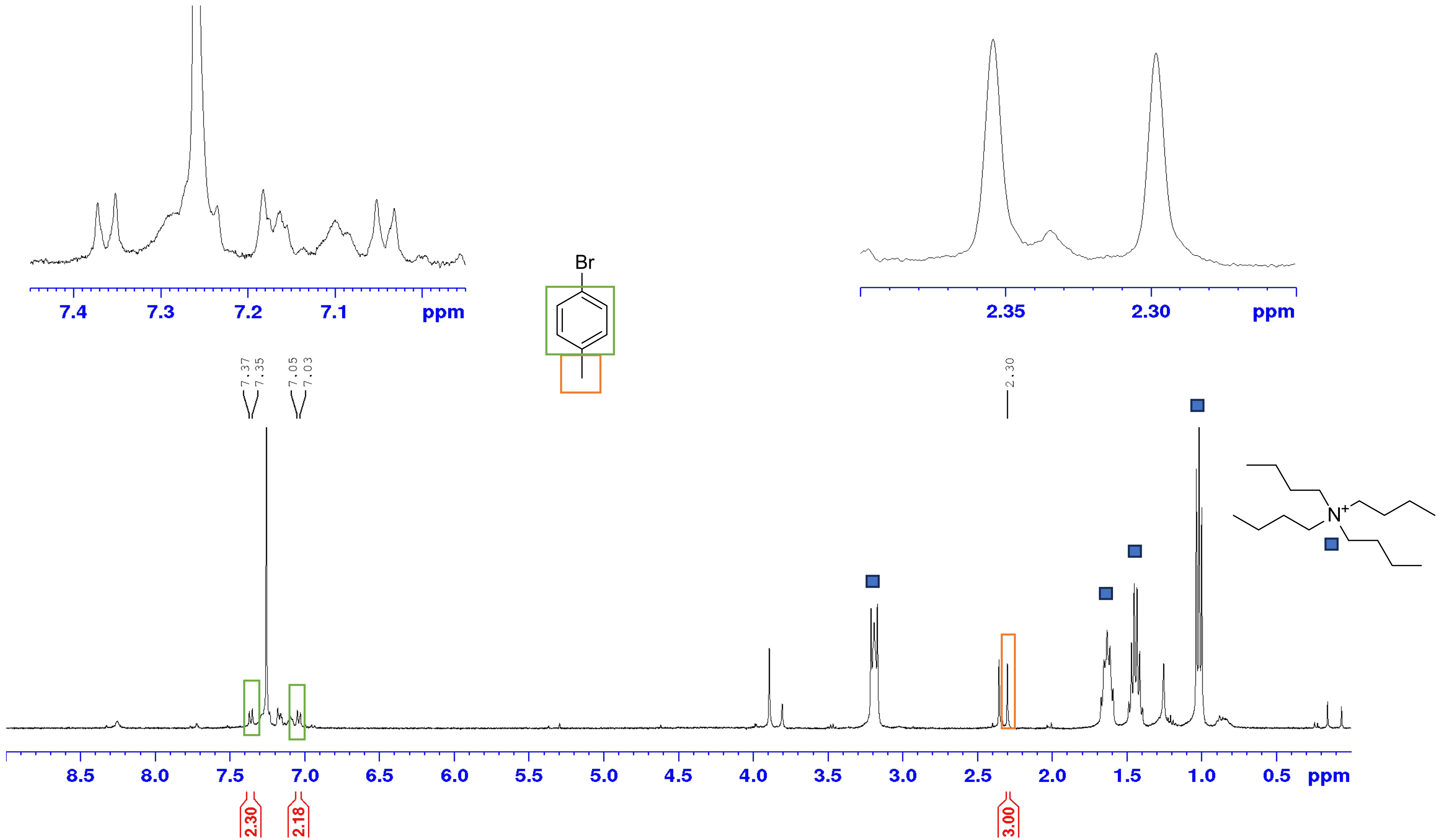

Figure S33 ^{1}H NMR of [Au(MeIM)$_2$(C$_6$H$_4$(CH$_3$))(OTf)][NTf$_2$] and [NBu$_4$][Br] in CDCl$_3$ overnight at 55°C showing formation of bromotoluene.

[Au(MeIM)$_2$(C$_6$H$_4$(CH$_3$))(OTf)][NTf$_2$] + [NBu$_4$][Cl] – CDCl$_3$

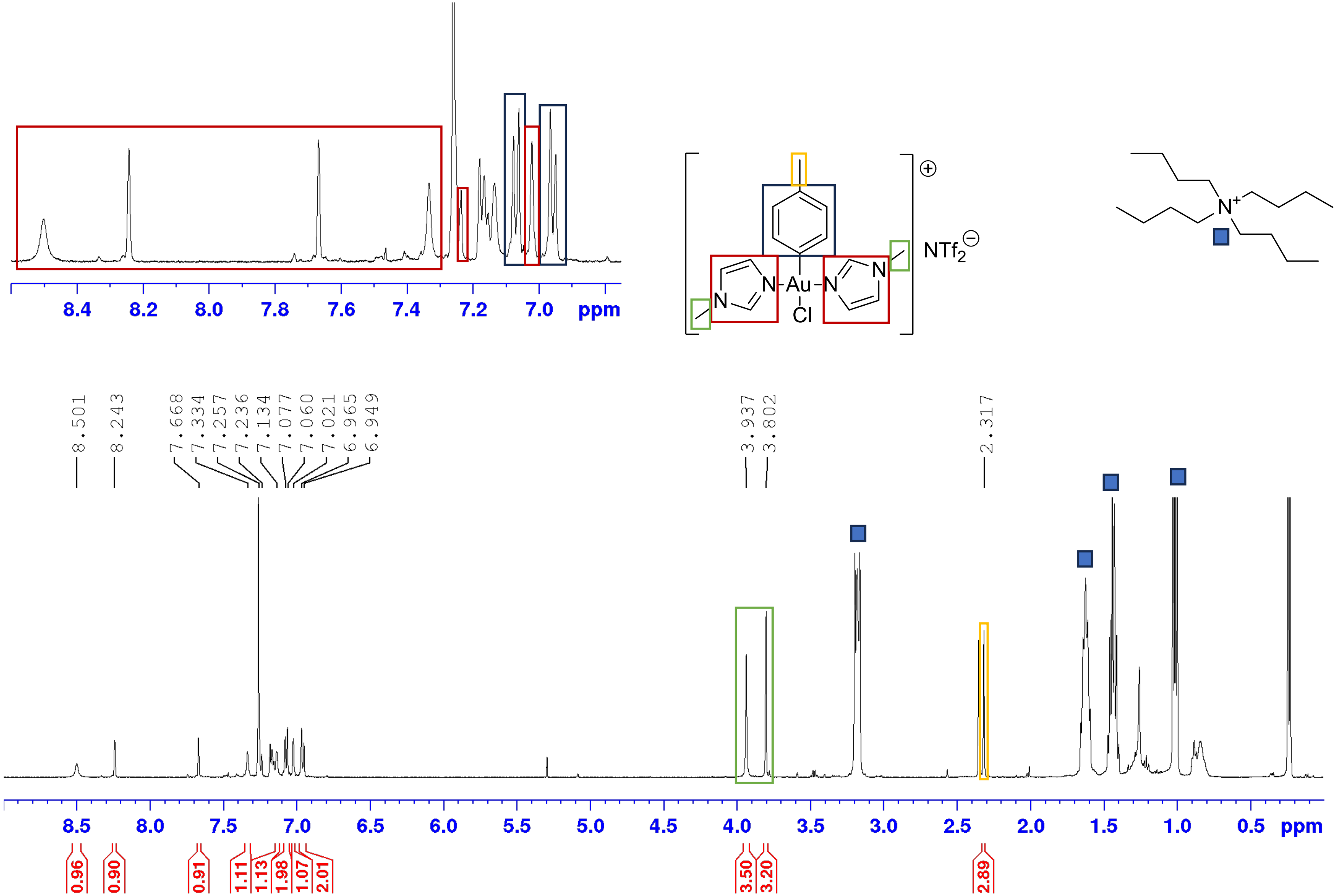

Figure S34 ^{1}H NMR of [Au(MeIM)$_2$(C$_6$H$_4$(CH$_3$))(OTf)][NTf$_2$] and [NBu$_4$][Cl] in CDCl$_3$, no degradation after heating for multiple days.

[Au(MeIM)$_2$(C$_6$H$_5$)(OTf)][NTf$_2$] + [NBu$_4$][I] – CD$_2$Cl$_2$

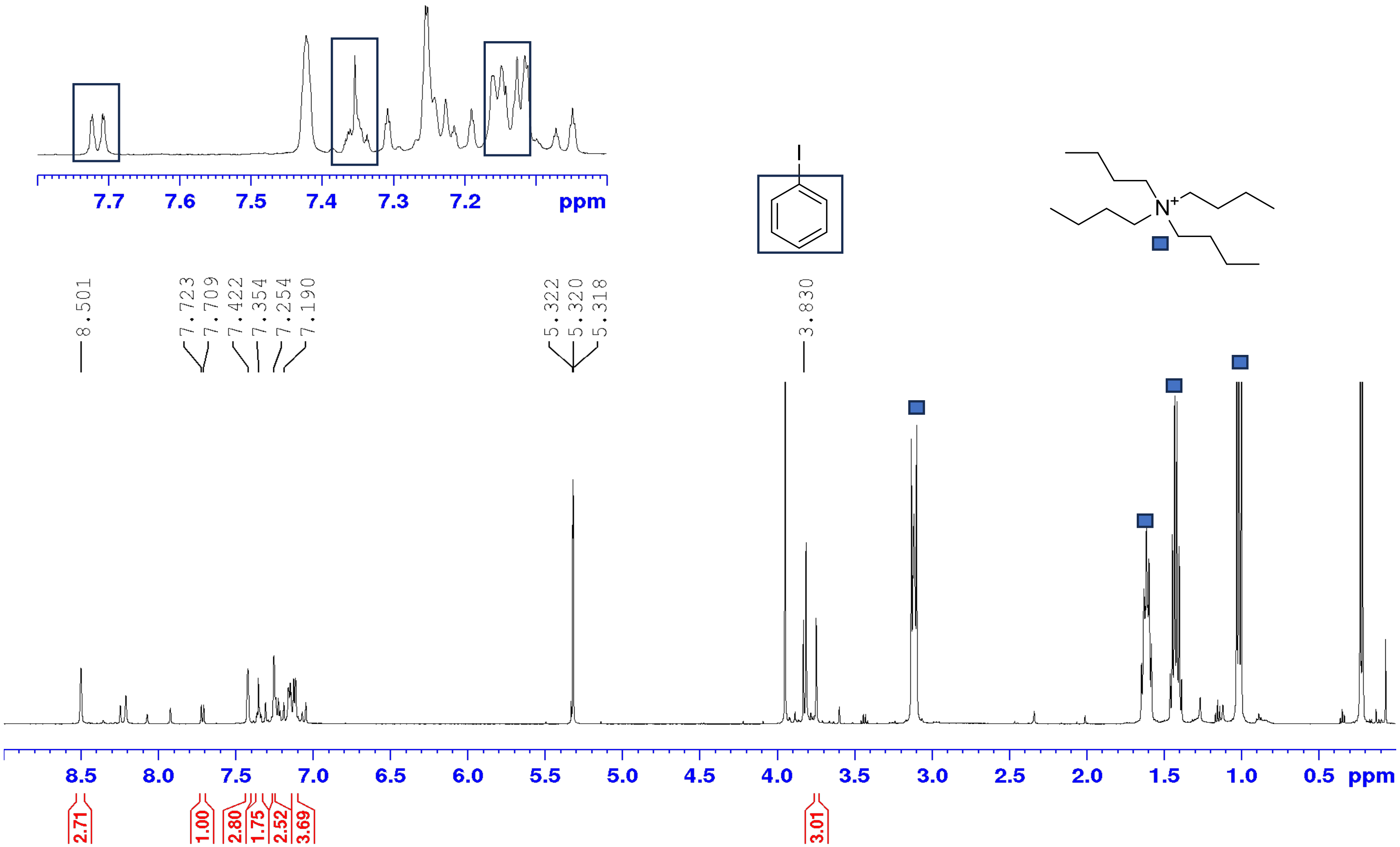

Figure S35 ^{1}H NMR for reaction of [Au(MeIM)$_2$(C$_6$H$_5$)(OTf)][NTf$_2$] and [NBu$_4$][I] at 24 hours in CD$_2$Cl$_2$, [Au(MeIM)$_2$][OTf] peaks picked as well as C$_6$H$_5$I ortho proton doublet

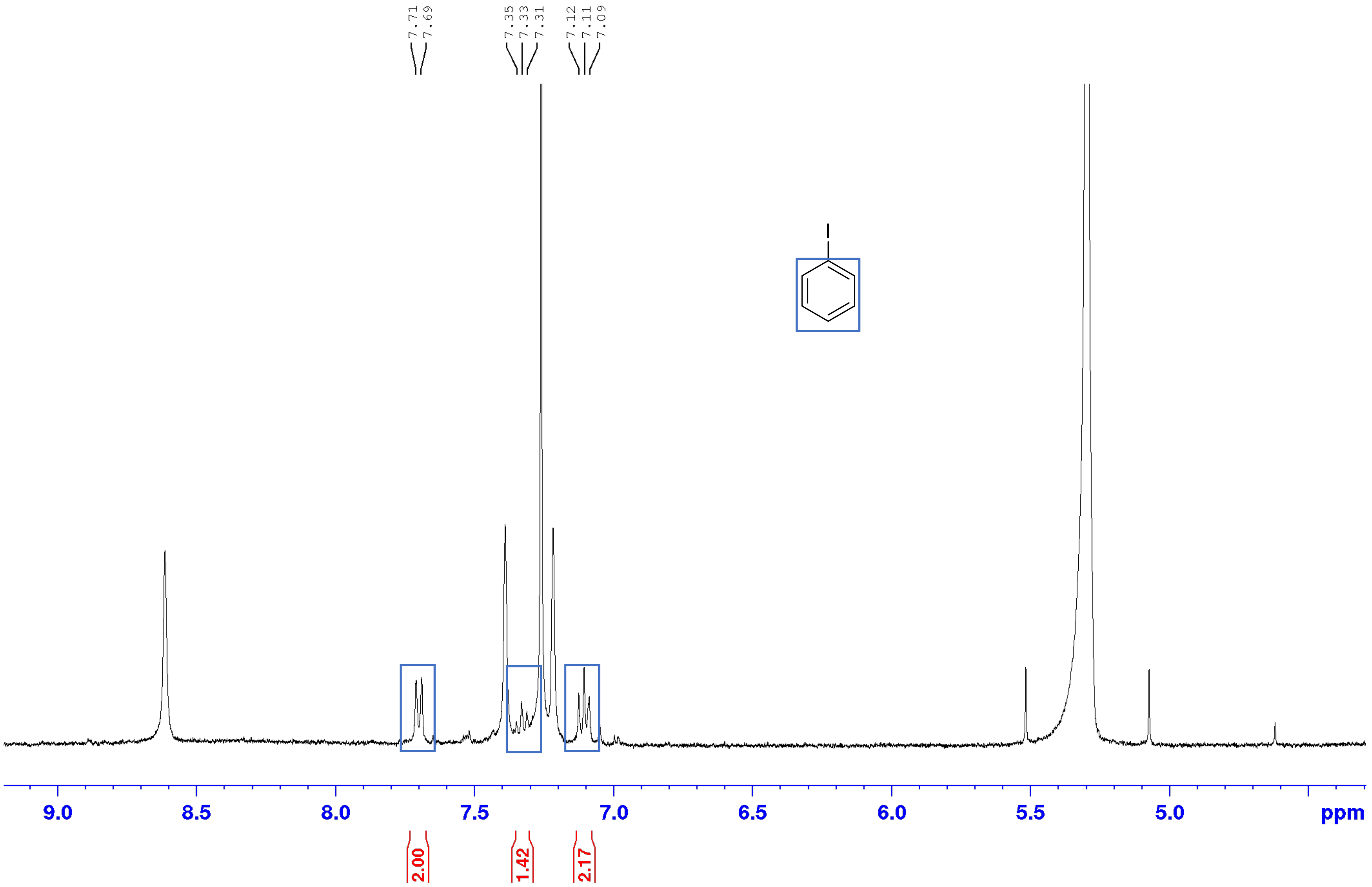

Figure S36 ¹H NMR for reaction of [Au(MeIM)$_2$(C$_6$H$_5$)(OTf)][NTf$_2$] and [NBu$_4$][I] in CDCl$_3$/CH$_2$Cl$_2$ 3:1, C$_6$H$_5$I peaks picked and integrated

[Au(MeIM)$_2$(C$_6$H$_5$)(OTf)][NTf$_2$] + [NBu$_4$][Br] – CDCl$_3$/CH$_2$Cl$_2$

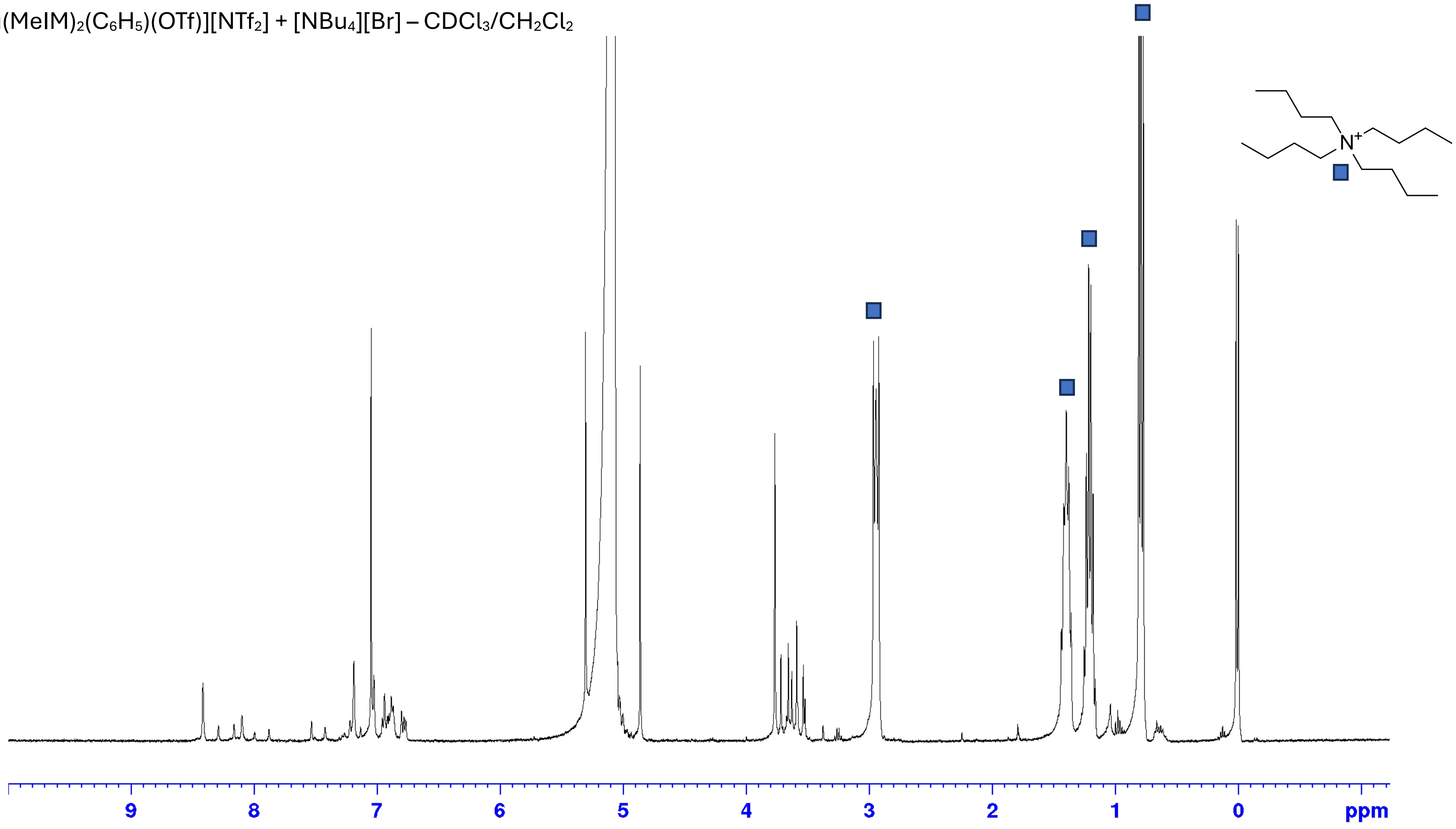

Figure S37 ^{1}H NMR for reaction of [Au(MeIM)$_2$(C$_6$H$_5$)(OTf)][NTf$_2$] and [NBu$_4$][Br] in CDCl$_3$/CH$_2$Cl$_2$ 3:1

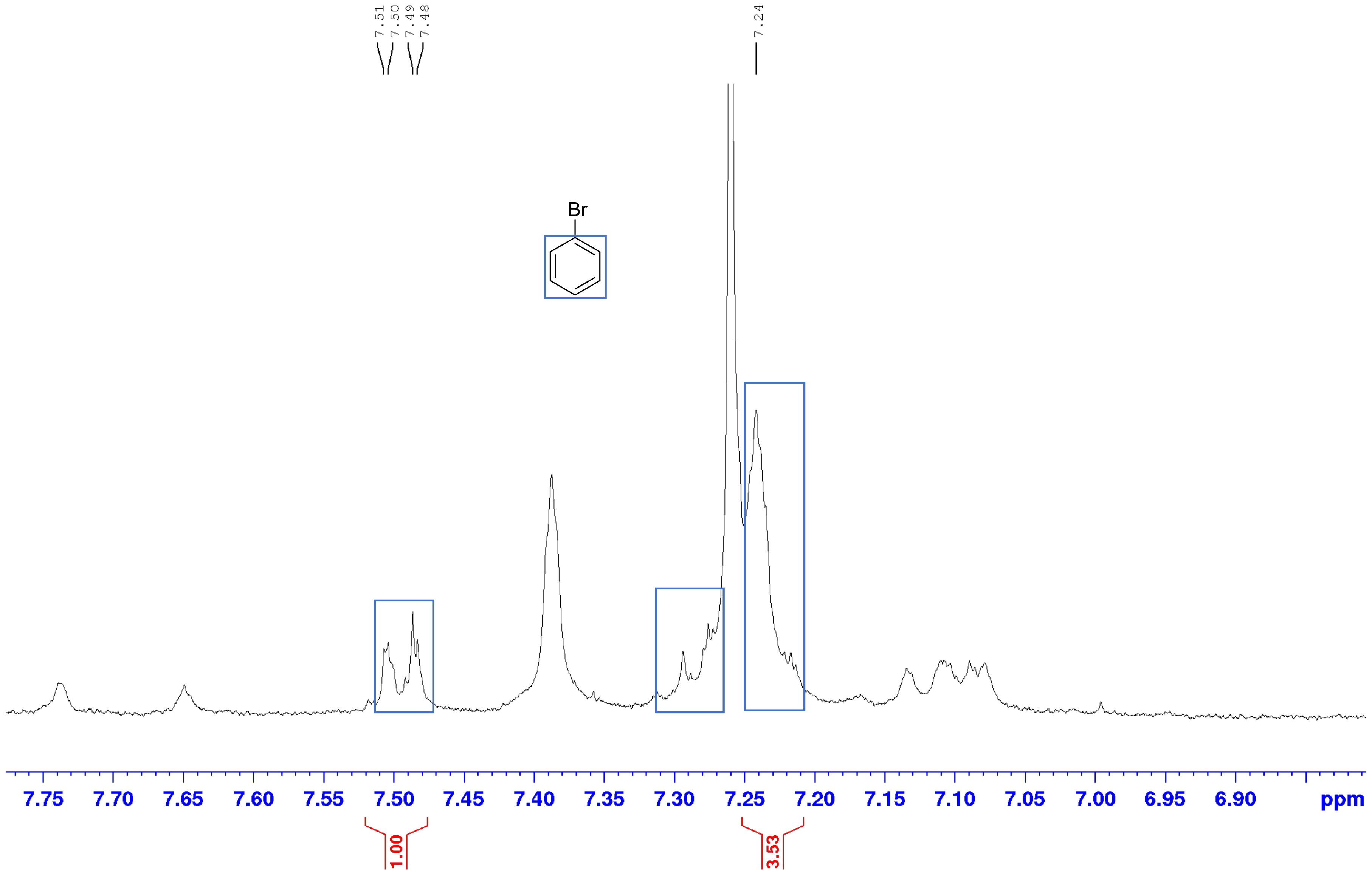

Figure S38 Close up of ^{1}H NMR of addition of [NBu$_4$][Br] to [Au(MeIM)$_2$(C$_6$H$_5$)(OTf)][NTf$_2$] in CDCl$_3$/CH$_2$Cl$_2$ 3:1

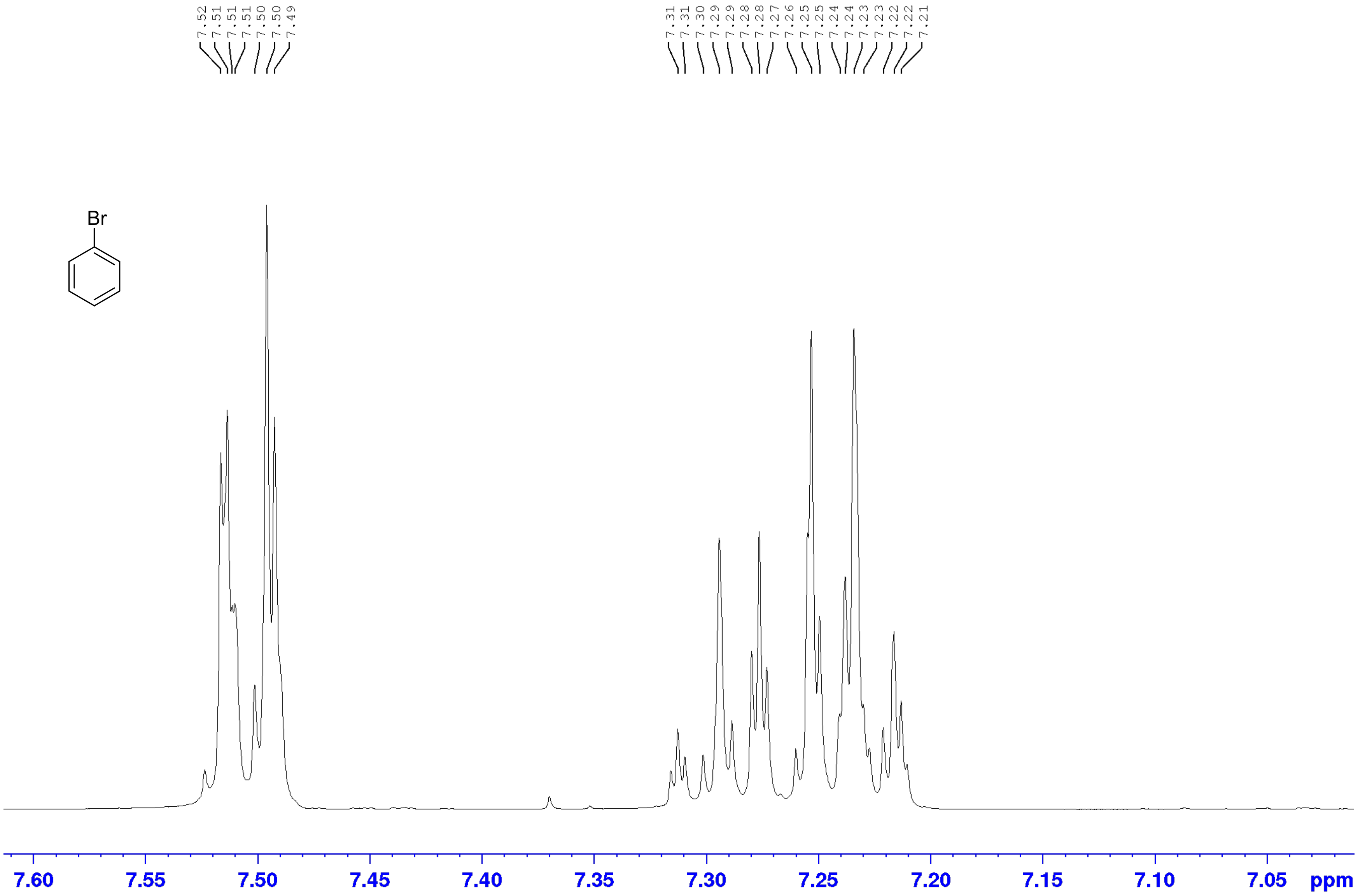

Figure S39 Reference standard of C_6H_5Br in $CDCl_3$

[Au(MeIM)$_2$(C$_6$H$_5$)(OTf)][NTf$_2$] + [NBu$_4$][Cl] – CDCl$_3$/CH$_2$Cl$_2$

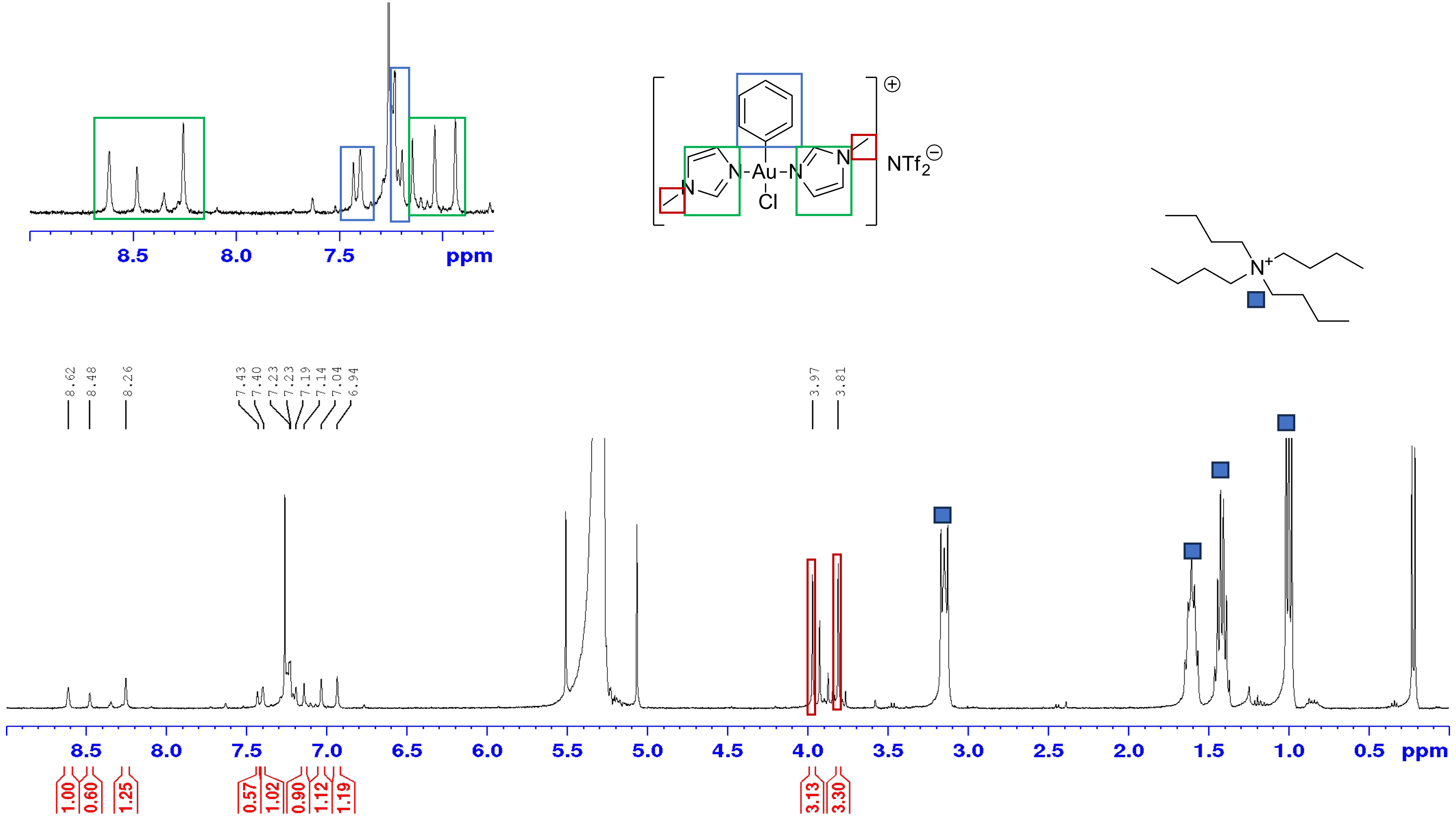

Figure S40 ^{1}H NMR of addition of [NBu$_4$][Cl] to [Au(MeIM)$_2$(C$_6$H$_5$)(OTf)][NTf$_2$] in CDCl$_3$/CH$_2$Cl$_2$ 3:1

[Au(MeIM)$_2$F$_2$][OTf] + TMS-NTf$_2$ + Mesitylene

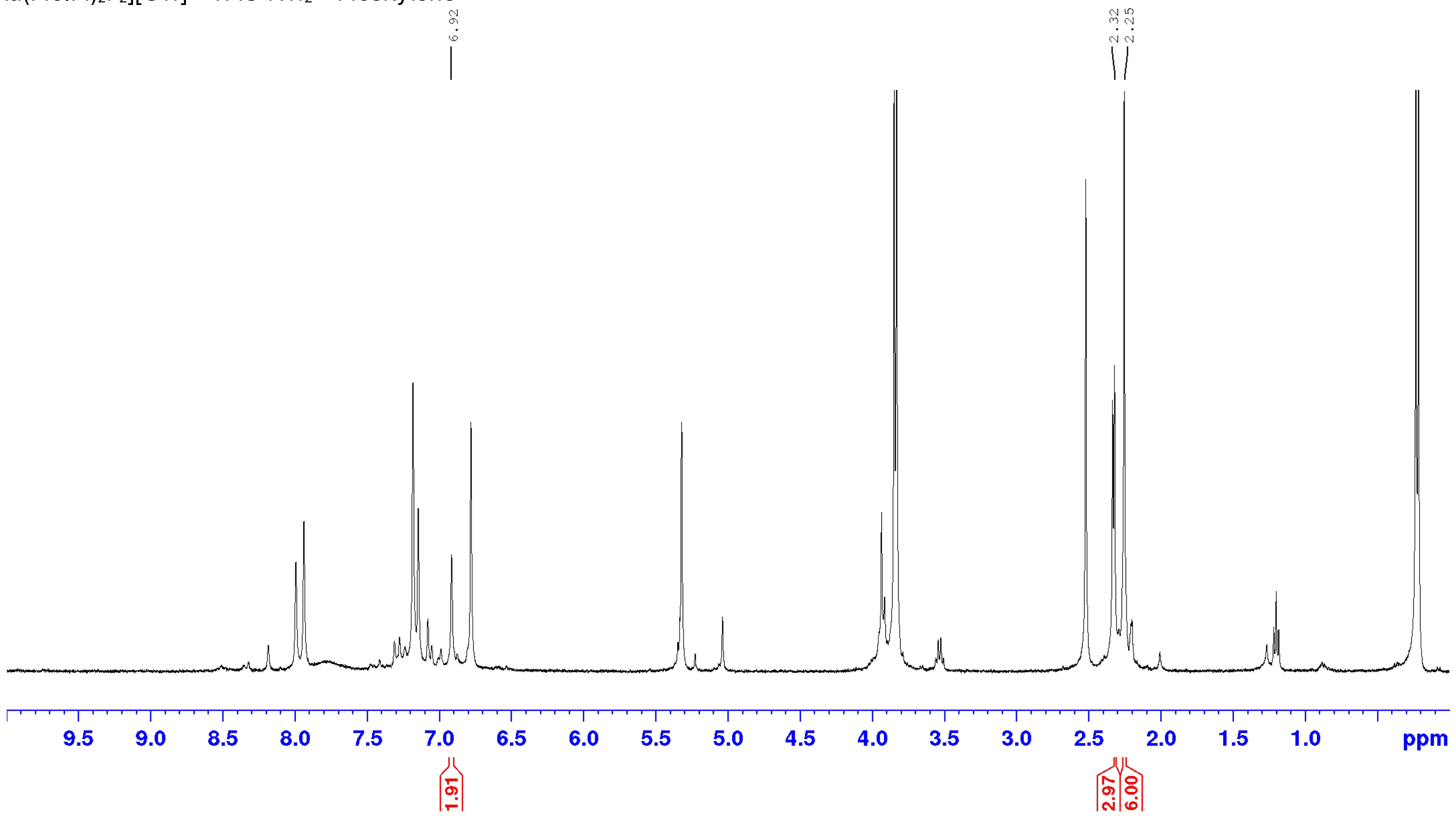

Figure S41 ^{1}H NMR of Reaction between [Au(MeIM)$_2$F$_2$][OTf] with TMS-NTf$_2$ and mesitylene

[Au(MeIM)$_2$F$_2$][OTf] + 2 TMS-OTf – CD$_3$CN

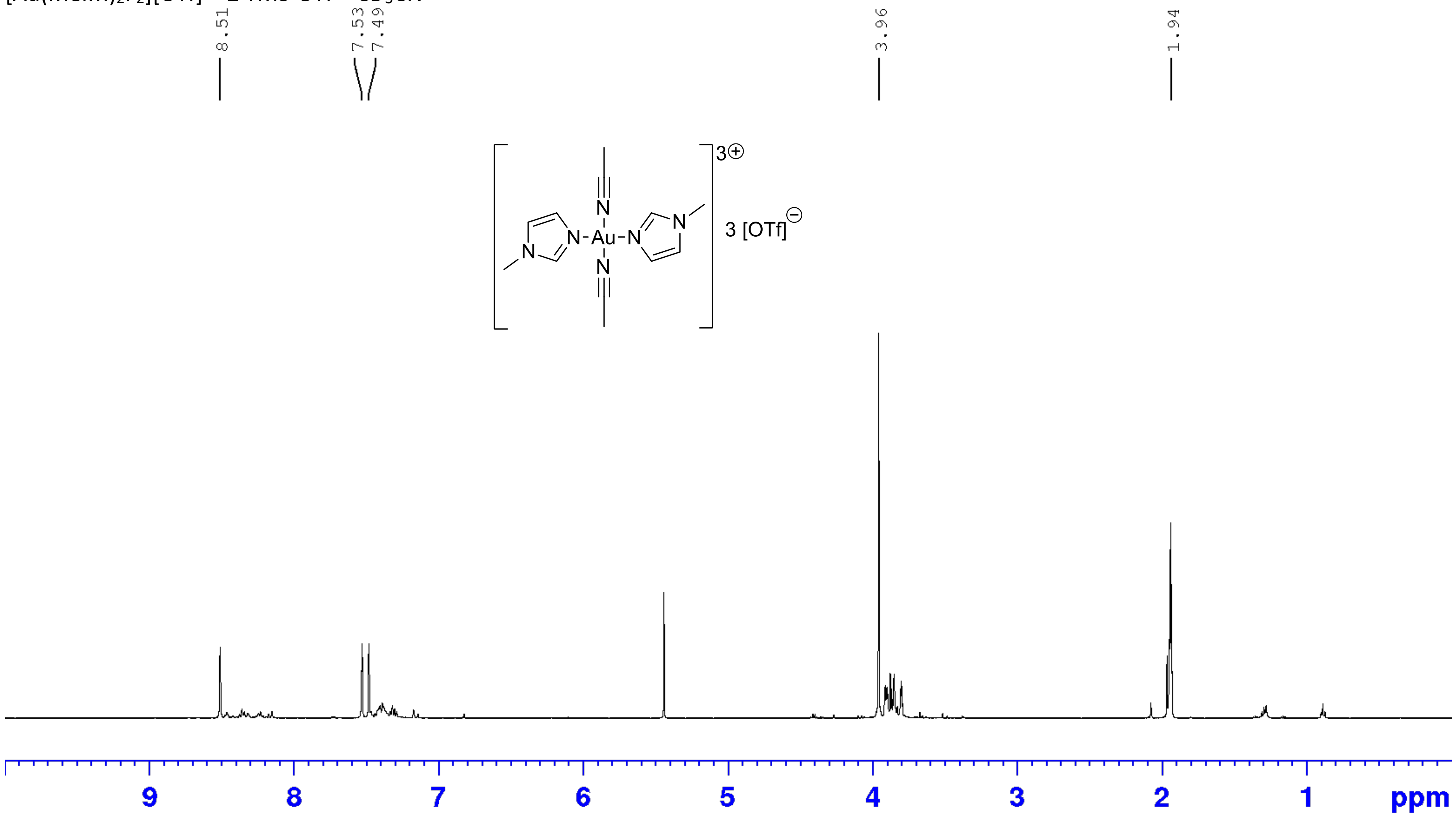

Figure S42 ¹H NMR of the reaction between [Au(MeIM)$_2$F$_2$][OTf] and TMS-OTf

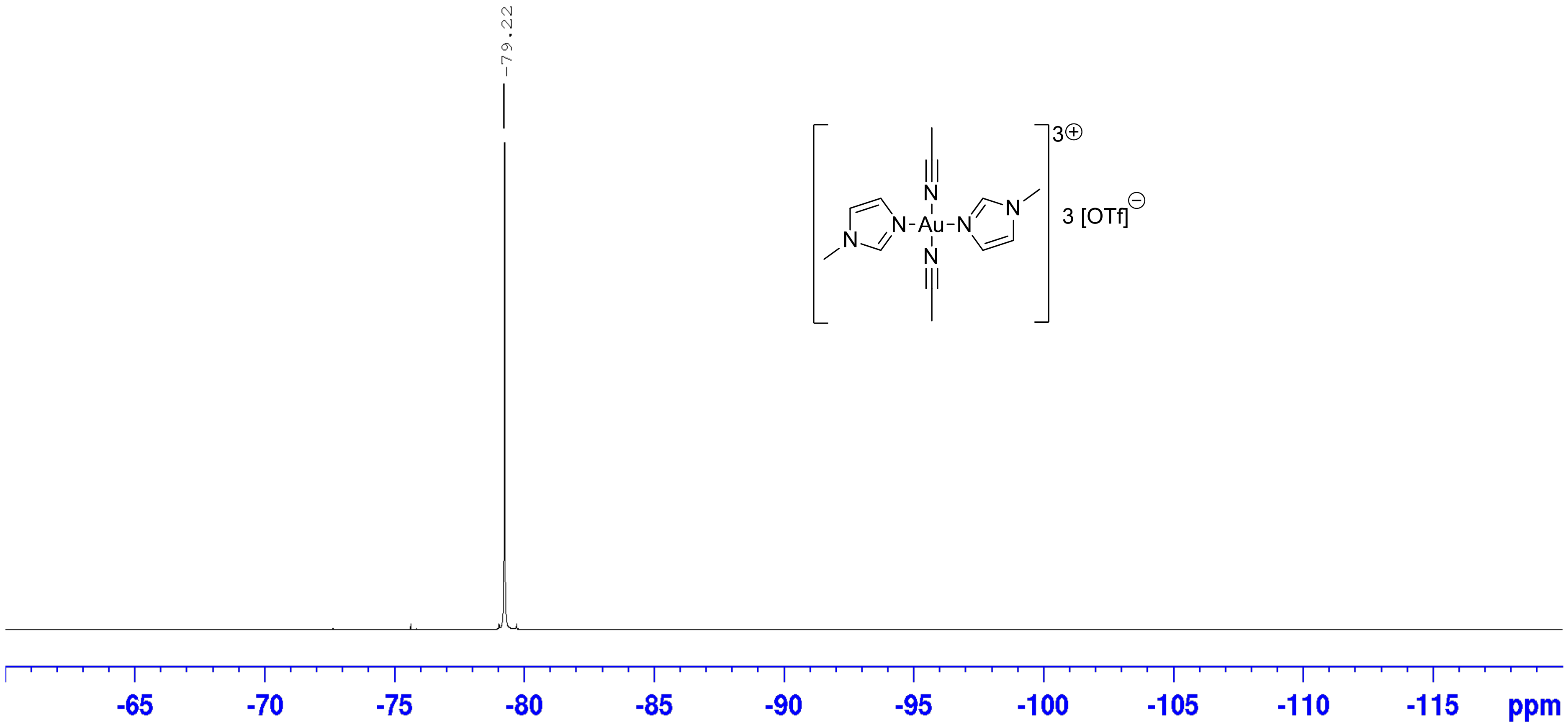

Figure S43 ^{19}F NMR of the reaction between [Au(MeIM)$_2$F$_2$][OTf] and TMS-OTf

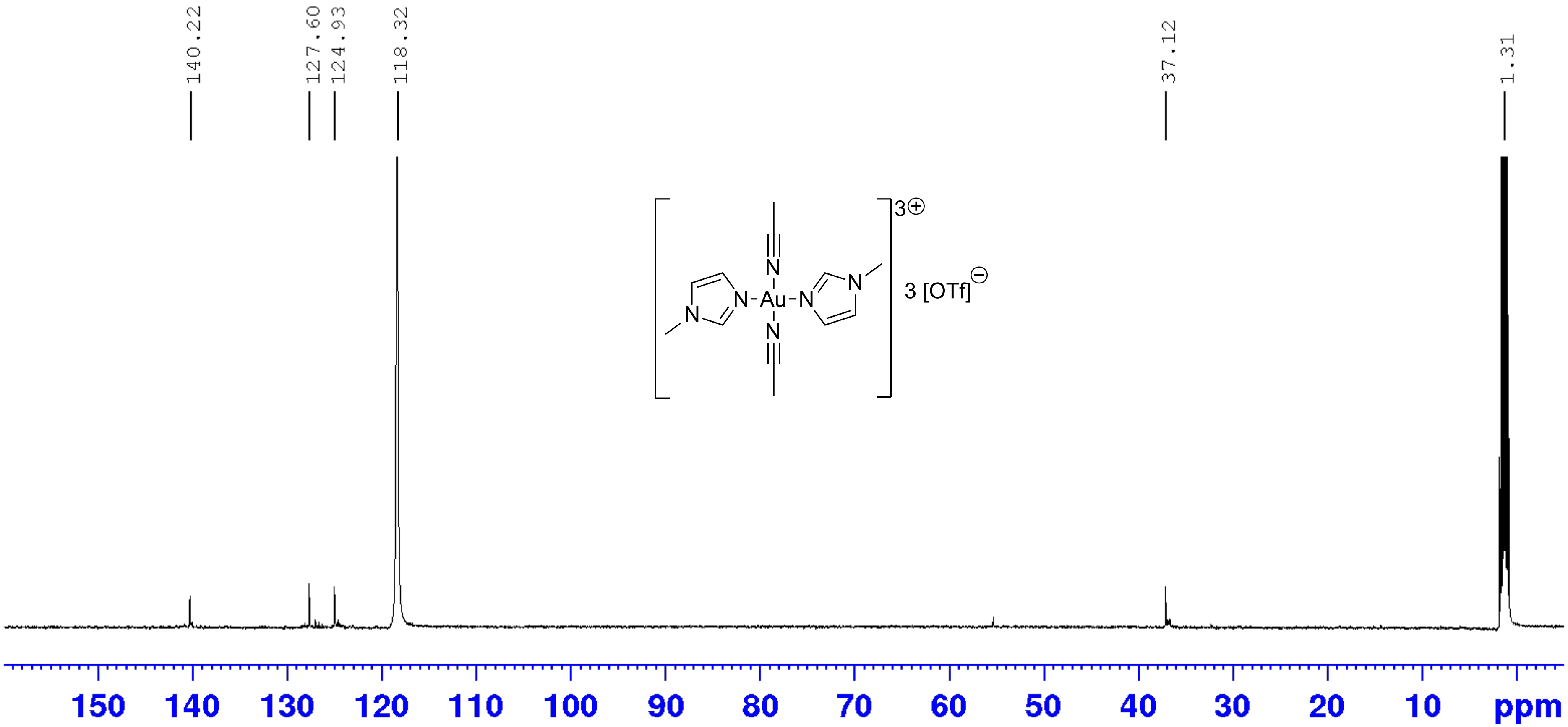

Figure S44 ^{13}C NMR of the reaction between [Au(MeIM)$_2$F$_2$][OTf] and TMS-OTf

Mass Spectra:

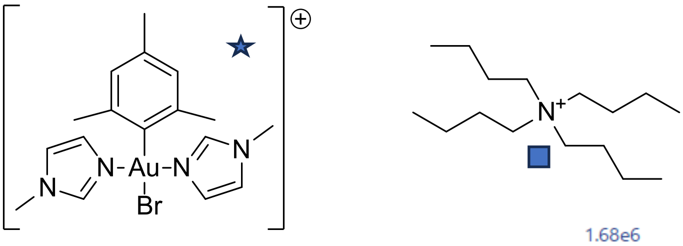

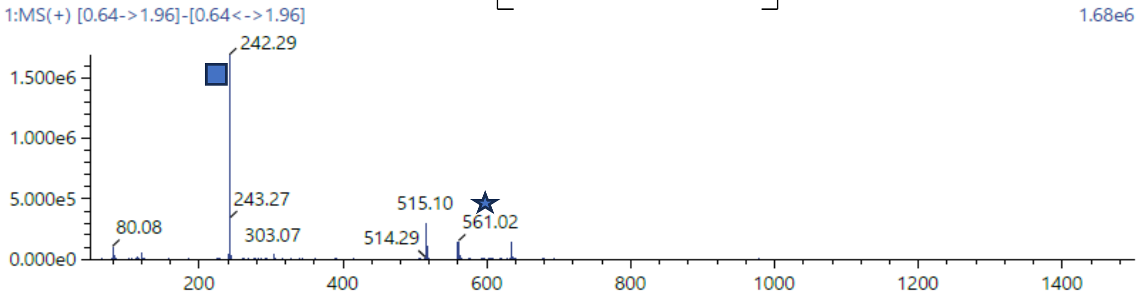

Figure S45 ESI-MS of reaction with [Au(MeIM)$_2$(MeCN)(Mes)][OTf] and [NBu$_4$][Br]

ESI-MS [Au(MeIM)$_2$(Br)(Mes)][OTf]

Calc: **560.27** m/z [Au(MeIM)$_2$(Br)(Mes)]+

Obsv: **561.02** m/z [Au(MeIM)$_2$(Br)(Mes)]+

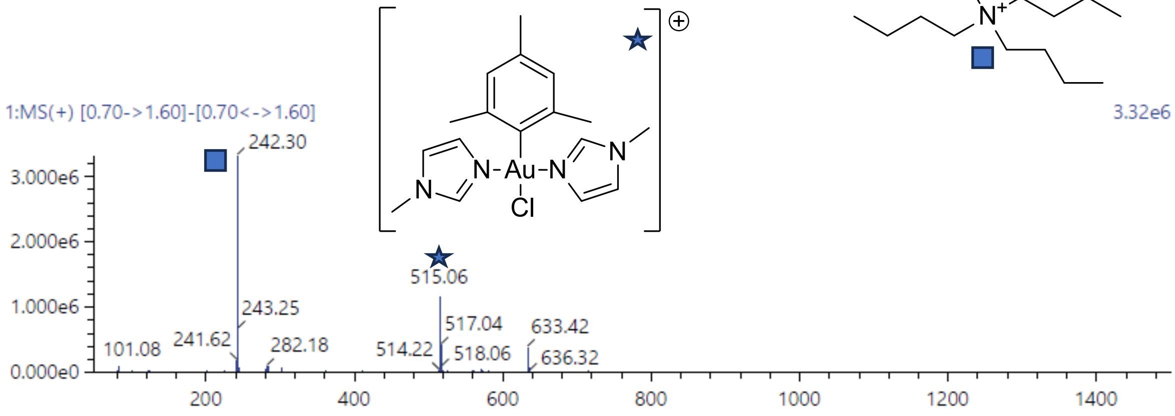

Figure S46 ESI-MS of reaction with [Au(MeIM)$_2$(MeCN)(Mes)][OTf] and [NBu$_4$][Cl]

ESI-MS [Au(MeIM)$_2$(Cl)(Mes)][OTf]

Calc: **515.81** m/z [Au(MeIM)$_2$(Cl)(Mes)]+

Obsv: **515.06** m/z [Au(MeIM)$_2$(Cl)(Mes)]+

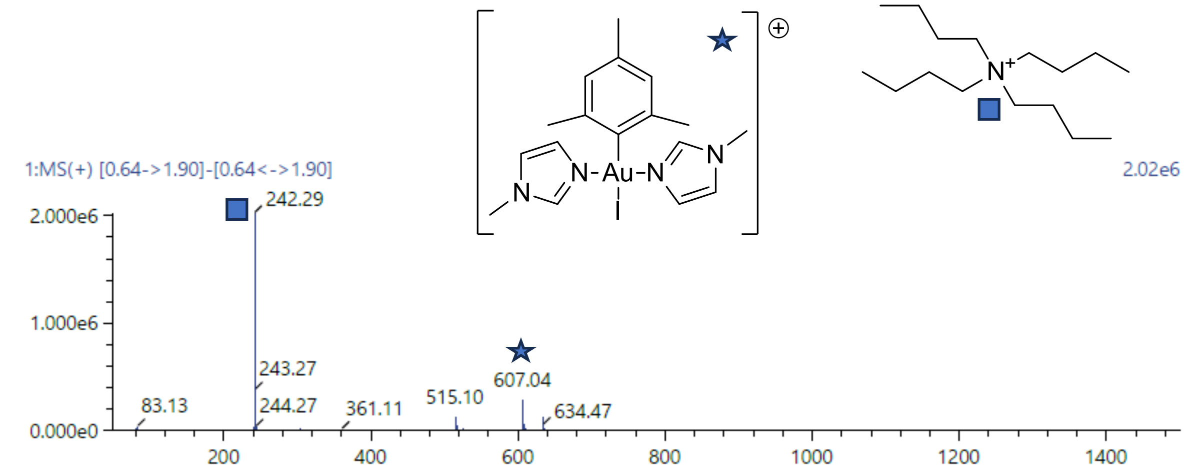

Figure S47 ESI-MS of reaction with [Au(MeIM)$_2$(MeCN)(Mes)][OTf] and [NBu$_4$][I]

ESI-MS [Au(MeIM)$_2$(I)(Mes)]OTf

Calc: **607.27** m/z [Au(MeIM)$_2$(I)(Mes)]+

Obsv: **607.04** m/z [Au(MeIM)$_2$(I)(Mes)]+

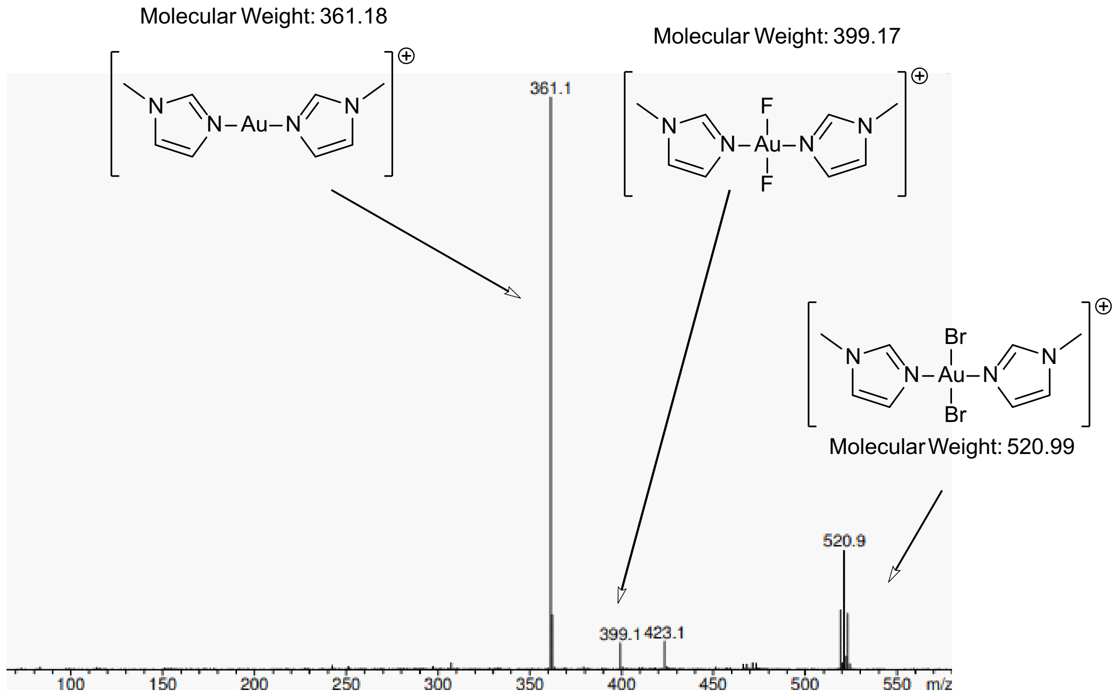

Figure S48 ESI-Ms of [Au(MeIM)$_2$Br$_2$]OTf

ESI-MS [Au(MeIM)$_2$Br$_2$][OTf]

Calc: **518.91** m/z [Au(MeIM)$_2$Br$_2$]+

Obsv: **520.9** m/z [Au(MeIM)$_2$Br$_2$]+

Computational Details:

All geometry optimisation and vibrational frequency calculations were performed using LC-ωHPBE/def2SVP using Gaussian 16 within the WebMO platform.[8-10] Molecular orbital calculations were performed on the optimized geometries using B3LYP/def2-TZVP.[11] The structures obtained are minima with no negative frequencies. Cartesian coordinates are in Å and energies are given in Hartree. Free energies were calculated at 1 atm and 298 K.

Cartesian coordinates and electronic energies for calculated species

$[Au(MeIM)_2(NTf_2)_2]^+$

Electronic Energy: -4316.49751743 Hartree

S 0.00000000 0.00000000 0.00000000

N 0.51185600 0.58377000 1.48477000

Au 2.50923700 1.00066100 1.48039300

N 2.09935200 2.94525200 1.72569700

C 1.23847400 3.62645000 0.98350400

N 1.22192200 4.89770500 1.37472200

C 0.41385500 5.96016800 0.79779800

H -0.21414100 6.41336700 1.57428500

H 1.06287300 6.72489600 0.35379600

H -0.22985400 5.53912600 0.01806700

C 2.11130600 5.03593700 2.41406100

C 2.65965300 3.81318700 2.63189200

H 3.38887200 3.49774800 3.37436500

H 2.27627000 5.98666500 2.91564000

H	0.63833000	3.21331100	0.17454800
N	4.48012700	1.42771200	1.44769200
S	5.26420500	1.66058600	0.00746600
O	6.65486600	1.34183300	0.18218600
C	5.16514600	3.48673000	-0.26094900
F	5.56803000	4.12737800	0.81272900
F	5.92147100	3.78556500	-1.28979800
F	3.90368100	3.81415400	-0.52052700
O	4.42683700	1.07390500	-1.01441400
S	5.30641700	1.36382500	2.90161600
O	6.22932500	2.45536800	3.01999000
O	4.25381100	1.11666300	3.86594900
C	6.32175600	-0.20636700	2.80505400
F	6.15661600	-0.83772900	3.94675600
F	7.57712500	0.09794400	2.62531200
F	5.88520300	-0.97014400	1.81342200
N	2.89923400	-0.94358600	1.20567500
C	3.18434900	-1.50899700	0.03855700
N	3.35683500	-2.81496000	0.21383800
C	3.66057400	-3.78208800	-0.82852900
H	2.83422600	-4.49692600	-0.92486100
H	3.79211000	-3.25517800	-1.77955700
H	4.58701600	-4.31494700	-0.58288500
C	3.17609200	-3.09928800	1.54689000

C 2.88910900 -1.92766100 2.16826700

H 2.66531600 -1.71510200 3.21137900

H 3.26136800 -4.10876300 1.94184600

H 3.27610000 -0.97907100 -0.90940200

S -0.14615400 0.07126300 2.93606400

O 0.96146300 -0.19603400 3.82358400

O -1.22399400 -0.84486400 2.69795300

C -0.90165700 1.63910700 3.55842700

F -1.67490700 2.16447600 2.62320800

F -1.60782800 1.35539900 4.62463100

F 0.05629800 2.50066800 3.86119400

O -0.22413700 -1.41788600 0.00332600

C -1.62671400 0.82941900 -0.29795500

F -1.98986500 0.50712200 -1.51700900

F -2.52787800 0.44552700 0.56927100

F -1.45158200 2.14398900 -0.21612000

O 0.91937200 0.63008400 -0.93043600

$[Au(MeIM)_2F_2]^+$

Electronic Energy: -865.333357645 Hartree

Au 0.00000000 0.00000000 0.00000000

F -0.00004700 1.91275400 0.00030000

N 1.99155300 0.00442400 -0.00014700

C 2.75015600 1.09062200 -0.00031300

N 4.03263300 0.73287100 -0.00002900

C 5.17313500 1.63401100 -0.00011600

H 5.78181200 1.46713300 0.89697200

H 5.78256900 1.46595800 -0.89646600

H 4.81399700 2.66871800 -0.00094500

C 4.09366300 -0.64209500 0.00039200

C 2.81339300 -1.09570300 0.00033800

H 2.40931400 -2.10540000 0.00052600

H 5.03904500 -1.17955800 0.00065900

H 2.36911000 2.11132500 -0.00067000

F 0.00010200 -1.90215600 -0.00017300

N -1.99155600 0.00440300 0.00007600

C -2.75017000 1.09061600 0.00010400

N -4.03262200 0.73286000 0.00003400

C -5.17314300 1.63399000 0.00008600

H -5.78249700 1.46605000 0.89651000

H -4.81401400 2.66869900 0.00072100

H -5.78188900 1.46696100 -0.89692700

C -4.09367100 -0.64210400 -0.00014300

C -2.81342300 -1.09573600 0.00002400

H -2.40919200 -2.10538100 -0.00004900

H -5.03908700 -1.17950600 -0.00020600

H -2.36898500 2.11127400 0.00028100

$[Au(MeIM)_2(OAc)_2]^+$

Electronic Energy: -1122.24248653 Hartree

Au 0.00000000 0.00000000 0.00000000

O 0.14725700 1.85018200 0.64907800

C 0.58349000 2.86686200 -0.11754400

O 1.33416900 3.67784400 0.35059400

C 0.08635200 2.90841500 -1.53894600

H -0.99303900 2.71587500 -1.59471400

H 0.31386700 3.88736000 -1.97370500

H 0.59879900 2.14064400 -2.13843700

N -1.99916000 0.12845200 0.00016800

C -2.70313700 1.06570900 0.62101300

N -4.00247000 0.84625400 0.42928000

C -5.09415600 1.65173200 0.95292500

H -5.69109100 2.05499200 0.12581600

H -5.73101500 1.04000200 1.60351000

H -4.68158800 2.48270200 1.53512400

C -4.13536900 -0.27897800 -0.35047400

C -2.88098800 -0.72718800 -0.61710400

H -2.54089400 -1.58306700 -1.19685600

H -5.10673000 -0.66504000 -0.65097500

H -2.27520400 1.88667500 1.19597700

O -0.12455600 -1.83968300 -0.63993500

C -0.18479000 -2.92663100 0.15837200

O -0.26846100 -4.00830300 -0.34249100

C -0.14201100 -2.68775300 1.64747000

H -0.99226800 -2.07028700 1.97171800

H -0.19107000 -3.65354100 2.16097200

H 0.78649000 -2.17621400 1.93954500

N 2.00106100 -0.11648500 0.00638100

C 2.81898800 0.75063200 0.59480000

N 4.07911500 0.37179900 0.39745400

C 5.26162700 1.06605100 0.88209500

H 5.91001100 1.32891100 0.03760600

H 4.95325000 1.98410000 1.39379100

H 5.81025400 0.42684700 1.58471300

C 4.07065700 -0.78538300 -0.34635200

C 2.77001900 -1.08933400 -0.59064700

H 2.32675100 -1.91401000 -1.14510400

H 4.98633700 -1.29250100 -0.64151600

H 2.50812700 1.65002700 1.13100500

$[Au(MeIM)_2(OTf)_2]^+$

Electronic Energy: -2586.78371724 Hartree

S 0.00000000 0.00000000 0.00000000

O -1.24279500 0.13247700 0.97175200

Au -3.03131000 -0.23044200 0.21968200

N -3.48926900 1.71003200 0.37214700

C -4.39350600 2.34552600 -0.36170500

N -4.44723900 3.62197200 0.00711800

C -5.31478500 4.64196000 -0.56105400

H -5.97011600 5.04930000 0.21837000

H -4.70937500 5.44712100 -0.99479200

H -5.93041800 4.19337900 -1.34801300

C -3.54138400 3.81271000 1.02420400

C -2.94216100 2.61578000 1.24956600

H -2.16596000 2.34002200 1.95890900

H -3.39859400 4.77997300 1.50030600

H -5.01044200 1.88932600 -1.13487800

O -4.82453700 -0.55170700 -0.53201800

S -5.86992900 -1.39479400 0.30720500

O -6.45455900 -2.39973700 -0.53292600

O -5.29772000 -1.69094600 1.60104700

C -7.14009300 -0.08924600 0.56106500

F -8.16814000 -0.60377400 1.19040400

F -7.51547600 0.39938600 -0.60787000

F -6.61006300 0.88992300 1.28702800

N -2.57120500 -2.17013800 0.05902700

C -1.67409800 -2.81587500 0.79572200

N -1.62967200 -4.08995600 0.42224700

C -0.76053300 -5.11272600 0.98258100

H -0.13167600 -5.53761700 0.19107400

H -0.11619500 -4.65865500 1.74277600

H -1.36416300 -5.90365400 1.44418000

C -2.53867700 -4.27324400 -0.59284200

C -3.12784600 -3.07256300 -0.81948100

H -3.90785300 -2.79980300 -1.52633500

H -2.69241300 -5.23888800 -1.06857900

H -1.04644400 -2.37201500 1.56656600

O -0.47127100 0.03809500 -1.36603600

C 0.81066500 1.61105100 0.36215700

F 1.94144700 1.66669500 -0.30174700

F 0.00819200 2.58984200 -0.02738900

F 1.03288200 1.71246400 1.65695600

O 0.87599100 -1.03785000 0.47639000

$[Au(MeIM)_2]^+$

Electronic Energy: -665.969770595 Hartree

C 0.00000000 0.00000000 0.00000000

N 1.20893400 -0.54358500 -0.59238600

C 2.46204600 -0.16214100 -0.32585800

N 3.32348100 -0.86448800 -1.04289100

C 2.58561800 -1.73789300 -1.80567700

C 1.26837400 -1.54352800 -1.53002300

H 0.37651000 -2.02852100 -1.91959200

H 3.04705900 -2.44014700 -2.49545100

Au 5.34299900 -0.68368600 -1.03314800

N 7.36251700 -0.50300400 -1.04431500

C 8.22397600 -1.19959700 -0.32171700

N 9.47707000 -0.82022500 -0.59126800

C 10.6860340 -1.35910500 0.00534200

H 10.4160760 -2.13858800 0.72613500

H 11.3257080 -1.79828700 -0.77019200

H 11.2343870 -0.56578300 0.52826800

C 9.41758900 0.17222600 -1.53683200

C 8.10033800 0.36435300 -1.81401200

H 7.63886200 1.06110400 -2.50932400

H 10.3094280 0.65416000 -1.93023900

H 7.96897700 -1.97720200 0.39663500

H 2.71707600 0.62115400 0.38627400

H -0.54787700 -0.78894300 0.53000900

H 0.26994200 0.78583300 0.71387000

H -0.64011400 0.43219500 -0.77908500

$[NTf_2]^-$

Electronic Energy: -1825.38542253 Hartree

S 0.00000000 0.00000000 0.00000000

N -1.12899500 0.84588700 -0.72538700

S -2.25770000 1.69213200 0.00000600

O -1.97784600 2.08329000 1.36994400

O -2.78980700 2.67752800 -0.92139700

C -3.62179600 0.46547600 0.15077600

F -4.63598000 1.00835800 0.82522700

F -3.22565800 -0.62331900 0.79688800

F -4.07123600 0.10178000 -1.04652800

O -0.27972800 -0.39132700 1.36990800

C 1.36379000 1.22700300 0.15079700

F 0.96753600 2.31563800 0.79709900

F 2.37813900 0.68416800 0.82507600

F 1.81299800 1.59092600 -1.04651600

O 0.53242700 -0.98517500 -0.92146700

[OTf]$^{-}$

Electronic Energy: -960.520784958 Hartree

S 0.00000000 0.00000000 0.00000000

O 0.30926200 -1.35758500 0.45452800

O 0.30942000 1.07225300 0.94821600

O 0.30945000 0.28510500 -1.40275900

C -1.84359600 -0.00058900 0.00020300

F -2.34170900 1.17888600 -0.39467900

F -2.34053400 -0.24791900 1.21859200

F -2.34041900 -0.93179100 -0.82382400

[OAc]$^{-}$

Electronic Energy: -228.193886903 Hartree

C 0.00000000 0.00000000 0.00000000

C -1.56010300 -0.05383100 0.00002100

O -2.03787700 -1.20488700 -0.00000900

O -2.14340000 1.04601700 -0.00001100

H 0.38658300 -0.53233800 -0.88370700

H 0.37094000 1.03533400 0.00046700

H 0.38667000 -0.53320000 0.88314900

References

1 Corbo, R., Ryan, G. F., Haghighatbin, M. A., Hogan, C. F., Wilson, D. J. D., Hulett, M. D., Barnard, P. J., Dutton, J. L., *Inorg. Chem.,* **2016**, *55* (6), 2830-2839

2 Barwise, L., Moon, L, J., Dhakal, B., Hogan, C, F., White, K, F., Dutton. J.L., *Chem. Commun*., **2024**,60, 5586-5589

3 Albayer, M., Corbo, R., Dutton, J. L., *Chem. Commun*., **2018**, 54, 6832-6834

4 Cong-Ying, Z., Jing, L., *Organic Letters*, **2010**, 12(9), 2104-2107,

5 Sheldrick, G., *Acta Crystallogr. A* **2015**, 71, 3–8.

6 Sheldrick, G., *Acta Crystallogr. C* **2015**, 71, 3–8.

7 Dolomanov, O., Bourhis L., Gildea, R., Howard, J., Puschmann H., *J. Appl. Cryst*. **2009**, 42, 339–341

8 Heyd. J., Scuseria, G. E., Ernzerhof, M., *J. Chem. Phys.* **2003**, 118. 8207-8215

9 Heyd. J., Scuseria, G. E., *J. Chem. Phys.* **2004**, 120. 7274-7280

10 Weigend, F., Ahlrichs, R. *Phys. Chem. Chem. Phys.* **2005**, *7*, 3297-3305

11 Becke A. D., *J. Chem. Phys.* **1993**, *98*, 5648-5652
